# Nanotechnology-based ocular drug delivery systems: recent advances and future prospects

**DOI:** 10.1186/s12951-023-01992-2

**Published:** 2023-07-22

**Authors:** Shiding Li, Liangbo Chen, Yao Fu

**Affiliations:** 1 Department of Ophthalmology, Ninth People’s Hospital, Shanghai Jiao Tong University School of Medicine, Shanghai, China; 2 Shanghai Key Laboratory of Orbital Diseases and Ocular Oncology, Shanghai, 200011 China

**Keywords:** Nanotechnology, Ocular drug delivery systems, Ocular barriers, Characterizations, Clinical trail

## Abstract

Ocular drug delivery has constantly challenged ophthalmologists and drug delivery scientists due to various anatomical and physiological barriers. Static and dynamic ocular barriers prevent the entry of exogenous substances and impede therapeutic agents' active absorption. This review elaborates on the anatomy of the eye and the associated constraints. Followed by an illustration of some common ocular diseases, including glaucoma and their current clinical therapies, emphasizing the significance of drug therapy in treating ocular diseases. Subsequently, advances in ocular drug delivery modalities, especially nanotechnology-based ocular drug delivery systems, are recommended, and some typical research is highlighted. Based on the related research, systematic and comprehensive characterizations of the nanocarriers are summarized, hoping to assist with future research. Besides, we summarize the nanotechnology-based ophthalmic drugs currently on the market or still in clinical trials and the recent patents of nanocarriers. Finally, inspired by current trends and therapeutic concepts, we provide an insight into the challenges faced by novel ocular drug delivery systems and further put forward directions for future research. We hope this review can provide inspiration and motivation for better design and development of novel ophthalmic formulations.

**Graphical abstract:**

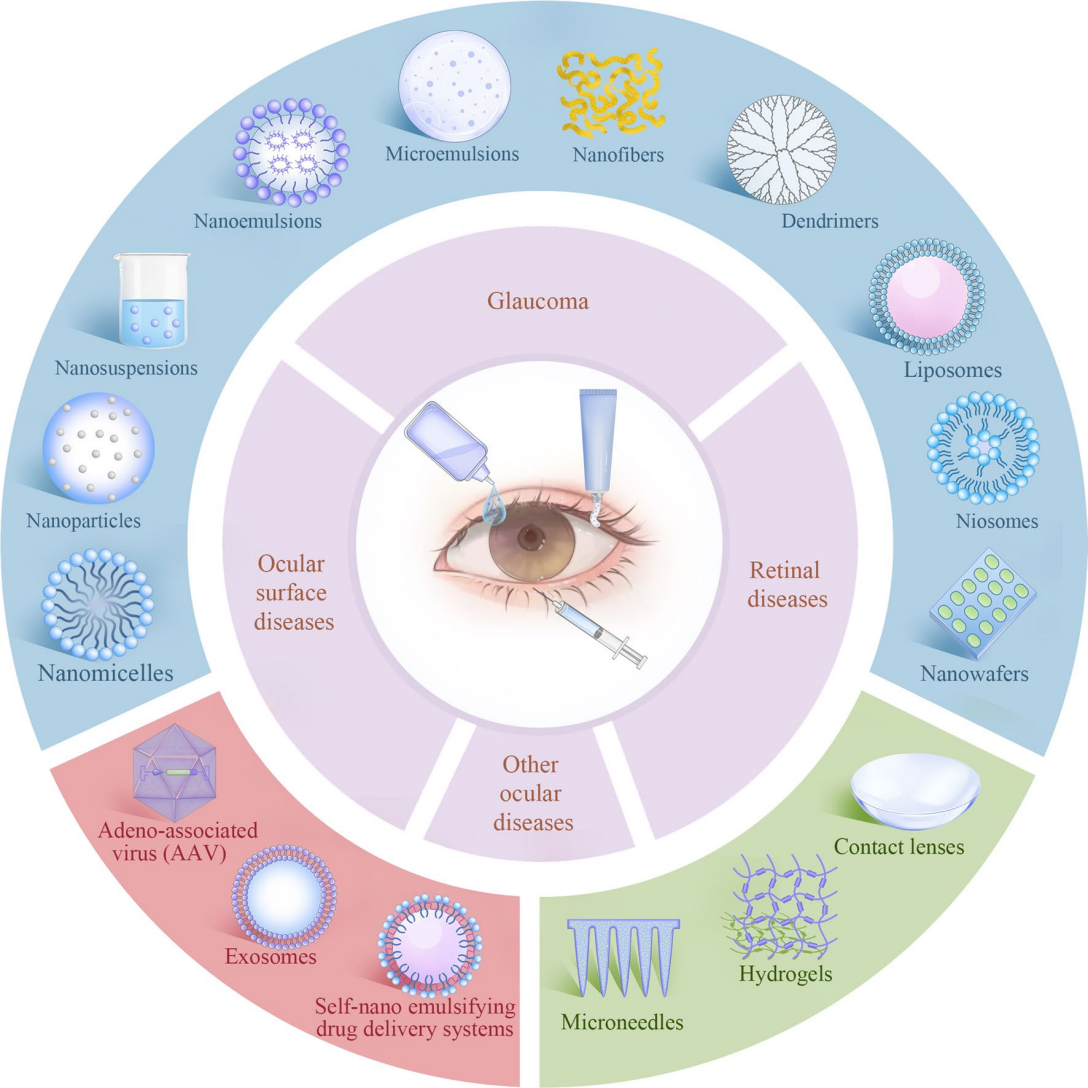

## Introduction

The eye, a highly complex, isolated and specialized organ, is the most significant sensory organ of the human body because about 80% of all sensory input is acquired via the eye [1]. Anatomically, ocular tissues are protected by dynamic and static barriers [2]. Tear turnover, reflex blinking, and nasolacrimal drainage prevent foreign substances away from the eye surface [2, 3]. The eyelid, conjunctiva and corneal epithelium cover and protect the eye surface [4]. In addition, the blood- aqueous barriers (BAB) and blood-retina barriers (BRB) limit the entry of compounds from the systemic circulation [5]. This defense system is further assisted by enzymes and other barriers (sclera, retinal etc.) [6, 7].

Although there are multiple protective mechanisms, the eyeball is still vulnerable to infection, trauma and other injuries due to its communication with the outside [8]. The World Health Organization reports that at least 2.2 billion people around the world have visual impairment [9]. Ocular diseases, such as keratitis [10], cataract [11], glaucoma [12], age-related macular degeneration (AMD) [13] and diabetic retinopathy (DR) [14] can seriously damage the patients' visual acuity and affect their life quality. The National Eye Institute estimated that the annual economic burden associated with eye conditions and vision impairment in the US is about $139 billion [15].

Drug therapy is the primary treatment for most eye diseases [16]. Delivering drugs to target eye tissues at the desired therapeutic concentration without damaging healthy tissues is a current research hotspot [17]. Ocular drug delivery systems (ODDS) are designed to: (1) overcome ocular barriers to deliver drugs to target eye tissues, (2) improve drug stability and treatment efficiency, (3) prolong drug retention time and reduce dosing frequency, (4) enable multiple drug combinations, and (5) improve patient adherence and reduce drug-related adverse events [18, 19].

Traditional administration methods, such as topical eye drops, conjunctival and scleral administration, intracameral administration, intravitreal injection, retrobulbar injection and systemic administration, are widely used clinically and have achieved certain therapeutic effects [20]. However, as mentioned earlier, the presence of ocular barriers poses a significant challenge for therapeutics in terms of reaching the intended site and staying there for a sufficient duration. As a result, the bioavailability of these therapeutics is often limited, typically less than 5% [21].

With the development of nanotechnology, dynamic progress has been made in the field of ocular drug delivery, which provides new therapeutic interventions for ocular diseases [21, 22]. Compared with traditional drug administration, nanocarriers offer numerous advantages, including the capacity to overcome ocular barriers, promote transcorneal permeability, prolong drug residence time, reduce drug degradation, reduce dosing frequency, improve patient compliance, achieve sustained/controlled release, drug targeting and gene delivery [23]. Novel drug carriers, such as nanomicelles, nanoparticles (NPs), nanoemulsions (NEs), microemulsions, nanofibers, dendrimers, liposomes, niosomes, nanowafers, microneedles (MNs), have been investigated for the therapy of anterior and posterior ocular diseases [24].

In this review, we attempted to provide a holistic overview of novel ODDS reported in the past five years. First, we described the specific anatomy of the eye and the ocular barriers, illustrating the key factors that lead to the low bioavailability of the therapeutics. Subsequently, based on the current treatment status of ophthalmic diseases, several conventional and alternative routes of administration were summarized and compared, especially their limitations and innovative progress. Then, we discussed the recent advances in novel nanocarriers, such as nanomicelles, NPs, nanosuspensions, microemulsions, dendrimers, liposomes, etc. and highlighted some recent research. In particular, we also introduced gene therapy, exosome and self-nano emulsifying drug delivery systems (SNEDDS), which have huge potential in ocular drug delivery. In view of the reports of these ODDS, we highlighted their characteristics to assist with future related research. Meanwhile, ophthalmic drugs currently on the market or still in clinical trials were summarized, as well as the recent patents of nanocarriers. Finally, inspired by current trends and therapeutic concepts, we focused on novel non-invasive ODDS to overcome ocular barriers, sustain drug release, and maintain effective drug levels at the therapeutic target. Although most current research is still in the basic research stage, ocular drug delivery based on nanotechnology is expected to become the main means of ocular drug therapy.

## The anatomy and barriers of the eye

**Fig. 1:**
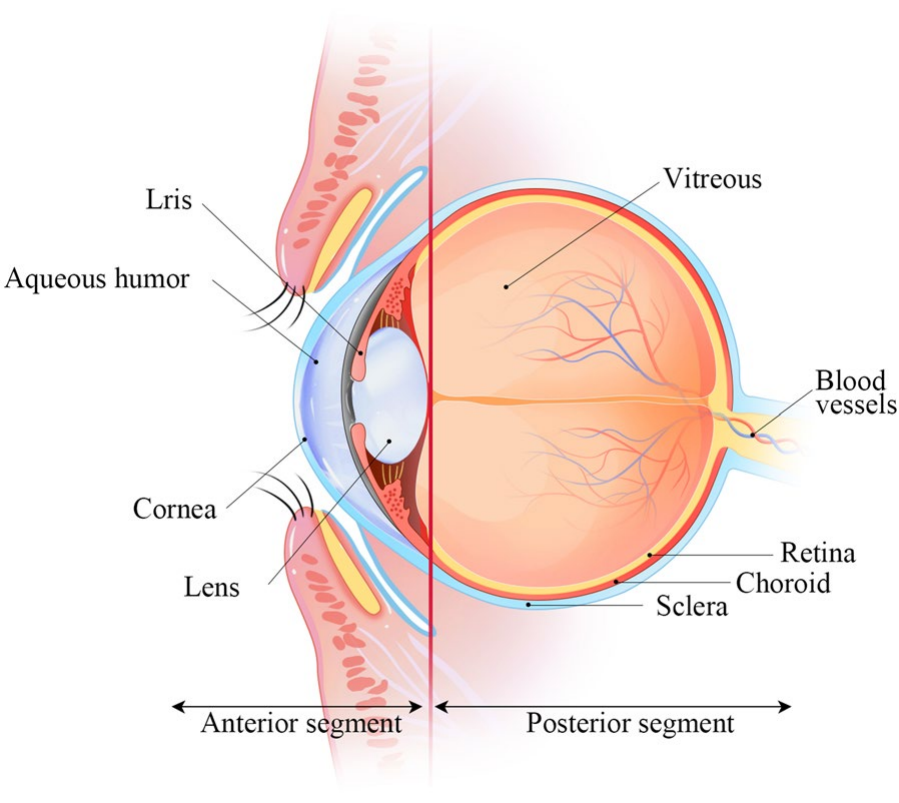
The anatomy of the eye

The anatomical structure of the eyeball can be divided into the anterior and posterior segments based on the lens. Figure 1 illustrates the anatomy of the human eye. The anterior segment includes the cornea, conjunctiva, iris, ciliary body, aqueous humor and lens, while the posterior segment includes the sclera, choroid, retina and vitreous body [25, 26].

**Fig. 2:**
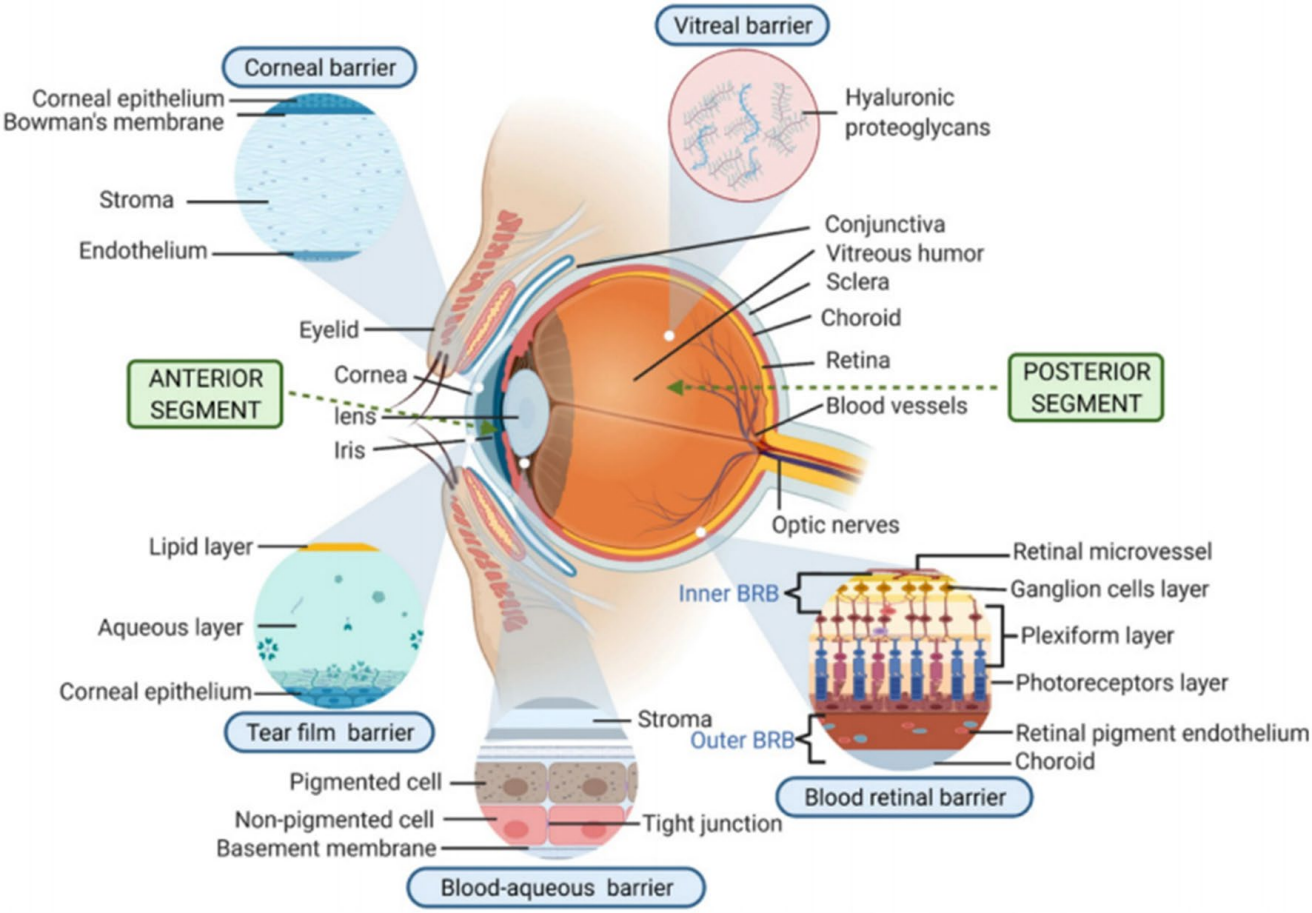
Drug delivery barriers in ocular routes [26]. The absorption barriers of the eye mainly include tear film barrier, corneal barrier, conjunctival and scleral barriers, vitreal barrier, blood-aqueous barrier, blood-retinal barrier. Copyright 2022, Drug Delivery and Translational Research

Various absorption barriers exist in the human eye (Fig. 2) [27]. They are briefly divided into static and dynamic barriers to prevent foreign substances, including therapeutic agents, from targeting various eye tissues [28]. Static barriers of the eye mainly include cornea, conjunctiva, sclera, vitreal barrier, BAB and BRB, while dynamic barriers primarily include tear film, tear turnover, nasolacrimal duct drainage, conjunctival and choroidal blood flow and lymphatic clearance [29–31]. These barriers limit the passive absorption of diverse therapeutic molecules, thereby reducing the ocular bioavailability of different agents. Details are described below to understand the absorption barriers further.

### Tear film, tear turnover, nasolacrimal duct drainage

The tear film is a thin, transparent fluid layer consisted of three layers: a surface lipid layer, an intermediate aqueous layer, and an inner mucin layer [32]. The lipid and water layers act as barriers for hydrophilic and hydrophobic drugs, respectively [33]. Mucins are negatively charged macromolecules that attract or repel drugs through electrostatic interactions and protect the eye's surface from harmful external stimuli and pathogens [34]. At the same time, the non-specific binding of drugs to tear enzymes (such as lysozyme), mucin layers, and proteins (such as albumin) prevents drugs from reaching the cornea and anterior chamber [35].

In addition, tear turnover increases after topical insolation of drugs, resulting in rapid clearance of drug molecules through nasolacrimal drainage (within one to two minutes) [6, 36]. Meanwhile, due to the limited surface area of the eye, ~ 30 μL of the drug dropped into the eye is quickly expelled down the lacrimal passage until the tear fluid returns to the normal volume (7–9 μL) [37]. Approximately 60% of the drug is eliminated 2 min after treatment with topical eye drops. After 8 min, the drug is diluted to 0.1%, and after 15 to 25 min, almost all the active ingredients are removed from the corneal surface [38].

### Cornea

The healthy cornea is a clear, avascular tissue and the main barrier for foreign substances to enter the anterior chamber [39]. Structurally, it comprises five layers: the outer epithelium, Bowman's membrane, intermediate stroma, Descemet's membrane and endothelial layer [40]. The barriers preventing drug penetration into parenchyma are mainly epithelial, stromal and endothelial layers [41].

The corneal epithelium is characterized by tight junctions within the surface cell layer [37]. Due to its lipophilicity, it is an obvious obstacle, especially for hydrophilic compounds [42]. Besides, the existence of cytochrome P450 (drug-degrading enzymes) and drug efflux pumps in epithelial cells is another reason for low drug bioavailability [43–45]. In contrast, the highly hydrated matrix structure is a layered arrangement of collagen fibers immersed in the extracellular matrix, hindering the diffusion of lipophilic drugs [46]. The endothelial cells act as a leakage barrier to aqueous humor due to the presence of gap junctions [47]. These features make the cornea a primary barrier that obstructs drug delivery to the anterior segment of the eye [48].

### Conjunctival and scleral barriers

The alternative route of drug entry into the eye after topical instillation is the non-corneal route comprised of the conjunctiva and sclera [7, 49]. The conjunctiva, a mucous membrane formed by a vascularized epithelial group and an inner stromal layer, is located on the eyelid's posterior surface and in the cornea's outer region [50]. It forms and maintains the tear film and protects the ocular surface from environmental pathogens [51]. Besides, the conjunctiva has a surface area that is around 17 times bigger than that of the cornea, making it more permeable than the cornea and offering a superior pathway for the absorption of macromolecules and hydrophilic compounds [52, 53].

Nevertheless, the conjunctiva is highly vascularized. Rather of staying localized in the intraocular segment, medicines that penetrate the conjunctiva can be systematically absorbed from the conjunctival sac or nasal cavity and distributed throughout the body [16, 54]. This mechanism can lead to huge drug loss into the systemic circulation, reducing bioavailability within the ocular region [54]. To enhance drug efficacy, high concentrations of the drug and repeated instillations are usually necessary to achieve the desired therapeutic effect. However, this approach can negatively impact patient compliance and increase the likelihood of side effects [55].

After clearance from the conjunctiva, the drug travels through the sclera to the anterior segment (transscleral route). The sclera is the white part of the eye and appears as an opaque, hard sheath that wraps around the outer layer of the eyeball [56]. It has relatively high permeability and a larger surface area than the cornea. The scleral penetration is mainly determined by the size of the drug molecule instead of its lipophilicity [41]. Scleral thickness seems to be a critical factor in transscleral drug delivery [57]. The spread of the drug across the sclera occurs through the perivascular space and between the scleral fibrils, eventually reaching the choroid and the retina [58].

### The blood-aqueous barrier

The blood-aqueous barrier, consisting of the non-pigmented ciliary body of the iris vasculature and the epithelial tissue of the endothelial cells, is the main barrier in the anterior segment of the eye, which prevents the non-specific entry of various solutes in the intraocular environment [59]. The permeability of drugs across the BAB is determined by the osmotic pressure and physicochemical properties of drug molecules [60]. Lipophilic and small-molecule drugs can pass through the BAB and exit the anterior compartment more rapidly than hydrophilic and large-molecule drugs. For instance, pilocarpine was discovered to have a faster clearance rate than inulin [61]. It remains a challenge for ocular drug delivery due to its specialized tissue barriers that can hinder therapeutic efficacy.

### The blood-retinal barrier

The blood-retinal barrier comprises internal and external components and is the most important barrier in the posterior part of the eye [62]. The inner BRB is formed by tight junctions between retinal capillary endothelial cells, while the outer BRB is formed by close junctions between retinal pigment epithelial cells [63]. The BRB prevents water, plasma components and toxic substances from entering the retina [64]. At the same time, it may also limit the access of drug molecules to the intraocular environment [65]. Hence, BRB is necessary to keep the eye as a privileged place to maintain normal visual function [66].

## Ocular diseases

At present, more than 500 kinds of eye diseases are known, such as glaucoma, macular degeneration, diabetic retinopathy, dry eye disease (DED), etc. The prevalence of ocular diseases is steadily increasing due to changing eye usage patterns and the ageing population. These conditions profoundly impact individuals' health and quality of life, emphasizing the urgent need for effective interventions. Drug therapy undoubtedly plays a pivotal role in treating many ocular diseases.

### Glaucoma

Glaucoma, an eye disease characterized by progressive vision loss, is the second leading cause of blindness worldwide after cataracts [67]. It is estimated that the number of glaucoma patients will increase to 111.8 million by 2040 [68]. High intraocular pressure (IOP) is an essential feature of glaucoma [69]. Elevated intraocular pressure can induce the loss of corneal endothelial cells [70]. In addition, high intraocular pressure can also compress the retinal blood vessels, leading to the damage of retinal ganglion cells and optic nerve [71].

Although glaucoma is considered a multifactorial disease, current treatment mainly focuses on lowering intraocular pressure to slow or reduce subsequent visual loss [72]. Treatment usually begins with topical anti-glaucoma medications. However, the bioavailability of topical administration is below 5% due to high precorneal loss and low corneal penetration [37, 48, 73]. At the same time, frequent ocular administration decreases patient compliance [74]. Therefore, it is necessary to use nanotechnology to effectively deliver drugs, improve bioavailability and maintain the efficacy of anti-glaucoma drugs.

### Age-related macular degeneration

AMD is the third leading cause of severe irreversible vision loss globally, and the number of AMD patients worldwide is expected to increase to nearly 300 million by 2040 [75]. It is clinically divided into early AMD and late AMD. The clinical symptoms of early AMD include: medium size stone fruit and retinal pigment changes, and late AMD is classified as neovascular (also called wet or exudative) or non-neovascular (also called atrophic, dry or non-exudative), which may lead to central vision loss and legal blindness [76].

High doses of zinc and antioxidant vitamin supplements can slow disease progression from early to advanced stages [77]. Intravitreal injection (IVT) of anti-vascular endothelial growth factors (VEGF) (such as bevacizumab (Bev), aflibercept, etc.) effectively treats neovascular AMD, but it's still invasive [78]. Therefore, exploiting new drug delivery systems for personalized drug delivery is particularly important.

### Diabetic retinopathy

Diabetic retinopathy is a chronic complication of diabetes and the leading cause of vision loss and blindness globally [79]. In severe cases, retinal detachment can gradually manifest as blurred vision, ocular floaters, distorted vision, and even partial or complete vision loss [80].

Clinically, if laser treatment is performed in time, retinal circulation can be improved, avoiding vitreous hemorrhage and retinal neovascularization. However, for patients with macular oedema, it is usually necessary to inject anti-VEGF to treat macular oedema and improve vision [81]. Unfortunately, regular intravitreal injections may cause damage to the ocular tissue, and not all patients respond optimally [82, 83]. Vitrectomy is needed in case of fundus hemorrhage or proliferative vitreoretinopathy [84]. Given the low bioavailability of drugs, potential adverse effects, and inevitable risks in major surgery, novel drug delivery methods are required to bring new ideas for the therapy of DR.

### Dry eye disease

Dry eye disease, known as dry keratoconjunctivitis, is a multifactorial ocular surface disease [85]. It is characterized by tear film instability, hypertonicity, inflammation, ocular surface damage, and nerve paresthesia [86]. The global prevalence of dry eye is five to 50% [87]. The symptoms of DED include ocular irritation, pain, soreness, foreign body sensation, and decreased vision. DED seriously affects the quality of patients' lives, causes psychological anxiety, and adds a huge economic burden to society [88, 89]. To date, the pathogenesis of DED has not been fully elucidated, and most researches perceived that inflammation is the core of its pathogenesis [90].

The diagnosis and treatment of DED can be divided into two main categories: dehydration type and evaporation type [86]. Common drug treatments include artificial tears, local secretagogues, corticosteroids, and immunosuppressants; however, there are side effects such as ocular discomfort, low patient compliance, elevated intraocular pressure, and glaucoma [91]. Exploiting new drug delivery methods to overcome ocular barriers and improve drug bioavailability is particularly critical.

## Traditional routes of drug administration

**Fig. 3:**
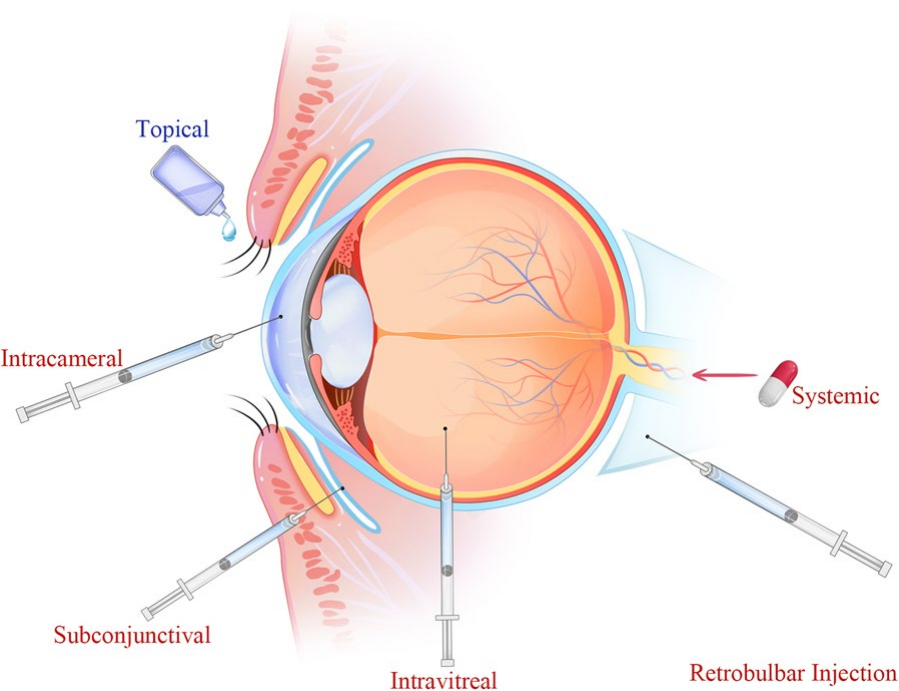
Routes of drug administration for ocular delivery. They mainly contain topical administration, subconjunctival and transscleral administration, intracameral administration, intravitreal injection and systemic administration et al.

The traditional routes of administration mainly include topical administration, conjunctival and scleral administration, intracameral administration, intravitreal injection, retrobulbar injection, systemic routes et al. [41]. The traditional routes of ocular drug administration are shown in Fig. 3. Depending on the routes of administration, one or more ocular barriers must be bypassed to allow the drug to reach the targeted site. Table 1 outlines several traditional routes of administration and their associated advantages and limitations.

**Table 1:** Comparison of various routes of ocular drug administration

Delivery routes	Advantages	Disadvantages	Diseases Treated
Topical	High patient compliance, self-administration, noninvasive nature	Corneal barrier, dilution and efflux, low bioavailability, high dosing	Conjunctivitis, keratitis, uveitis, episcleritis, scleritis, blepharitis
Subconjunctival and transscleral administration	Anterior and posterior drug delivery, ideal for depot formation	Choroidal and conjunctival circulation increase toxicity subconjunctival hemorrhage	Glaucoma, AMD cytomegalovirus retinitis,
Intracameral administration	Reduce corneal and systemic side effects. topical steroid use; high anterior chamber drug concentration	Toxic endothelial cell destruction syndrome and toxic anterior segment syndrome	Anesthesia, inflammation, endophthalmitis, pupil dilation
Intravitreal injection	Direct delivery to the vitreous humor and retina, BRB avoidance, high bioavailability and acute dosing	Poor patient compliance, invasiveness, drug toxicity, retinal detachment, cataract endophthalmitis, hemorrhage	AMD, central/branched retinal vein occlusion, diabetic macular edema, cytomegalovirus retinitis
Retrobulbar injection	Selective delivery to both anterior and posterior segments, avoidance of corneal and conjunctival barriers, long duration of action, a site for depot formulations	Poor patient compliance, invasiveness, drug deposition,compliances including pain, bleeding, infection, scarring, eyeball or optic nerve damage	Anesthesia
Systemic administration	High patient compliance	Blood ocular barriers, low bioavailability, systemic toxicity caused by high dosing	Scleritis, episcleritis, cytomegalovirus retinitis

### Topical administration

Topical administration is the most common and straightforward route of ocular drug administration [41]. Compared with systemic administration, it has the advantages of (1) being relatively non-invasive, (2) minimizing systemic side effects of the drug, and (3) the relative ease of patient administration [92, 93]. Therefore, ophthalmic solutions are the first choice for treating many eye diseases, such as infection, inflammation, DED, glaucoma, and allergy [94]. It is estimated that topical ophthalmic solutions account for 95% of the commercially available products in the global ophthalmic medicines market [95].

However, due to the unique physiological and anatomical structure of the eye, drug delivery in the eye is limited, and bioavailability is usually less than 5% [96]. High drug concentrations and repeated instillation are commonly needed to improve the efficacy of drug administration through the local route, which may lead to poor patient compliance and numerous side effects [6].

There are two main strategies to improve ocular bioavailability after topical administration: (a) increase the pre-corneal retention time, and (b) enhance the permeability of corneal, scleral, or conjunctival drugs [16]. Various approaches have been proposed to prolong drug residence time after topical administration, including prodrugs, mucus osmotic particles, enhancers, collagen corneal shields, and therapeutic contact lenses [97]. In addition, nanocarriers also open up new windows for liquid and semi-solid formulations to increase drug availability [48].

### Subconjunctival and transscleral administration

Subconjunctival administration is a minimally invasive and effective route to deliver drugs to the anterior or posterior eye chamber, avoiding the corneal and blood-aqueous barriers, potential adverse effects and first-pass metabolism of some systemic agents [98, 99]. However, the subconjunctival route may result in drug loss due to blood and lymphatic drainage through the conjunctiva [55, 100].

Similarly, transscleral administration is a simple, minimally invasive, and more suitable method for patients. This route can bypass the obstacles in the anterior part of the eye [101]. At the same time, the large surface area of the sclera (about 95% of the total surface area of the eye) offers the chance of delivering antioxidants, neuroprotective agents or anti-angiogenic agents to targeted sites in the retina [102]. It has been demonstrated that molecules up to 70 kDa can easily penetrate the sclera, whereas molecules that cross the cornea are under 1 kDa [99]. However, due to the dynamic barriers, the intraocular bioavailability of this method is lower than that of the direct intravitreal injection route [41, 103].

### Intracameral administration

Intracameral administration injects drugs directly into the eye's anterior chamber [104]. This local delivery approach avoids the adverse effects and first-pass metabolism with some systemic agents. At the same time, it also avoids the cornea, conjunctiva, and BAB [105]. Thus, intracameral injections allow relatively easy and efficient drug delivery to the anterior segment of the eye [106, 107]. Currently, intracameral injections are used for prophylactic antibiotics or anesthetics associated with eye surgeries [108–111].

However, administration in the anterior chamber can't deliver drugs to the posterior chamber of the eye. At the same time, drugs in the anterior chamber usually require reorganization, dilution, sterility, special preparations without preservatives, and appropriate concentrations and doses [112]. Corneal endothelial cell toxicity and toxic anterior segment syndrome may occur if incorrect doses and preparations are used [113].

### Intravitreal injection

Intravitreal injection is a preferred method of medicine administration in the posterior part of the eye to treat ophthalmic diseases in the eyeball [114]. Due to vitreous fluid turnover, free drugs can be removed quickly after IVT injections [3]. Frequent IVTs are required to achieve good therapeutic results, which may result in side effects such as retinal detachment, eyeball infection, endophthalmitis and elevated intraocular pressure [115, 116]. Therefore, the optimal protocol for IVT is a one-time injection of the drug without retracting the needle and keeping the eyeball system closed.

Recent studies have focused on maintaining therapeutic effects, prolonging treatment intervals and protecting normal ocular tissues. NPs, intravitreal implants, hydrogels, combinatorial systems, and minimally invasive techniques are under preclinical and clinical investigations, which act as safer and more efficient alternatives to combat ophthalmic diseases [24, 117].

### Retrobulbar injection

The retrobulbar route involves injecting needles through the eyelid and orbital fascia to deliver drugs to the retrobulbar space [118, 119]. Retrobulbar injection of triamcinolone acetonide treats macular oedema caused by retinal vein occlusion [120]. The antifungal effect of retrobulbar injection of amphotericin B is higher than intravenous injection [121]. Retrobulbar injection of chlorpromazine is used to treat painful blind eyes [122].

### Systemic administration

Systemic administration (including parenteral and oral dosing) is an alternative method of drug delivery. At present, systemic administration has been used to deliver antibodies, antibiotics, and carbonic anhydrase inhibitors to treat diseases such as endophthalmitis, elevated intraocular pressure, and uveitis [123–126]. Nevertheless, due to the ocular barriers and the tight junctions of the retinal pigment epithelium that allow only one to two per cent of the drug to reach the retinal and vitreous regions, frequent administrations are required to obtain the desired therapeutic effect, which may contribute to systemic side effects and poor patient compliance [108, 127]. Therefore, it is not an ideal mode of administration.

## Pharmacokinetics

**Fig. 4:**
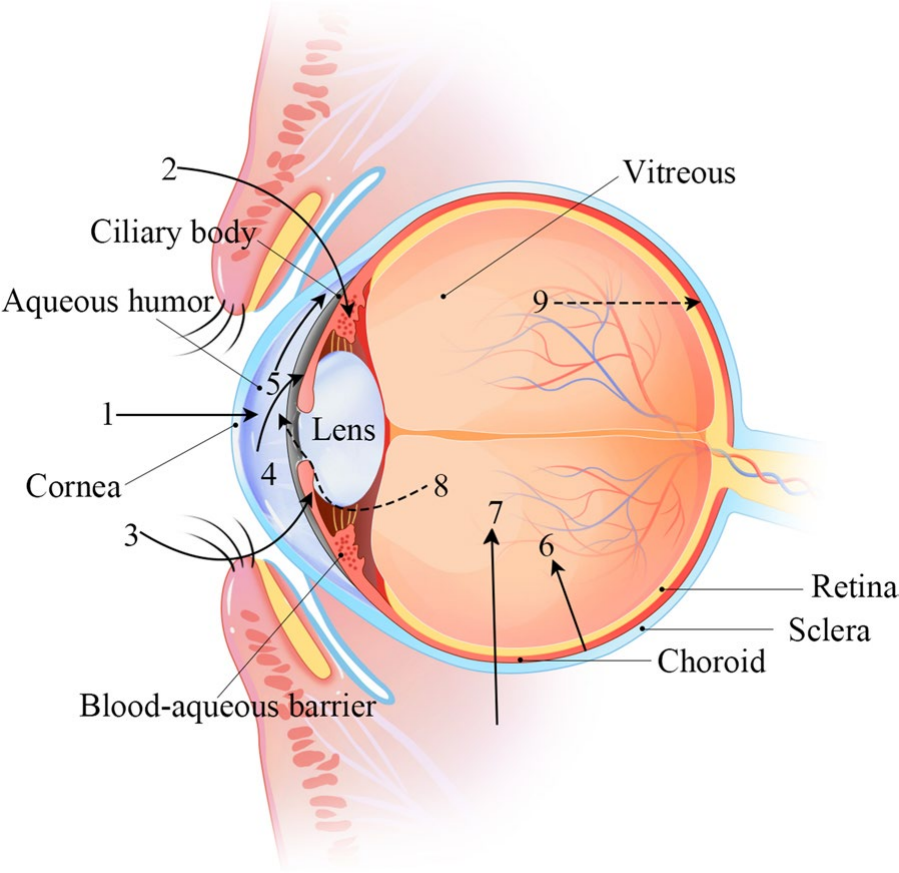
The pathways of drug metabolism. According to the arrows in the figure, there are nine major pathways of drug metabolism, as described in detail above

Based on the ocular barriers and drug administration described above, ocular pharmacokinetics, including penetration and elimination, are discussed in detail. As shown in Fig. 4 [6, 128], it mainly contains the following pathways: (1) through the tears and cornea into the anterior chamber, (2) non-corneal permeation into the anterior uvea through the conjunctiva and sclera, (3) drug from the bloodstream cross BAB to the anterior chamber, (4) drug from the aqueous humor cross BAB to the systemic circulation, (5) drug elimination from the aqueous humor to the trabecular meshwork and Schlemm's canal, (6) drug distribution from the circulation through BRB to the posterior segment of the eye, (7) intravitreal administration, (8) elimination from the vitreous body into the posterior compartment via an anterior route, and (9) elimination from the vitreous body via a posterior route through BRB.

## Nanotechnology-based ocular drug delivery systems

**Fig. 5:**
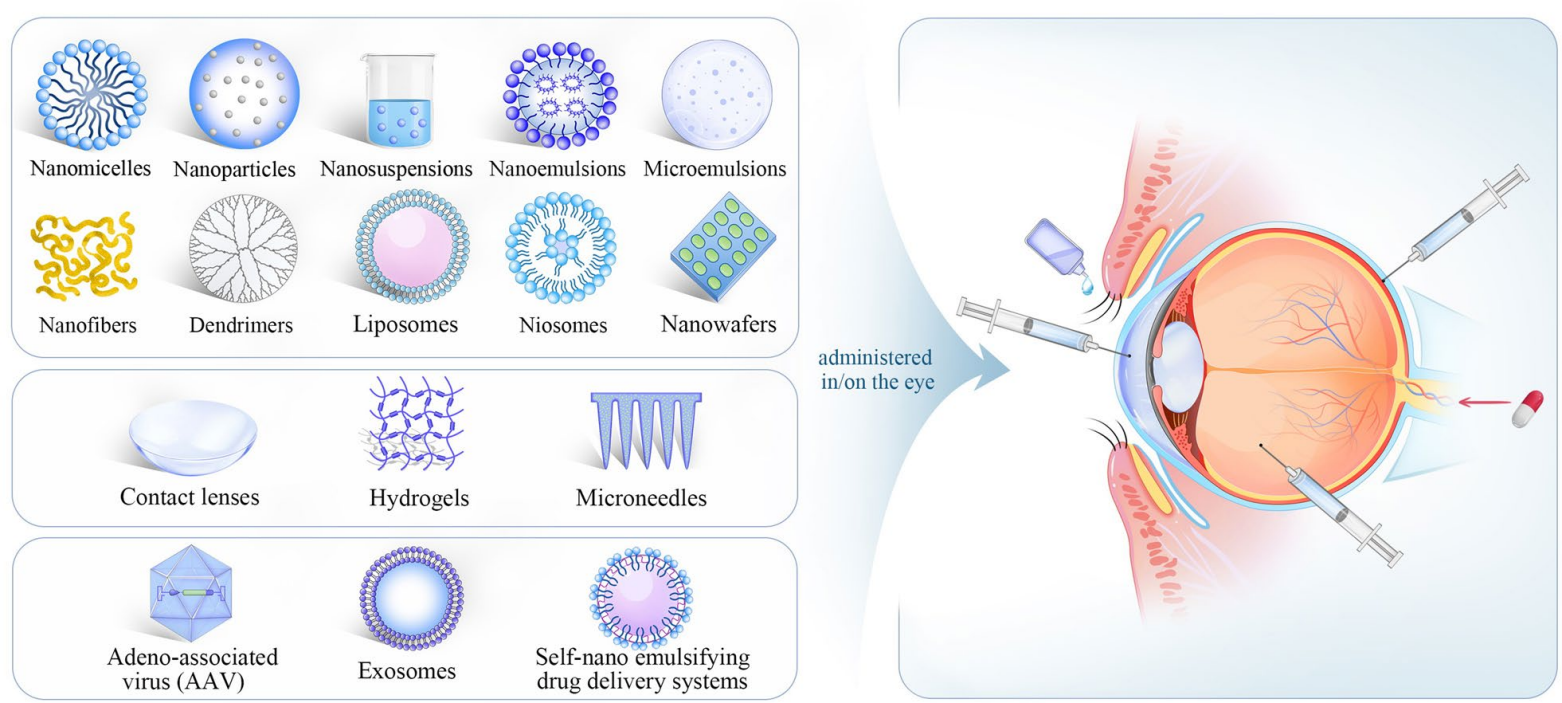
Nanotechnology based drug delivery systems for ocular application

To overcome ocular drug delivery barriers and improve drug bioavailability, novel drug delivery systems have been developed. Nanocarriers' development offers many advantages, including overcoming ocular barriers, promoting transcorneal permeability, prolonging drug residence time, reducing the dosing frequency, improving patient compliance, reducing drug degradation, achieving sustained/controlled release, drug targeting and gene delivery [23]. Many ocular drug delivery systems such as nanomicelles, NPs, nanosuspensions, NEs, microemulsions, nanofibers, dendrimers, liposomes, niosomes, nanowafers, MNs and exosomes (Fig. 5), have shown splendid delivery potential in both vitro and vivo studies, enhancing drug permeability across the ocular barriers and prolonging the residence time in the eye [23, 129].

### Nanomicelles

Nanomicelles are core–shell nanocarriers formed by spontaneous assembly of amphiphilic copolymers with hydrophobic groups as the core and hydrophilic groups as the outer shell [130]. Usually, the particle size ranges from 10 to 100 nm and can be divided into three categories: polymers, surfactants, and multi-ion composite nanomicelles [131]. Besides, hydrophobic interactions, hydrogen bonds, electrostatic interactions, etc., are the driving forces for polymer micelle formation [132]. Positive micelles are generally formed when the hydrophobic moiety forms clusters within the core and the hydrophilic moiety is aligned outwards to increase contact with water. Likewise, when the opposite arrangement occurs, the aggregates are referred to as reverse micelles [133]. Positive micelles are used to encapsulate, solubilize, and deliver hydrophobic drugs, whereas reverse nanomicelles are used to encapsulate and deliver hydrophilic drugs [134]. The unique chemical structure of nanomicelles can solubilize drugs internally, reduce adverse reactions, improve the stability of drugs, and have a sustained release effect, regarded as safe alternatives for ocular drug delivery [135, 136].

Cyclosporine is an immunomodulatory drug employed in treating DED. Given its relatively high molecular weight and poor permeability, Ghezzi et al. prepared micelles using tocopherol polyethene glycol 1000 succinate (TPGS) and Solutol®HS15 for cyclosporine delivery. Meanwhile, the addition of α-linolenic acid was evaluated based on the results of using fatty acids for micelle preparation [137, 138] and drug loading [139, 140]. Also, the effect of TPGS as a corneal permeability promoter and irreversible changes in tissue permeability were analyzed. It was demonstrated that TPGS micelles (approximately 13 nm in size), loaded with 5 mg/mL cyclosporine, facilitated drug retention in the cornea and sclera and possessed good tolerance for ocular applications [141].

Besides, XU et al. developed chitosan oligosaccharide-valylvaline-stearic acid (CSO-VV-SA) nanomicelles and hydrogen-castor oil 40/octyl alcohol 40 (HCO-40/OC-40) hybrid nanomicelles for topical ocular drug delivery. Neither nanomicelles produced significant cytotoxicity in human corneal or conjunctival epithelial cells. Dexamethasone in both nanomicelles was detectable in rabbit tears for over 3 h. Notably, the delivery efficiency of CSO-VV-SA nanomicelles was not inferior to HCO-40/OC-40 hybrid nanomicelles at both cellular and animal levels, which suggested that CSO-VV-SA nanomicelles would have further potential for clinical translation as novel drug delivery carriers [142].

Traditional intravitreal injection of anti-VEGF into the posterior part of the eye to treat retinal diseases is invasive and accompanied by various complications. A nano-micelle drug delivery system composed of polyethene glycol (PEG), polypropylene glycol, and polycaprolactone (PCL) fragments was developed to avoid these. The copolymer EPC (nEPC) locally delivers aflibercept to the posterior segment of the eye via the corneal-scleral routes. Animal experiments have shown that aflibercept-loaded nEPCs (nEPCs + A) can penetrate the cornea in an ex vivo porcine eye model and deliver aflibercept to the retina to promote choroidal neovascularization (CNV) regression in a mouse model of laser-induced CNV. Besides, nEPCs + A showed good biocompatibility and intrinsic anti-angiogenic properties. These findings suggest that nEPCs may be promising candidates for further clinical applications [143].

### NPs

NPs are colloidal drug carriers with ideal sizes ranging from 10 to 100 nm [21]. They are mainly divided into polymer and lipid NPs [144]. NPs used in ocular preparations are composed of lipids, proteins, and natural or synthetic polymers such as albumin, sodium alginate, chitosan, polylactide-coglycolide (PLGA), polylactic acid (PLA), and PCL [145]. Besides, the surface charge of NPs highly affects their effective ocular absorption. Since corneal and conjunctival tissues have negatively charged surfaces, cationic NPs have a higher retention time on the ocular surface than anionic NPs [146].

To date, NPs have been used widely to deliver drugs to the targeted tissue in the eye, with the advantages of: (1) smaller and less irritating; (2) providing sustained drug release to avoid repeated dosing; (3) preventing non-specific uptake or premature degradation; (4) providing better absorption and improving intracellular penetration; and (5) targeted delivery to desired tissues [42, 147–149].

As a synthetic polymer, PLGA has been widely used to prepare NPs for ocular drug release due to its biodegradability, excellent biocompatibility, and capacity to modulate drug release by altering molecular weight, terminal groups, and the lactide-to-glycoside ratio [150, 151]. The US Food and Drug Administration (FDA) has approved various drug delivery products with PLGA.

In one study, chitosan-coated polylactide-glycolic acid NPs (CS-PLGA NPs) were developed to deliver Bev (an anti-VEGF drug used widely for treating DR) to the posterior chamber of the eye. The confocal laser scanning microscopy and pharmacokinetics showed that CS-PLGA NPs had better permeability than the traditional drug solution, with higher concentrations of Bev (above 22 ng/mL for 6 weeks) in the posterior ocular tissues. In the retinopathy model, subconjunctival injection of CS-PLGA NPs significantly reduced the level of VEGF in the retina for 12 weeks compared with local and intravitreal injections. Thus, CS-PLGA NPs can potentially be used to target the retina for drug delivery [152].

Kim et al. delivered NPs loaded with the drug latanoprost into the eye by iontophoretic method to treat glaucoma. These NPs were made of PLGA and had the advantages of releasing the latanoprost sustainably and prolonging the drug residence time. The 300 nm NPs showed the most durable drug effect in vivo. It lasted more than 7 days and increased its efficacy by approximately 23-fold compared to Xalatan^®^ (a commercially available latanoprost eye drop), which offers a new strategy for prolonging the efficacy of drugs and reducing the frequency of drug administration in the treatment of glaucoma [153].

**Fig. 6:**
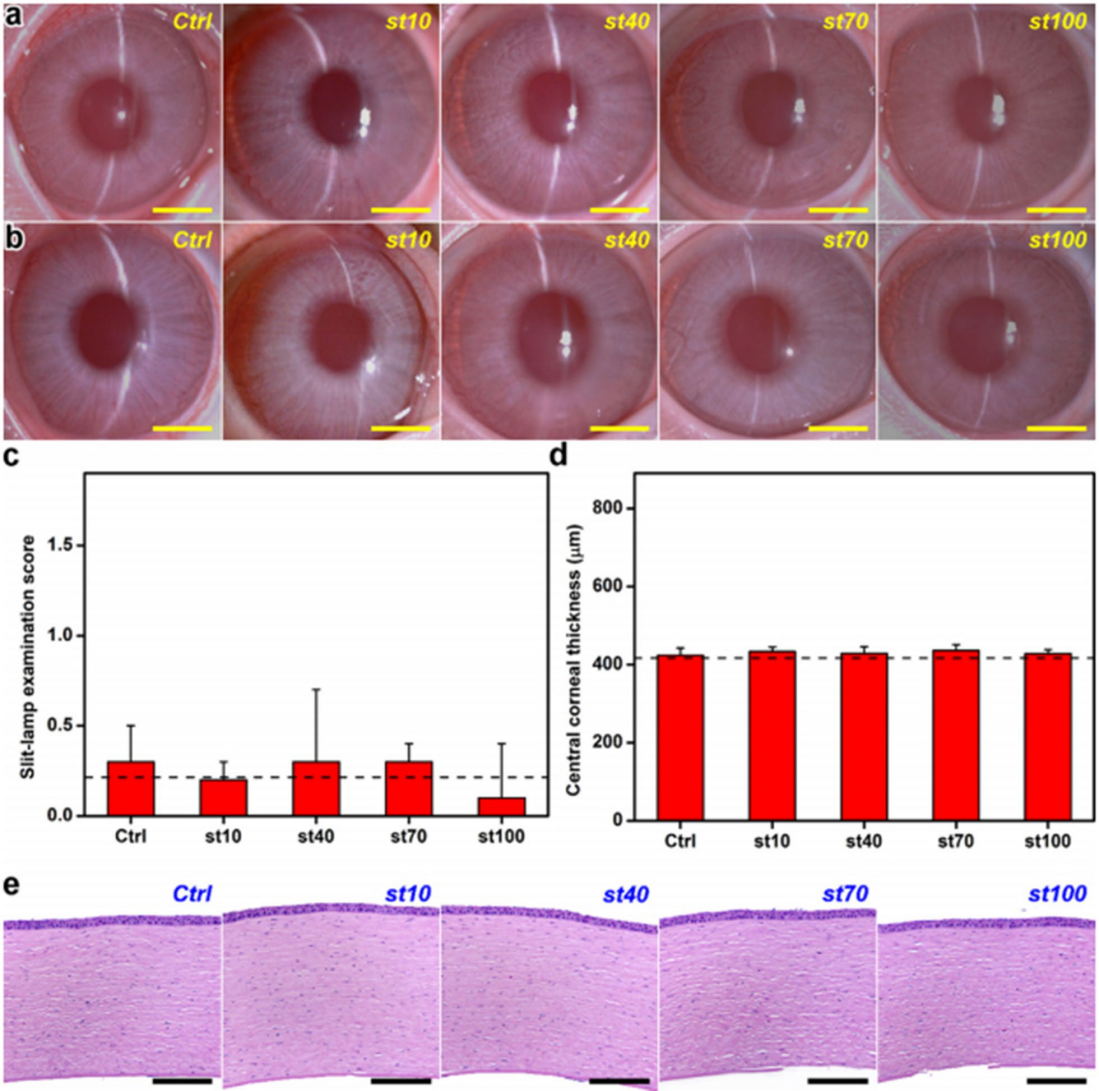
The representative images of rabbit eyes taken with a slit-lamp biomicroscope after intracameral administration of pilocarpine-loaded HPLA NP (st10, st40, st70, and st100) dispersions or BSS buffer (Ctrl group) at 0 (**a**) and 56 (**b**) days. **c** The scores of slit-lamp examinations at 56 days **d** Central corneal thickness at 56 days. **e** The histology of corneal tissues at 56 days postoperatively

Likewise, Nguyen et al. developed hollow polylactic acid NPs and innovatively investigated the role of shell thickness in developing long-acting drug carriers to treat glaucoma effectively. Among the four NPs with an adjustable shell thickness of 10 to 100 nm (~ 10, 40, 70, and 100 nm), a medium-thickness shell (~ 40 nm) manifested the most effective release curve of pilocarpine and sustained relief of high IOP for more than 56 days in the rabbit glaucoma model, which may protect the structural integrity of the corneal endothelium, as well as attenuate retinal and optic nerve degeneration (Fig. 6). Thus, this finding implies the potential of the shell thickness effect in developing long-acting drug delivery systems that can be used to treat some chronic eye disorders [154].

In contrast to polymeric NPs, lipidic formulations are known to be less stable for sustained drug release. Recently, adding polymers to lipidic NPs formulations has gained wide interest in increasing the stability of nanocarriers [16]. Schnichels et al. investigated lipid DNA NPs functionalized for the loading of brimonidine through specific aptamers and via hydrophobic interactions with double-stranded micelles. Both NP types significantly reduced IOP in living animals. Overall, IOP reduction was observed in 74% (SEM: ± 3%) and 54% (SEM: ± 1%) of the number of animals treated with two types of DNA NPs once daily for 5 weeks, compared to the animals treated with the original brimonidine(36%, SEM: ± 3%). Importantly, NPs loaded with brimonidine showed no toxicity and improved efficacy. In conclusion, these drug delivery systems offer great opportunities to treat glaucoma [155].

To improve the biocompatibility of the NPs, it is worth noting that the combination of biomimetic technology and NPs has brought new ideas for non-invasive drug delivery to the eye. Chen et al. reported adhesive and therapeutic biomimetic nanocoatings on ocular surfaces using sebocyte membranes with integrin-β1 overexpressed to coat NPs. The NPs specifically bind to the Arg-Gly-Asp sequence of fibronectin in the ocular epithelium, which is critical in supplementing the lipid layer, stabilizing the tear film and prolonging the retention time for 24 h. In mouse and rabbit DED models, dexamethasone-loaded nanocoatings effectively reduced corneal opacity and inflammatory cytokine levels, improved corneal epithelial recovery and restored tear secretion. This study provides new insights to protect the ocular surface and prolong the retention time of the drug [156].

**Fig. 7:**
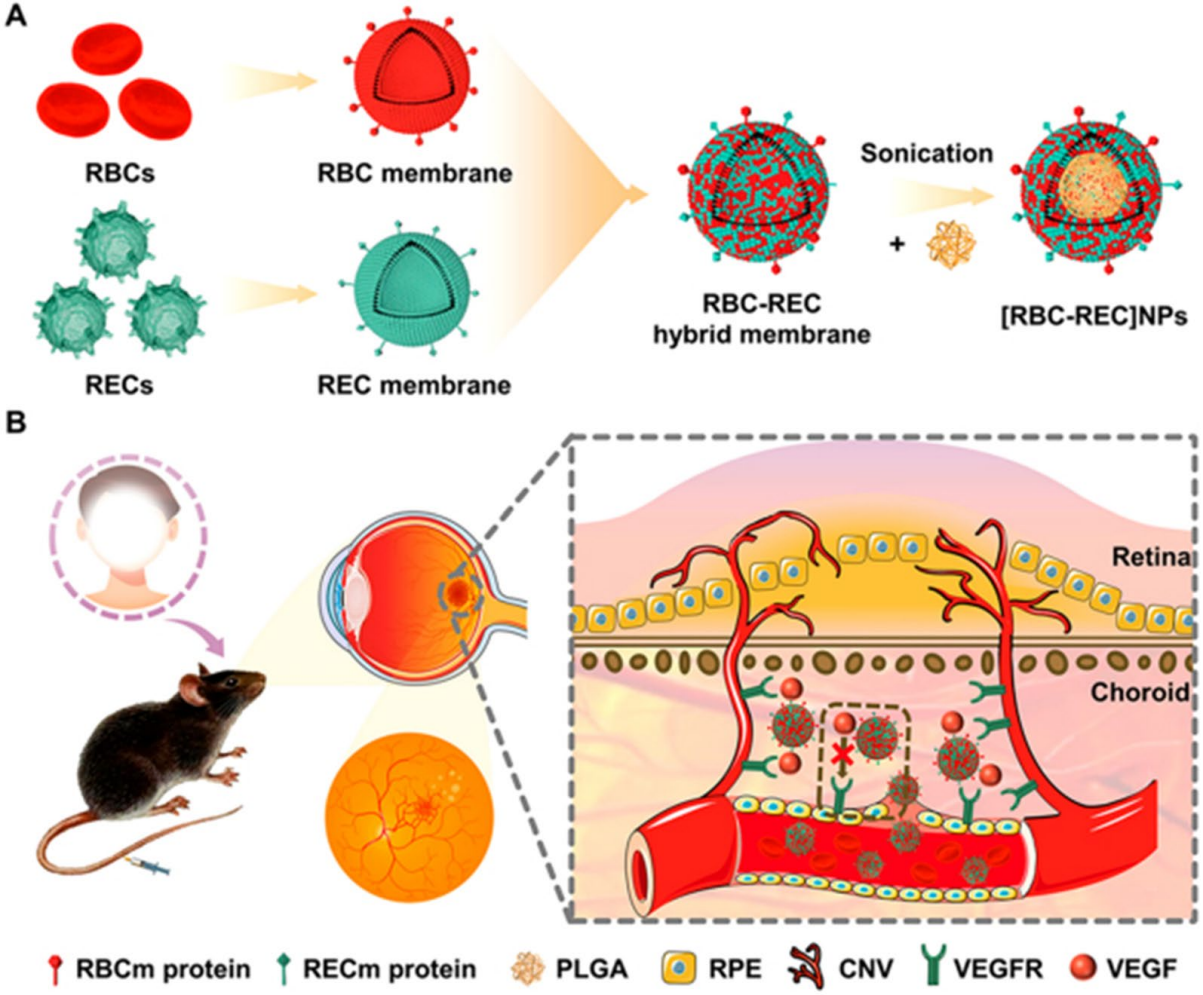
The schematic illustration of hybrid cell-membrane-cloaked biomimetic nanoparticles taking advantage of the targeting property of REC and the immune evasion capability of RBC for the therapy of laser-induced CNV. **A** The process of preparing hybrid cell-membrane-coated NPs. **B** Intravenous administration of NPs absorbs proangiogenic factors, leading to the blocking of their influences on the endothelial cells of the host neovascularization

Similarly, Li et al. developed an alternative anti-angiogenic agent based on hybrid cell-membrane-coated NPs for the non-invasive treatment of choroidal neovascularization (Fig. 7). The fusion of erythrocyte membrane protected the mixed membrane-coated NPs from phagocytosis by macrophages. The retinal endothelial cell membrane coating provides isotype targeting and binding ability to VEGF. In laser-induced CNV mouse models, intravenous injection of the NPs effectively inhibited ocular angiogenesis. The inhibition rates of migration and invasion were ~ 77.5% and ~ 78.5%, respectively. At the same time, excellent treatment results were achieved in reducing the leakage and area of CNV, analyzed by fluorescein angiography and indocyanine green angiography. In conclusion, biomimetic anti-angiogenic nano agents open a new window for the non-invasive treatment of CNV [157].

Although NPs show promise for treatment of ophthalmic diseases, there are still significant constraints that prevent them from being widely used in clinical practice. These limitations include inadequate drug loading, premature drug release during storage, difficulty in achieving homogeneous particle dispersion, and toxic effects related to the concentration of the surfactants [22]. More studies should be conducted to promote the clinical translation of NPs.

### Nanosuspensions

The nanosuspension consists only of submicron colloidal dispersions of drug nanocrystals. Surrounded by stabilizers, it is one of the most promising approaches for delivering poorly soluble active ingredients [158, 159]. Unlike conventional matrix-framed nano-systems, nanosuspension does not require a carrier material. It contains 100% pure drug NPs in the nanometer range and is usually stabilized by surfactants or polymers [160]. They have the advantages of increased residence time, sustained drug release, and enhanced drug solubility [161].

To improve the bioavailability of moxifloxacin hydrochloride, Josyula et al. used an ion-pairing method to fabricate an insoluble moxifloxacin–pamoate (MOX-PAM) complex, which was further formulated as a mucus-penetrating nanosuspension eye drops (MOX-PAM NS). Compared with Vigamox^®^ (commercial formulation) in healthy rats, MOX-PAM NS significantly increased ocular drug absorption with about 1.6-fold greater C_max_ and had better antibacterial effects. Treatment with MOX-PAM NS administered once daily was similar to that with Vigamox^®^ administered three times daily in a rat model of ocular Staphylococcus aureus infection. These results demonstrated nanosuspension's high translational and clinical relevance [162]. Moreover, nanosuspensions have been used as a platform for ocular delivery of immunosuppressive agents [163, 164].

Furthermore, nanosuspensions can also be combined with other nanotechnology. Triamcinolone acetonide (TA) is a synthetic corticosteroid widely used to treat several inflammatory conditions. One study developed a hybrid nanosuspension and dissolving MNs system for effective and minimally invasive transscleral delivery of the hydrophobic drug TA. After optimization, TA NS was incorporated into the MN array by high-speed centrifugation to form a bilayer structure. TA NS-loaded MNs were strong enough to penetrate the excised porcine sclera, with an insertion depth greater than 80% of the needle height, and dissolved rapidly (< 3 min). Notably, the transscleral deposition study showed that the amount of TA deposited in the sclera after 5 min application of NS-loaded MN was 56.46 ± 7.76 μg/mm^2^, which was 4.5-fold higher than that of common drug-loaded MN (12.56 ± 2.59 μg/mm.^2^) [165].

Despite these encouraging nanosuspension results, the stability issues related to nanosuspensions remain unresolved. The stability properties of electrostatic and steric stabilizers, the maximum achievable particle size and physical stability are key factors that need further study [166].

### Nanoemulsions

Nanoemulsion is a transparent or translucent, thermodynamically unstable but kinetically stable system with sizes ranging from 20 to 500 nm [167, 168]. According to the classification of the dispersed phase system, NEs are mainly divided into (1) water-in-oil (w/o) NEs: continuous phase-containing dispersion of water droplets, (2) oil-in-water (o/w) NEs: continuous phase-containing dispersion of oil droplets, and (3) bi-continuous NEs: oil microdomains and water intermingled in the system, and various NEs modifications [169].

Based on nanotechnology, NEs are widely used as non-invasive, cost-effective drug delivery vehicles and can be easily scaled up for commercial production. Besides, compared with traditional drug delivery methods, NEs have the advantages of prolonged anterior corneal retention time, sustained drug release, high penetration ability, enhanced ocular bioavailability, and easy sterilization improvement [170–173]. At the same time, it can also be used to treat different eye diseases, such as DED [174], fungal keratitis [175], herpes simplex keratitis infection [176], glaucoma [177], etc.

Dukovski et al. developed a functional cationic ophthalmic NE with 0.05% (w/w) chitosan and nonsteroidal anti-inflammatory drugs loaded, using chitosan as the cationic and lecithin as the anionic surfactant. In an ex vivo porcine cornea model, NPs extended the drug retention time on the ocular surface, stabilized the tear film and acted on inflammatory components, providing a possibility for the therapy of DED [174].

Bacterial keratitis is a serious eye infection which can result in severe visual disability. Youssef et al. prepared a ciprofloxacin-loaded nanoemulsion (CIP-NE) using oleic acid and Labrafac^®^ lipophilic WL 1349 as the oil phase and Tween^®^80 and Poloxamer 188 as surfactants. Optimized nanoemulsion was spherical in shape and showed a globule size, zeta potential, and polydispersity index of 121.6 ± 1.5 nm, −35.1 ± 2.1 mV, and 0.13 ± 0.01, respectively, with 100.1 ± 2.0% drug content. The in vitro release and ex vivo trans-corneal permeation studies showed sustained release and 2.1-fold enhanced penetration compared with commercial ciprofloxacin, suggesting that the CIP-NE formulation might be used as a promising nanocarrier to enhance the therapeutic efficacy of bacterial keratitis [178].

Travoprost is a synthetic prostaglandin F2α analogue used in the therapy of glaucoma. Given its water insolubility and oiliness, new delivery systems must be proposed to improve its bioavailability and maintain its release. Ismail et al. used the travoprost nanoemulsion as a novel carrier, exhibiting suitable nanodroplet size, zeta potential, refractive index, pH, controlled release, and adequate stability under accelerated conditions. Compared with Travatan^®^ eye drops, travoprost nanoemulsion has a short-term safety profile, improved bioavailability, and sustained IOP reduction for 60 h. Therefore, travoprost nanoemulsion is a good ocular delivery vehicle for the therapy of glaucoma [179].

Although NEs can be used in ocular preparations, NEs still have some drawbacks, such as eye irritation and low viscosity. In addition, NEs are thermodynamically unstable and may decompose over time through various physicochemical mechanisms, such as gravitational separation, flocculation, Oswald maturation, and coalescence [22]. Future studies should focus on physicochemical analysis, toxicity analysis in vivo and in vitro tests, and optimization of some formulation development parameters, further promoting the transformation of NE-based drug delivery to clinical application.

### Microemulsions

Microemulsions have colloidal dispersions composed of specific proportions with different phases, including aqueous phase, oil phase, cosurfactant, and surfactant. Their droplet sizes range from 10 to 100 nm [180]. Based on the types and amount of surfactant in the formulation, microemulsions can be divided into three categories: o/w, w/, and bi-continuous structures [181]. Typically, o/w microemulsion has a higher water comparison, while w/o microemulsion has a higher oil comparison. Microemulsions have been extensively explored as a drug delivery vehicle for ocular preparations to overcome various obstacles and reduce the frequency of daily eye drops [182].

Microemulsions are the most potential submicron drug carriers, especially for poorly water-soluble drugs. At the same time, microemulsions are thermodynamically stable, inexpensive and relatively simple to produce [183]. Various researches have demonstrated the efficiency of microemulsions in delivering multiple drugs to different issues of the eye.

For instance, Mahran et al. used oleic acid, Cremophor EL, and propylene glycol to prepare microemulsion preparations loaded with TA for treating uveitis. Different pseudo-ternary phase diagrams were also constructed using the water titration method, and the formulation composed of oil, surfactant-co-surfactant (1:1), and water (15:35:50%w/w, respectively) turned out to be most effective (complete drug release within 24 h). In a uveitis-induced rabbit model, the developed TA-loaded microemulsion observably reduced inflammation signs, protein content, and inflammatory cells compared to commercially available suspensions [184].

Besides, Santonocito et al. used a novel microemulsion system (NaMESys) to deliver sorafenib to the retina. It has shown that NaMESys carrying 0.3% sorafenib (NaMESys-SOR) has good cytocompatibility and tolerability. It can also reduce pro-inflammatory and proangiogenic mediators in a robust model of proliferative retinopathy. Furthermore, NaMESys-SOR significantly inhibited the mRNA expression of tumor necrosis factor-alpha (20.7%) and inducible nitric oxide synthase (87.3%) in retinal ischemia–reperfusion rats compared with the control group. In addition, NaMESys-SOR also observably inhibited 54% of the neovascularization lesions in mice with laser-induced CNV. The findings show that NaMESys eye drops may effectively deliver various drugs to the retina [185].

Interestingly, some researchers have found that the methylglyoxal (MGO) concentration in Manuka honey is quite high and can effectively manage bacterial overload. Based on these, D. Rupenthal et al. prepared liquid crystal microemulsions containing alpha-cyclodextrin-complexed Manuka honey and evaluated their antimicrobial function at relatively low MGO concentrations. The results showed that 100 mg/kg MGO formulation had significantly higher antibacterial activity against Staphylococcus aureus (especially at a density of 1 × 106 CFU/mL) in vitro than each of its individual components. Importantly, no corneal or conjunctival irritation was observed at concentrations consistent with accidental exposure to the ocular surface, which may provide new ideas for treating blepharitis [186].

In conclusion, these findings are worth further investigating the other therapeutic potential of the microemulsion, facilitating the continued exploration of novel drug delivery technologies.

### Nanofibers

Nanofibers are 1–100 nm diameter fibers [187]. Various natural polymers (such as chitosan, fibronectin, gelatin, collagen, silk, and ethyl cellulose) or synthetic polymers (such as PLA, PLGA and PCL) or combinations thereof can be used to produce nanofibers through the electrospinning process [188].

Nanofibers have the advantages of a high surface-to-volume ratio, high porosity, adjustable mechanical properties, strong drug-loading capacity, high encapsulation efficiency, and simultaneous delivery of multiple therapeutic agents [189]. In addition, nanofibers can help drugs cross physiological barriers and target tissues, providing long-term controlled drug release while minimizing drug distribution in other parts of the body [190]. These properties make it a unique candidate for drug delivery applications, diagnosis and treatment of various diseases, especially chronic eye diseases that need frequent administration [191, 192].

MEL exerts neuroprotective effects on retinal damage and neuronal damage associated with several chronic and degenerative eye diseases, such as AMD, DR, and glaucoma [193, 194]. Unfortunately, the short half-life and low bioavailability of MEL plasma (3–15%) limit the therapeutic effect [195, 196]. Romeo et al. used electrospinning to prepare polyvinyl alcohol (PVA) and PLA nanofibers. Both nano-systems were loaded with various concentrations of MEL (0.1, 0.3 and 0.5% w/w). PVA nanofibers release MEL quickly (within 20 min) and completely, whereas PLA nanofibers provide a slow and controlled release of MEL. Interestingly, the addition of Tween®80 provides faster dissolution and approximately a 20-fold increase in expansion properties. Based on the obtained results, the formulated MEL-supported nanofibers may be a promising carrier with improved biopharmaceutical properties for the ocular delivery of MEL [197].

Furthermore, nanofibers can be loaded with multiple drugs. Rohde et al. developed electrospun polymer fibers with gentamicin and dexamethasone, which are used to treat bacterial conjunctivitis. Upon contact with the ocular surface, the nanofibers are immediately dissolved in the tear fluid, quantitatively releasing the two active substances. The recovery rate was over 92% by fluorescence and quantitative chromatographic methods. In the pig microfluidic corneal model, the eye retention time was significantly longer than that of traditional eye drops. After 20 min of eye drops, the availability of drugs on the ocular surface increased by 342%. Notably, the polymer has good biocompatibility and sufficient storage stability for antibacterial activity within 12 weeks [198].

Similarly, Tawfik and his partners developed coaxial PLGA and polyvinylpyrrolidone nanofibers loaded with the antibiotic moxifloxacin hydrochloric acid and the anti-scarring agent pirfenidone for the treatment of corneal abrasion. Pirfenidone was fully released from the outer layer of PLGA after 24 h, and about 70% of moxifloxacin hydrochloride was released from the inner layer of polyvinylpyrrolidone within the same time. In addition, a single dose of fiber was as effective in inhibiting infection as four doses of moxifloxacin hydrochloride, supporting the potential of dual drug-loaded nanofiber systems as once-daily eye implants for treating corneal abrasion [199].

**Fig. 8:**
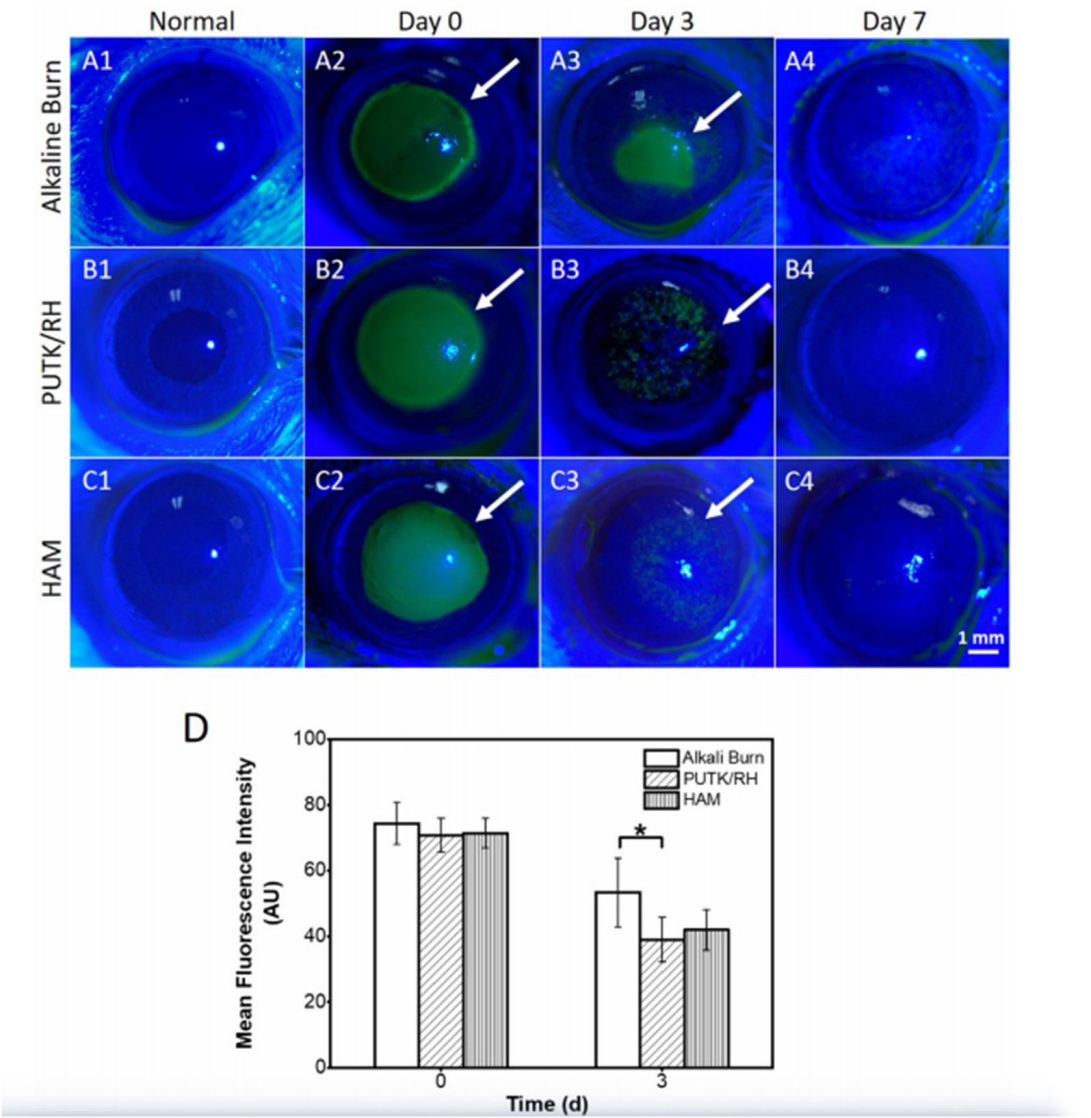
**A**–**C** The fluorescein-staining photographs of rat corneas transplanted with HAM and PUTK/RH patch after alkali burn. **D** Mean fluorescence intensity. The corneal epithelial defects (green region) are marked by white arrows point to. n = 5,*P< 0.05

Because of the nanofiber extracellular matrix-like structure, its production method is less costly and simpler than many nanostructured drug delivery systems [200–202]. In addition, nanofibers can be combined with other technologies. One study combined nanofibers with hydrogels for intravitreal anti-VEGF drug delivery. This modulated, injectable, biodegradable hydrogel nanofiber system can change the peptide concentration to adjust the dose, providing a broad application prospect for treating wet age-related macular degeneration [203]. Likewise, a double network patch was designed by compounding electrospinning nanofibers of thioketal-containing polyurethane (PUTK) with a reactive oxygen species (ROS)-scavenging hydrogel (RH) fabricated by cross-linking poly with thioketal diamine and 3,3’-dithiobis. The PUTK/RH patch has good transparency, high tensile strength, hydrophilicity and strong antioxidant activity. In a rat corneal alkali burn model (Fig. 8), the corneal fluorescein staining showed that the mean fluorescence intensity in PUTK/RH group decreased to 39.0 ± 6.7 AU, compared to the alkaline burn group (53.4 ± 10.5 AU) on day 3. Furthermore, PUTK/RH patch can accelerate corneal wound healing by inhibiting inflammation, promoting epithelial regeneration and reducing scar formation, which may be a new therapeutic strategy for the alkali burned cornea [204].

Besides, a visual device was developed using commercial contact lenses as substrate, metal-coated nanofiber mesh as conductor, and in-situ electrochemical deposition of poly (3, 4-ethylenedioxythiophene)/poly (styrene sulfonate) as adhesive material. This hydrogel contact lens has high permeability, excellent wettability, optical transparency and mechanical compliance. A study involving rabbit eyes demonstrated the safety of wearing this contact lens continuously for 12 h; no notable corneal wear or irritation was observed. This finding highlights the lens's high level of safety and its potential to serve as a versatile platform for eye health monitoring and drug administration [205].

### Dendrimers

Dendrimers are nano-sized (usually 2–100 nm), symmetric, hyperbranched and typically tree-shaped or star-shaped structures with repeating molecules surrounding a central core [206, 207]. They have high capacities for drug encapsulation and conjugation and the functionalization of surface groups [23, 208]. Besides, dendrimers are highly versatile in function and can be designed into multifunctional biological macromolecules by modifying the surface for various applications, which have been widely used in hydrophilic and lipophilic drugs delivery, nucleic acid delivery (gene, miRNA/siRNA), macromolecular delivery, and other biomedical applications [37, 209].

Astodrimer sodium (SPL7013) is a polyanionic dendrimer with antiviral activity. Romanowski et al. evaluated ocular tolerance and anti-adenovirus potency of topical SPL7013 in the rabbit eye model with adenovirus (HAdV5) ocular infections. In a tolerance study, rabbits were treated with 3% SPL7013, control, or 0.5% cidofovir an the Draize scale was used to evaluate the scores on 0, 1, 3, 4, 5, 7, 9, 11 and 14 days. Compared with the control, 3% SPL7013 and 0.5% cidofovir significantly shortened the duration of HAdV5 shedding. Moreover, 3% SPL7013 induced a Draize score of "minimal" to "almost no irritation". These findings suggest that 3% SPL7013 is suitable for treating adenoviral eye infections [210].

In a clinically relevant rat model of AMD, Kambhampati et al. discovered that systemic hydroxy-terminated poly-amidoamine dendrimer-triamcinolone acetonide conjugates.

(D-TA) were selectively taken up by activated microglia/macrophages and retinal pigmented epithelium, which are essential in disease progression. D-TA significantly inhibited choroidal neovascularization (> 80%, > 50-fold better than free drug). Meanwhile, in ex vivo studies of human postmortem diabetic eyes, dendrimers were also ingested into choroidal macrophages. These findings show systemic hydroxyl dendrimer drugs can be used alone or combined with current anti-vascular endothelial growth factors to provide a new approach to treating AMD [211].

**Fig. 9:**
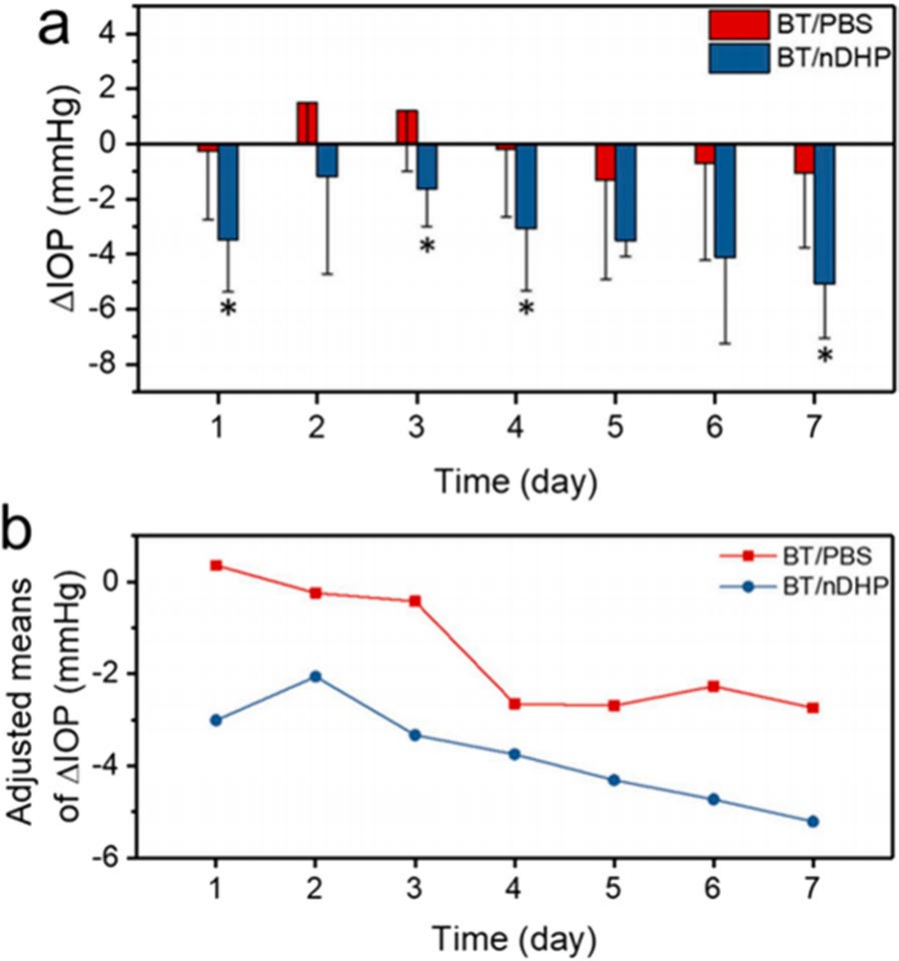
**a** The in vivo IOP of normotensive rats was reduced following 7 days of daily topical application of BT/nDHP and BT/PBS. * P < 0.05. **b** The daily adjusted averages of △IOP at 12 PM. Each formulation is administered as 2 × 5 μL of 0.1% w/v BT for 7 days

Recently, Wang and co-workers developed dendrimer gel particles (DHPs), which combine the advantages of dendrimers, hydrogels, and NPs. The delivery efficiency and efficacy of two anti-glaucoma drugs, brimonidine tartrate and timolol maleate, were tested by loading them into dendrimer gel particles of different sizes. The results showed that nano-in-nano DHP (nDHP, ~ 200 nm) was superior to μDHP3 (3 μm) and μDHP10 (9 μm) in terms of cytocompatibility, degradability, drug release kinetics, and corneal permeability. Compared with conventional drug solutions, nDHP increased drug corneal permeability by 17-fold. In addition, in vivo experiments showed that nDHP showed a significant IOP lowering effect after once daily administration for 7 days. The BT/nDHP reduced IOP by 4.5 mmHg in 4 h, which was 2.6 times more effective than BT/PBS eye drops on average. Besides, the IOP reduction in the BT/nDHP group was fourfold higher than that in the BT/PBS group at day 7 (Fig. 9). These findings indicate that nDHPs can be used for precision drug delivery and open a new window for combining multiple nanotechnologies [212].

In conclusion, dendrimers provide practical solutions to the solubility, distribution, and targeting problems faced by ocular drug delivery, making them effective carriers for ophthalmic applications. However, the clinical translation of this system is hampered by multiple formulation procedures, difficulties in large-scale production, cytotoxicity, and low drug loading [22]. A lot of research is still needed in the future.

### Liposomes

Liposomes are lipid vesicles consisting of one or more phospholipid bilayers with a central water compartment diameter of 0.025 to 10 µm [213]. Hydrophilic or lipophilic drugs can be encapsulated in them, which are widely used in the therapy of retinal diseases. For instance, verteporfin liposome is the first FDA-approved drug for treating AMD [214]. In addition, liposomes can adhere to the cornea, which are excellent carriers for drugs with low partition coefficient, low solubility, high molecular weight and poor absorption [215, 216]. The positive charge on the liposomes allows them to bind to the negatively charged mucin coating on the corneal epithelium. For example, the positively charged liposomes increased the trans-corneal flow of penicillin G fourfold, indicating enhanced corneal permeability [217].

Besides, Tavakoli et al. evaluated how the properties of these liposomes (particle size, surface charge, surface coating) affect their retinal penetration In an in vitro bovine explant system. The data indicate that small liposomes (≈50 nm) can penetrate the retina, whereas large liposomes (≈100 nm) cannot, underlining the importance of particle size. In addition, PEGylation and anionic surface charge favor the distribution of retinal liposomes. In conclusion, this study expands the understanding of the ocular barrier and provides valuable information for designing enhanced retinal drug delivery systems [215].

One study reported a cationic liposome eye drop loaded with tacrolimus (FK506) for treating dry eyes. Tacrolimus liposomes have a diameter of approximately 300 nm and a surface charge of + 30 mV. Cationic liposomes can interact with the anionic eye surface, prolonging the eye retention time and enhancing tacrolimus in the cornea.FK506 liposomes have also been shown to reduce ROS and DED-related inflammatory factors, which have excellent potential for treating ocular diseases [218].

Although liposomes have numerous advantages, limited drug loading capacity, short shelf life, and sterilization issues restrict their use [219].

### Niosomes

Niosomes are self-assembled vesicles formed by hydrating non-ionic surfactants, cholesterol, or other amphiphilic molecules [220]. They are structurally similar to liposomes and have been developed as an alternative delivery system to liposomes. The advantages of niosomes over liposomes include chemical stability, longer storage time, and continuous drug administration [37, 221]. Moreover, niosomes are biodegradable and non-immunogenic [222]. As a multifunctional drug delivery system, lipophilic and hydrophilic drugs can be encapsulated into membrane bodies with improved drug stability and bioavailability [223, 224].

Epalrestat is a drug that inhibits the polyol pathway and protects the diabetic eye from damage associated with sorbitol production and accumulation. Kattar et al. designed cationic ionophores composed of polysorbate60, cholesterol, and 1, 2-di-O-octadecyl-3-trimethylammonium propane to deliver the drug. Compared with contact lenses containing epalrestat or free drug solution, niosomes could encapsulate more drug (encapsulation efficiency 99.76%), increase the apparent solubility, protect the drug from premature degradation, and promote drug delivery to the intraocular tissues (75% drug release within 20 days). In addition, drugs encapsulated in niosomes show better biocompatibility. Hence, the niosomes are expected to encapsulate and carry therapeutic drugs through the eye to meet the requirements of a controlled drug system for treating diabetic eyes [225].

To better treat glaucoma, Allam et al. mixed betaxolol-loaded niosomes into pH-responsive in situ gels to further prolong precorneal drug retention. The optimized niosomes had a high encapsulation efficiency (69 ± 4.8%), a negative surface charge, and a nanoscale hydrodynamic diameter. After the instillation of the niosomal gel loaded with betaxolol into rabbit eyes, IOP was consistently reduced, and the relative bioavailability of betaxolol was significantly increased (280 and 254.7%) compared with commercially available eye drops. Therefore, using niosomal pH-triggered in situ gel for ophthalmic drug delivery is a promising glaucoma treatment technique [226].

Similarly, Fathalla et al. incorporated latanoprost niosomes into gels to prolong the anti-glaucoma effect of latanoprost. Non-specific interactions of latanoprost with the surfactant resulted in more than 88% drug encapsulation efficiency. This gel reduced IOP in normotensive rabbits for 3 days, with prolonged release and no irritating effect on rabbit eyes compared with normal Xalatan^®^ eye drops. The study's results confirmed the potential of latanoprost niosomal gel to prolong drug release, reduce the frequency of administration, and possibly improve patient compliance [227].

Despite the many advantages of niosomes, low drug loading, encapsulated drug leakage, physical instability, and high production cost limit the application of niosomes in drug delivery [22]. These are complex challenges that need to be addressed in the future.

### Nanowafers

The nanowafers are small transparent disks that can be applied to the eye's surface with a fingertip and withstand continuous blinks without displacement. The slow drug release from the nanowafers prolongs the retention time of the drug on the ocular surface and facilitates drug absorption [228]. Coursey and co-workers have developed a dexamethasone-loaded nanowafer (Dex-NW) for the therapy of DED. In the experimental mouse dry eye model, administering only two doses of Dex-NW over a 5-day treatment period was comparable to the efficacy of topical Dex eye drops administered twice daily during the same treatment period. Dex-NW showed better therapeutic effects than topical Dex eye drops, confirming the efficacy and translational potential of the nanochip drug delivery system for DED [228]. In addition, nanowafers can also be used as protective membranes for corneal surface damage in DED [229].

Furthermore, Yuan et al. demonstrated the in vivo efficacy of axitinib-loaded nanowafers in treating corneal neovascularization in a mouse eye burn model. Laser scanning confocal imaging and reverse transcription-polymerase chain reaction studies have shown that once-daily administration of axitinib-loaded nanowater was twice as effective as topical eye drops twice a day [230].

Recently, a study reported PVA nanowafers loaded with PnPP-19, a synthetic peptide designed from a toxin existing in the spider's venom and having a hypotensive effect on the eyes of rats. Compared to common eye drops. the device prolonged the delivery time of the peptide on the ocular surface and maintained its fluorescence intensity for more than 180 min. Besides, PVA nanowafers could enhance PnPP-19 diffusion into the eye tissues, with continued fluorescent on the cornea after 24 h. These findings prove the potential of nanowafers to treat glaucoma [231].

To date, the polymers and drugs used to develop the nanowafers are already in clinical use. Besides, the nanowafers can be easily dropped onto the eye surface through the fingertips of patients without any clinical procedures. Therefore, it’s promising that the nanowafers can be quickly translated into clinical trials for human use.

### Contact lenses

Contact lenses are hard or soft polymer devices that fit the cornea to correct refractive errors. They can be composed of hydrophilic or hydrophobic polymers [232]. Based on the designed materials, there are two main types of contact lenses: soft contact lenses, which are made of hydrogels or silicone hydrogel polymers, and rigid gas-permeable contact lenses [233]. Drug-loaded contact lenses can be in close contact with the cornea, prolong drug retention time, and improve ocular bioavailability by at least 50% [234, 235]. Therapeutic contact lenses can decrease the required drug dose, frequency of administration, and systemic drug absorption [236]. However, water content, oxygen permeability, transparency, and mechanical property pose challenges for drug delivery, especially for patients who are strange to wear contact lenses [237]. The combination of nanotechnology and contact lenses has revolutionized drug delivery in the eye.

Immersion of contact lenses in drug-containing NPs (preferably < 100 nm) is the most common, simplest, and most cost-effective method of manufacture [238]. For example, contact lenses immersed in zinc oxide NPs (20–40 nm) showed antibacterial activity against ocular microorganisms such as Staphylococcus aureus, Bacillus subtilis, Pseudomonas aeruginosa and Escherichia coli [239]. In addition, NPs containing drugs can be coated on the surface of the contact lens. Sahadan et al. developed a silicone hydrogel contact lens coated with phomopsidione NPs that allowed the sustained release of phomopsidione for 48 h and could be used to treat keratitis [240].

Likewise, Jiao et al. used a novel polyacrylamide semi-interpenetrating network hydrogel consisting of quaternary ammonium chitosan and tannic acid to construct a novel antibacterial and antioxidant contact lens. The antibacterial test showed that the contact lens had a good bactericidal effect on Staphylococcus aureus and Escherichia coli (almost 100%). Besides, tannic acid could alleviate oxidative stress and protect cells from ROS-induced cytotoxicity. Hence, this drug-free antibacterial and antioxidant contact lens is a promising option for treating ocular infectious and inflammatory diseases [241].

Ding et al. developed a contact lens device with embedded microtubes to treat glaucoma. This device can improve drug bioavailability, decrease the risk of adverse effects and prolong drug release time for 45 days. More importantly, as IOP fluctuates, the curvature of the contact lens changes, which in turn triggers more drug release, making it an adaptive drug-release device that potentially provides dynamic and adaptive anti-glaucoma treatment [242]. It is believed that in the future, the combination of contact lenses and nanotechnology will have more applications in the therapy of ophthalmic diseases.

### Hydrogels

Hydrogels comprise a three-dimensional network of hydrophilic polymer chains with high water retention capacity. In situ, gels are administered as a liquid and transformed into a gel upon eye contact [243]. Heat-responsive, pH-responsive, and ion-responsive materials are the three primary stimulation-responsive materials most widely employed to develop gel systems for ocular medication administration. Recent hydrogel advances offer great opportunities for ophthalmic drug delivery to treat ocular diseases [244, 245]. Since hydrogels can improve the therapeutic effect of ophthalmic drugs through the following mechanisms, including (1) prolonging the retention time of drugs at the site of drug delivery, (2) sustained drug release at the target site, and (3) the co-delivery of multiple drugs to their function [97, 116, 246, 247].

The combination of nanotechnology and hydrogels has significantly progressed the treatment of ocular diseases [18]. Various nanoformulations such as NPs, nanomicelles, MNs, and nanofibers have been combined to prepare composite systems to further prolong the retention time of drugs on the ocular surface and improve their bioavailability [248]. Some representative hydrogels used in ocular drug delivery will be detailed in the following sections and emphasized with a few appealing examples.

Fang et al. developed a polypseudorotaxane hydrogel for treating anterior uveitis by mixing Soluplus micelles (99.4 nm) with cyclodextrins solutions. The optimized hydrogel exhibited shear thinning and sustained release properties. In the endotoxin-induced rabbit uveitis model, the hydrogel significantly improved the drug retention ability (21.2 folds), corneal permeability (1.84 folds), intraocular bioavailability (17.8 folds), and anti-inflammatory effect compared with drug solutions. In addition, cytotoxicity and eye irritation studies also confirmed the good biocompatibility of the hydrogel. In conclusion, this study demonstrated that γ- cyclodextrins-based hydrogels have great potential for treating anterior uveitis [249].

Patients with wet AMD require an intravitreal injection of Bev or other drugs. Jung et al. developed an in situ formed hydrogel consisting of Bev and hyaluronic acid cross-linked to poly (ethylene glycol) diacrylate, which was slowly released after Bev injection into the suprachoroidal space of the eye using MNs. The in-situ formed Bev-hyaluronic acid hydrogel was well tolerated and released Bev for over 6 months in the rabbit eye, which could be used in treating posterior ocular diseases in the future [250].

**Fig. 10:**
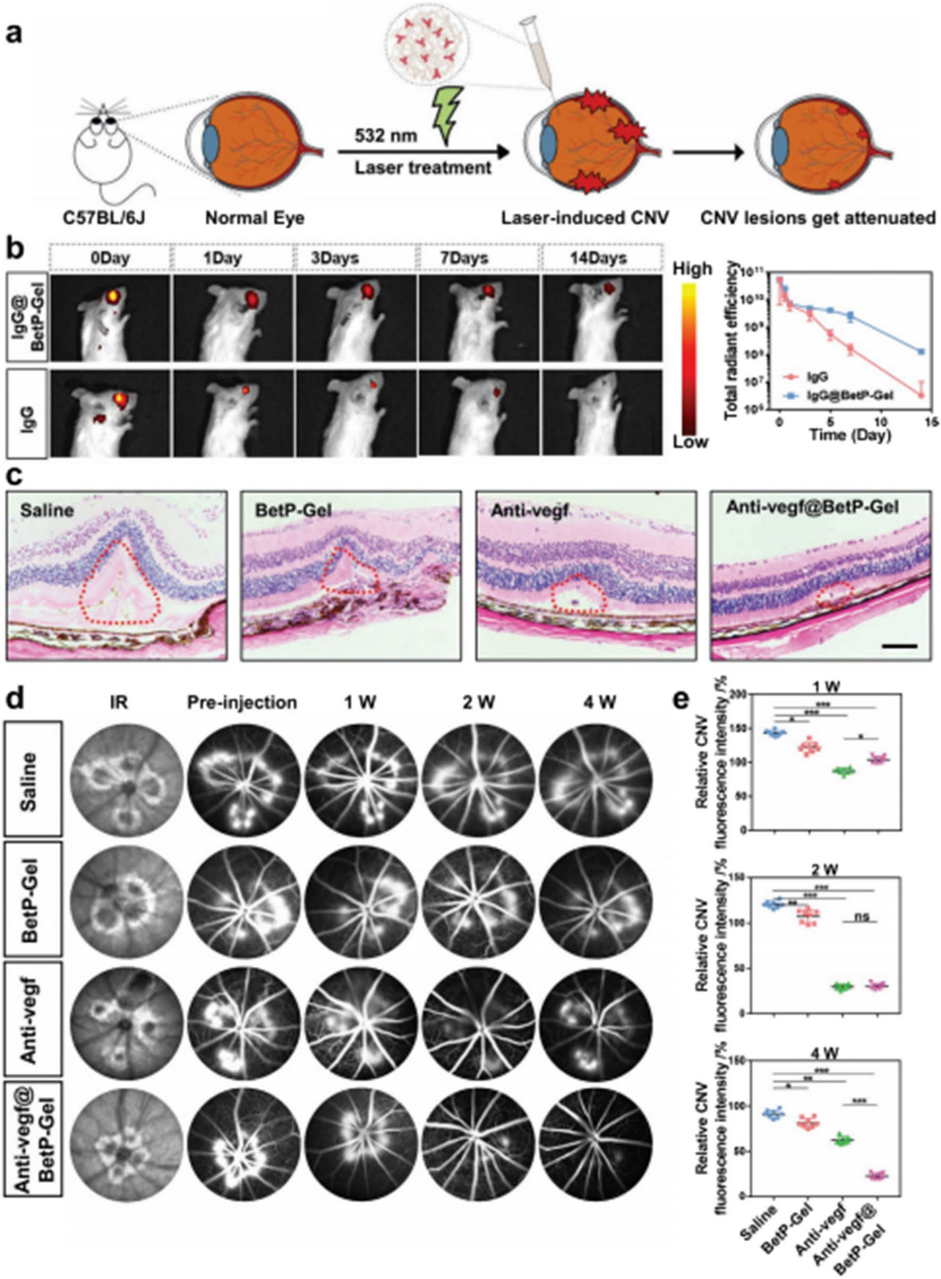
The long-term effect of the laser-induced mice CNV model using Anti-VEGF@BetP-Gel. **a** Experimental design to evaluate the impact of Anti-VEGF@BetP-Gel. **b** Fluorescence IVIS imaging demonstrating the in vivo retention of IgG-Cy5.5 at various time periods after intravitreal injection of free IgG-Cy5.5 or IgG-Cy5.5@BetP-Gel. **c** H&E-stained transverse CNV sections after 4 weeks intravitreal injection. **d** The typical fluorescein fundus angiography images of laser-induced mice CNV model taken at 1, 2, and 4 weeks following intravitreal injection. **e** The graded and measured angiogenic vascular leakage values

Recently, Gao et al. developed an injectable antibody-loaded supramolecular nanofiber hydrogel by mixing betamethasone phosphate, the gold-standard anti-VEGF agent for AMD, with CaCl2. This betamethasone phosphate-based hydrogel can release anti-VEGF to inhibit retinal vascular proliferation, attenuate CNV for a long time, and remove ROS to reduce local inflammation (Fig. 10). Notably, the duration of anti-VEGF can be effective for approximately threefold longer than conventional administration, can reduce the frequency of administration and improve patient compliance [251].

In short, combining hydrogels and nanotechnology expands the range of biomedical applications and opens new windows for ocular drug delivery.

### Microneedles

Microneedle technology is an attractive, minimally invasive strategy with the advantages of easy drug administration, controlled drug release, and low manufacturing cost [252]. It has been widely studied for transdermal delivery of various therapeutic drugs (e.g., anti-diabetes, anti-obesity drugs, and vaccines) [253]. Various MNs have been exploited and tested, such as solid MNs, hollow MNs, and dissolved MNs [254, 255]. Due to its excellent patient tolerance and efficacy have prompted researchers and pharmacists to explore its use in treating ocular diseases.

Fungal keratitis (FK), an infectious corneal disease, is a serious cause of visual impairment worldwide. Shi et al. manufactured a dissolved microneedle array patch based on PLA and hyaluronic acid to treat FK. Among them, a 30% PLA-hyaluronic acid MN patch reversibly penetrated the corneal epithelial layer, and the cornea recovered completely within 12 h. More importantly, it demonstrated that the therapeutic effect of self-implantation of drug-loaded MN patches as a controlled release reservoir for local drug delivery is much better than that of eye drops in the rabbit model of FK. Hence, the MN patch serves as an ocular drug delivery system with efficient and rapid corneal healing ability, which may also open a new avenue for the clinical treatment of FK [256].

Besides, Cui et al. developed cryo-MNs for the ocular delivery of living bacteria. In cell experiments, the device delivered predatory Bdellovibrio bacteriovorus, which could successfully inhibit the proliferation of gram-negative bacteria. In a mouse ocular infection model, infection was reduced by nearly six-fold after 2.5 days of treatment, and corneal thickness and morphology were unaffected; this brings new insights for the safe and effective delivery of novel antimicrobial agents to the impermeable ocular surface [257].

Lee et al. developed a self-plugging MN (SPM) to perform intraocular drug delivery and seal the scleral tissues at the same time. SPMs were fabricated by a thermal stretching process and then coated with a drug-loaded polymer carrier and a biocompatible hydrogel. Each coating functional layer was characterized and explained in vitro and ex vivo experiments. The 10 mm-long SPM released over 95% of the coated drug (27.9 μg) gradually within 24 h. Furthermore, the ability of SPM to achieve rapid closure and sustained intraocular delivery was confirmed using a porcine model [258].

However, MN products' performance and quality evaluation involves several vital technical parameters, such as bending property, loading capacity, and safety in use. At the same time, MNs can cause tissue damage and have high technical requirements for clinicians, so there is still a long distance to realize the clinical transformation of MNs.

## Other promising ocular drug delivery methods

### Gene therapy

Gene therapy is a hot topic in the research of modern ophthalmic diseases. There are two strategies for gene therapy: (1) restoring the function of nonfunctional or missing proteins (gene addition or gene editing) and (2) knocking down proteins to block their function (gene silencing) [259].

The eye has important features well suited for gene therapy: well-defined anatomy, relative immunological privilege, accessibility, simplicity of diagnosis, and one eye can be used as an experimental target and the other as a control in the same subject [259]. There are more than 350 hereditary eye diseases, including choroiditis, retinitis pigmentosa, Leber congenital amaurosis, etc., involving various genetic loci [260, 261]. In addition, gene therapy approaches are also being exploited and extended to diseases not unrelated to a single genetic defect, such as corneal and retinal vascular disease or AMD [262, 263]. Gene delivery systems primarily include viral vectors, non-viral vectors, gene editing techniques (mainly CRISPR-Cas9), and epigenetic treatments with antisense oligonucleotide (ASO) and RNAi therapeutics [264].

### Viral vectors

Viral vectors are often therapeutic gene vectors due to their high transduction efficiency. Several viral vectors, such as adenovirus, adeno-associated virus (AAV), retrovirus and lentivirus, have been widely used in ocular gene therapy [265, 266].

Among them, AAVs are tiny (~ 26 nm diameter), non-enveloped, icosahedral-structured capsid, single-stranded DNA, non-pathogenic viruses and the most common viral vectors used for ocular gene therapy [267]. Both dividing and nondividing cells can be transduced with AAV vectors. They are not integrated into the host cell genome but live in the cells as free DNA [267]. Recombinant AAVs can deliver various genetic materials to control protein expression within cells and alleviate some ocular disorders. The FDA has approved two human-using AAV-based gene therapies, and many are in clinical trials [268]. A study employing AAV as a vector may alleviate the problems of repeated intravenous injections to induce a systemic blockade of VEGF-A, which is normally expressed in human retina [269]. Anti-angiogenic microRNAs have also been used to lessen the number of corneal neovascularization through recombinant adeno-associated viruses multi-targeted biotherapy [270].

However, viral vectors have some limitations, such as potential mutagenesis, limited loading capacity (< 5 kb for AAV), poor immunoreactivity, and high production costs, resulting in unaffordability for patients [271]. Therefore, several alternative strategies, such as non-viral vector systems and NPs, are being developed.

### Non‑viral vectors

Compared with viral vectors, non-viral vectors are less immunogenic and pathogenic [272]. Besides, non-viral vectors are generally low-cost, easy to manufacture, and the size of the involved genes is unrestricted. [273]. Many non-viral vectors, such as NPs, dendrimers, liposomes, polymers, naked DNA, and peptide-based vectors, have been used for gene therapy in the eye [168, 264].

For example, Ma et al. fabricated redox-responsive quasi-mesoporous magnetic nanospheres (rMMNs) with an iron oxide core and disulfide bond-bridged polyethyleneimine shell. These rMMNs are highly loaded with miR-30a-5p through electrostatic interactions and then efficiently release miRNA under a glutathione-dominant microenvironment. The rMMNs up-regulate the level of miR-30a-5p by targeting the transcription factor E2F7 and inhibiting the malignant phenotype of ocular melanoma. In addition, rMMNs play a role in promoting cancer cell apoptosis by regulating M1-like macrophage polarization and activating the Fenton reaction. Therefore, rMMNs are attractive miRNA vectors for gene therapy and can enhance pro-inflammatory immunity in melanoma and other cancers [274].

In a notable study by Ribeiro et al., lipoplexes were developed utilizing sodium alginate as an adjuvant and strategically coated with hyaluronic acid (HA-LIP). This innovative approach facilitated siRNA delivery to retinal cells, inhibiting Casp3 expression and attenuating retinal degeneration caused by excessive LED light exposure. The safety of HA-LIP was confirmed through electroretinogram measurements, clinical assessments, and histology. These findings highlight the potential of HA-LIP as a non-viral vector for siRNA delivery, opening up promising avenues for treating various retinal diseases [275].

### Antisense oligonucleotide, RNAi, CRISPR-Cas9

ASOs are brief (12–24 nt) single-stranded nucleic acids (DNA or RNA) that bind to specific complementary mRNA targets by Watson–Crick base pairing to regulate gene expression. [276]. In a phase 1b/2 clinical trial of Leber congenital amaurosis type 10, intravitreal antisense oligonucleotide sepofarsen had a manageable safety profile and showed clinically relevant visual improvement [277].

**Fig. 11:**
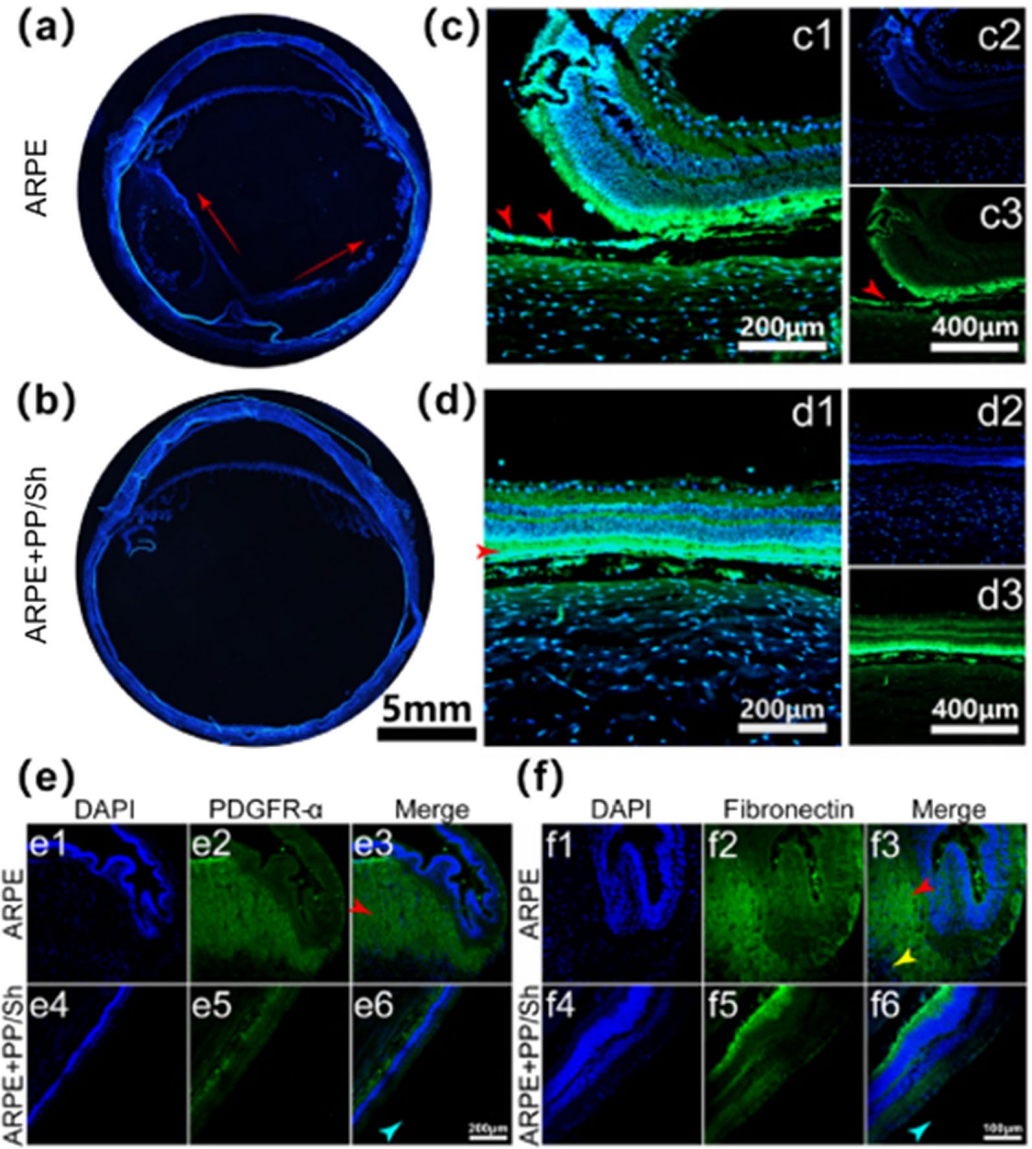
Representative images of the immunofluorescence assays. **a**, **b** The nuclei are stained blue with DAPI, the fibrous membrane of the vitreous cavity is marked by the red arrows. **c**, **d** The red arrows indicate the retinal pigment cell layer. **e**, **f** The expression of PDGFR-α and Fibronectin

The RNAi pathway is expressed through sequence-specific double-stranded RNA genes. RNAi regulates mRNA stability and cell translation using double-stranded small interfering RNA (siRNA) or short hairpin RNA (shRNA) complementary to their target RNA [276]. Among them, siRNA is a nucleotide duplex of about 20 bp in length, which can specifically couple and guide the degradation of target genes in cells, modify the relevant signal pathways for therapeutic intervention [278]. Wang et al. used polyethene glycol grafted branched polyethyleneimine as a non-viral gene vector to interfere with platelet-derived growth factor alpha receptor and block the epithelial-mesenchymal transition process of cells by gene silencing technology to achieve an anti-fibroblast effect (Fig. 11). This study provides a feasible and promising clinical idea for using RNAi technology to develop non-viral gene vectors to prevent fibroblast eye disease [279].

The CRISPR-Cas9 system is an engineered endonuclease directed by a short RNA comprising a complementary region with 20-nucleotides long that can recognize target DNA sites through complementary base pairing and precisely create nicks or cuts in the genome [280]. The incision is then repaired using non-homologous end-joining mechanisms (NHEJ) or homology-directed repair (HDR) [281]. Due to its simple structure, it has become the most popular genome editing tool and has gained utility in disease modelling, genetic screening, epigenome editing, cell tagging, and gene therapy applications [282].

X-linked form of hereditary retinal degeneration caused by mutations in the Retinitis Pigmentosa GTPase Regulator (RPGR) gene is a challenge for gene therapy. The majority are frameshift mutations in the OFR15 exon, a highly repetitive, purine-rich region of the RPGR. The RPGR reading frame may be restored in partly mutated photoreceptors using CRISPR/Cas9 targeted ablation of ORF15, followed by repair via non-homologous end joining, which then corrects gene function in vivo [283]. Besides, CRISPR-Cas9 with guide RNAs increased VEGF gene ablation in human cells in vitro with a slight increase in off-target activity and demonstrated the possibility of CRISPR-Cas9 causing genetic ablation in vivo, which might be affected by multiple factors in the body [284].

Recently, the delivery of gene editing agents as ribonucleoproteins in vivo has been shown to have a safety advantage over nucleic acid delivery. Banskota et al. developed engineered DNA-free virus-like particles (eVLPs) that efficiently package and deliver the base editor or Cas9 ribonucleoprotein. A single injection of eVLPs into mice achieved therapeutic levels of base editing in multiple tissues, such as reducing serum Pcsk9 levels by 78% after 63% liver editing and partially restoring visual function in a mouse model of genetic blindness. The eVLPs combine the critical advantages of viral and non-viral delivery and are promising vectors for therapeutic macromolecular delivery [285].

In conclusion, advances in the field of gene therapy and gene editing have brought hope for the treatment of eye diseases. At the same time, with the development of nanotechnology, the prospect of gene therapy for eye diseases will be broader and clinical transformation will be achieved faster.

### Exosomes

**Fig. 12:**
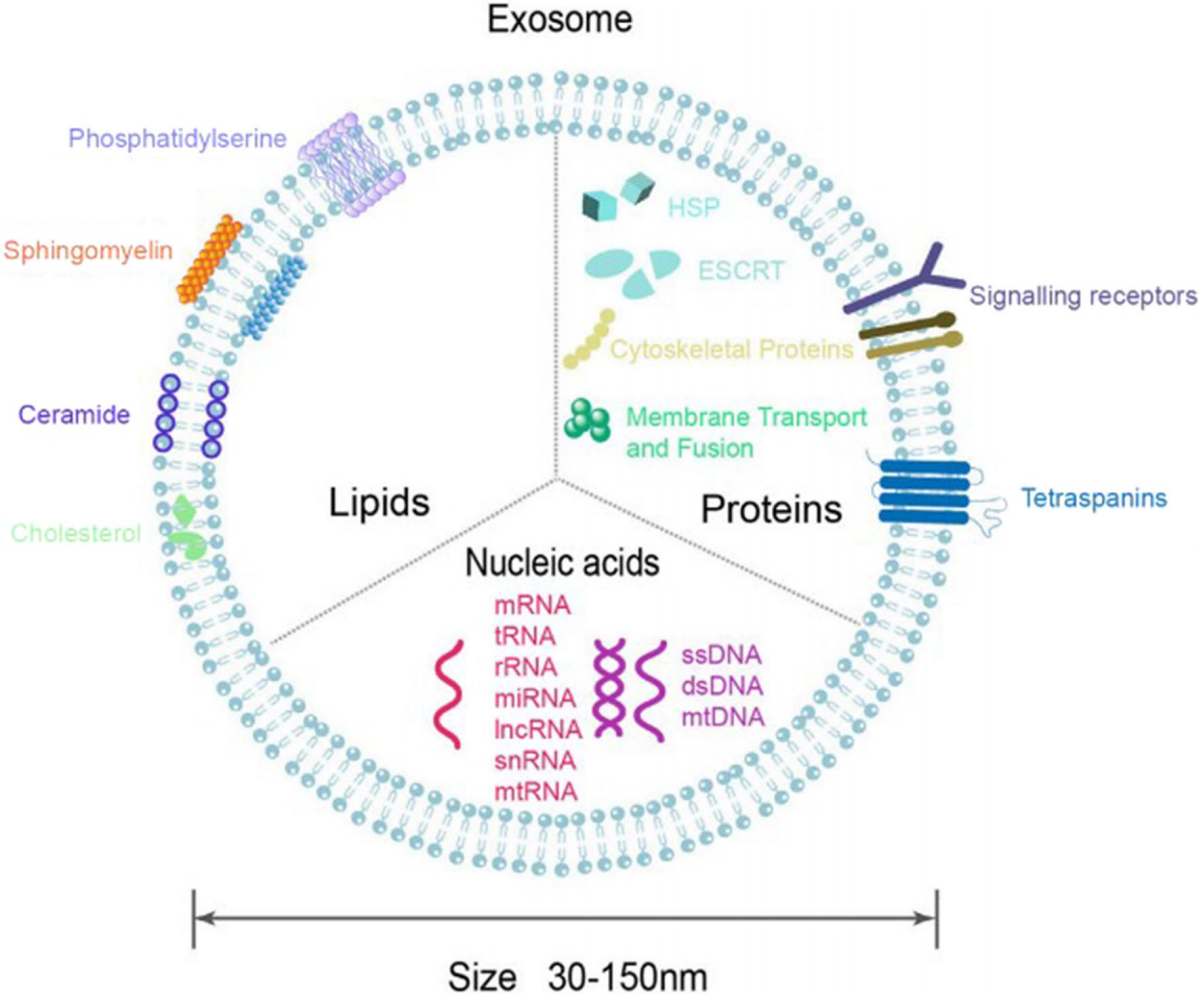
Diagram of exosomal molecular composition. Reprinted with permission. Copyright 2021, Cell Commun Signal

Exosomes are nanoscale vesicles with a 30–150 nm diameter and consist of lipid bilayers, proteins, and genetic material [286]. The diagram of exosomal molecular composition is shown in Fig. 12 [287]. Almost all types of cells in the human body secrete exosomes, which act as a vital role in intercellular communication, inflammatory response and immune regulation [288]. Due to their natural, non-toxic, and biodegradable properties, exosome is an ideal candidate for drug delivery to treat many diseases, such as cancer, cardiovascular diseases, neurodegenerative diseases, and so on [289, 290]. As natural carriers, exosomes have a higher barrier-crossing ability and safety than synthetic nano-drug carriers and are more capable of delivering various drugs or bioactive molecules among eye diseases.

Pathological angiogenesis is a hallmark of numerous vision-threatening diseases. Dong et al. used exosome as a carrier for intraocular delivery of the anti-angiogenic peptide KV11 into the retinal vasculature by retroorbital injection, which greatly enhanced the inhibitory effect of KV11 on neovascularization. Besides, this is a less invasive modality compared to intravitreal injection. Therefore, EXO_KV11_ may be an effective nanotherapeutic agent for treating pathological angiogenesis in retinopathy [291].

In addition, Tian et al. engineered exosomes derived from regulatory Treg cells (rEXS) and used a peptide linker to conjugate them with anti-VEGF antibodies (aV). This nano-drug exploited the ability of rEXS to localize to neovascular lesions. Meanwhile, the peptide adaptor was cleaved by matrix metalloproteinases in inflammatory lesions and released rEXS and aV to inhibit inflammation and the activity of VEGF. In mouse and nonhuman primate CNV models, aV binding to rEXS resulted in a five-fold longer intraocular retention time than soluble proteins. Besides, a 3.5-fold increase in aV accumulation was detected in CNV lesions, and the controlled release of aV by matrix metalloproteinases-mediated cleavage also contributes to the efficacy. These findings provide new ideas for more effective treatment of CNV [292].

Also, Zhou et al. reported that exosomes derived from mesenchymal stromal cells (MSC-exo) were administered as eye drops. In a mouse model of DED, MSC-exos reprogram pro-inflammatory M1 macrophages to an immunosuppressive phenotype through Mir-204-mediated IL-6/IL-6R/Stat3 pathway targeting, facilitating dry eye therapy. MSC-exo maintains ocular surface homeostasis and corneal transparency by regulating the balance between M1 and M2 macrophages on the ocular surface [293]. Furthermore, exosomes derived from the umbilical cord mesenchymal stem are used in clinical trials to alleviate dry eye symptoms (NCT04213248).

Exosomes are a subtype of extracellular vesicle (EV). To avoid alterations in tear balance, tear film stability, pH and osmolality changes [294], researchers are exploring new approaches, including fusing drug-carrying liposomes with EVs to improve bioavailability [295]. Notably, EVs designed to be produced by implanted cells have recently been reported [296]. This technique provides a unique route for the production of engineered exosomes in vivo.

In summary, exosomes exhibit key characteristics that make them highly attractive as therapeutic drug carriers, particularly their ability to target multiple therapeutic payloads, benign safety, and low immunogenicity potential. However, the optimal isolation method for specific exosome applications, the heterogeneity of extracellular vesicle preparations, and the availability of appropriate methods and equipment still require extensive studies to verify.

### Self-nano emulsifying drug delivery systems

SNEDDS are mixtures of oil phases, surfactants, and cosurfactants or cosolvents [297]. After aqueous phase dispersion and slight agitation, SNEDDS can spontaneously form oil-in-water NEs with droplet sizes below 200 nm [298]. Moreover, spontaneous emulsification occurs when the entropy change favoring dispersion surpasses the energy needed to expand the surface area of the dispersion [299, 300]

The surfactant and lipid components used in SNEDDS can synergistically promote the absorption of drugs in the gastrointestinal tract, which is one of the emerging strategies to improve the availability of oral administration. Furthermore, these components can be easily modified to make SNEDDS suitable for hydrophilic and hydrophobic drugs [301]. For example, Lopez-Cano et al. prepared a self-emulsified osmo-protective ophthalmic microemulsion (O/A) with an internal oily phase (1.2%), an external aqueous phase (96.3%), cosolvents (1%), and surfactants (1.5%). Scanning electron microscopy and cryo-transmission electron microscopy demonstrated that all formulations exhibited sphere-shaped morphology with good cell tolerance (≈100%) and could be stable at 8 ℃ for 9 months. These findings manifest that self-emulsified microemulsions can be a novel drug delivery system for treating ocular diseases [302].

Besides, to improve the bioavailability of amphotericin B (AmpB), which is one of the most commonly used drugs for treating severe fungal infections, Kontogiannidou et al. prepared an oral formulation by combining AmpB-loaded SNEDDS with room temperature ionic liquids of imidazolium. This hybrid system enhanced the solubility of AmpB and exhibited good biocompatibility [303].

Although SNEDDS show considerable advantages over conventional drug delivery systems, there are still some limitations that require further investigation:(1) the content of vehicles in SNEDDS is usually very high, and the safety should be considered, (2) the risk of drug precipitation, (3) the capacity of improving drug loading and targeting, (4) knowledge of in vivo pharmacokinetics of SNEDDS is still a grey area, especially in human volunteers [304]. It is believed that SNEDDS will be more widely used in ophthalmology in the future.

## Characterization of nanotechnology-based drug delivery systems

Nanotechnology refers to treating structures at the nanoscale level, which ranges in size from 1 to 100 nm and is proportionally comparable to peptide drugs [17]. Their basic physicochemical properties, such as visual appearance, size, zeta potential, refractive index, pH, retention, viscosity, osmolality, biodegradability, surface charge, hydrophobicity and biodegradability are closely related to their therapeutic efficacy in the ocular pathological environment [23, 305]. Therefore, we characterized nanocarriers' physicochemical and biological properties to provide new ideas for better design of effective novel delivery systems.

### Visual appearance

Most NPs present a transparent, translucent, or translucent to milky appearance, depending on the size of the particle, the surfactant or cosurfactant, and the concentration or type of oil. The transparency of nanocarriers can be checked by measuring the transmittance (%T) at 520 nm using a UV spectrophotometer [61, 306].

### Particle size (PS) and polydispersity index (PDI)

PS and PDI are essential characteristics of nanocarriers and the main determinants of physical stability. These parameters are mainly estimated by dynamic light scattering or photon correlation spectroscopy [61, 307]. Particles with a smaller size penetrate the inner mucin layer of the tear film more quickly and have higher aqueous humor absorption than larger particles [307, 308]. For PDI, 0 represents a homogeneous system, and 1 represents a heterogeneous system [309]. PDI values less than 0.1 and close to 1 indicate good and poor quality of the colloidal system, respectively [61]. Small PS and PDI are generally preferred for ocular drug delivery because they provide better stability, biodistribution properties and high patient compliance [23].

### Morphology

Microscopic techniques were used to study the morphology of nanocarriers. Electron microscopy approaches, including transmission electron microscopy (TEM), freeze-fracture transmission electron microscopy (FF-TEM), and negative staining transmission electron microscopy (NS-TEM), are preferred for liquid samples, while scanning electron microscopy is used for solid samples [220]. In addition, TEM and atomic force microscopy (AFM) techniques can be used to reconfirm the results obtained from photon correlation spectroscopy or dynamic light scattering measurements [310].

### Zeta Potential (ZP)

Zeta potential is an indicator of the physical stability of a nano-system. It is determined by the electrophoretic motion of particles in an electric field and is one of the most studied parameters [311]. The surface potential is able to be measured using the laser Doppler anemometry, and the magnitude of ZP indicates the degree of electrostatic repulsion between two neighboring particles [220]. Typically, a zeta potential of about ± 20 mV is suitable for electrostatic attachment to the corneal surface [61]. High zeta potential values (> ± 30 mV) can stabilize the nanoformulations by electrostatic repulsion [23]. Besides, positively charged particles are more suitable for enhancing electrostatic interactions with negatively charged ocular surfaces, showing better bioavailability and activity [312, 313].

### Stability

Stability issues such as creaming, flocculation, Ostwald ripening, coalescence, and precipitation are essential obstacles in developing nanocarriers [23]. The stability of different nano-systems can be estimated by short-term stability (3 months), centrifugation test, freeze–thaw cycle, heating–cooling cycle and high-temperature storage [314]. A promising approach to improve biological stability is pegylated. As a hydrophilic non-ionic polymer with high chain flexibility, PEG-coated or coupled on the surface of nanocarriers can prevent macrophage clearance by reducing contact with the surrounding environment (oxidants, enzymes, and other degraders) [315–317]. Besides, in vivo drug flux studies have shown that pegylated nanostructured lipid carriers have nearly twofold higher levels of ciprofloxacin in all ocular tissues than non-pegylated nanostructured lipid carriers at 2 h after administration [315].

### Refractive index (RI)

Refractive index is measured by Abbe’s refractometer to determine soft contact lenses' water content, salinity and sugar concentration [318]. The tear RI was generally between 1.340 and 1.360. Therefore, the recommended RI value for ocular formulations must be < 1.476 [319, 320]. For instance, the RI values of intraocular NEs prepared by Ismail et al. ranged from 1.334 to 1.338, which was satisfactory to meet the demands [179].

### pH

pH measurement plays a critical role in preparing stable and non-irritating ocular formulations. It has been reported that acidic (pH < 4) or alkaline (pH > 10) solutions can cause chemical damage to the eye [61]. Therefore, the appropriate pH of topical ophthalmic formulations ranges from 6.6 to 7.8 [321]. Compared with Travatan^®^ eye drops, the pH value of the prepared NEs is between 5.5 and 5.9, which is suitable for ocular instillation and can treat DED [179].

### Retention

Ocular retention is a fundamental property of ocular delivery systems because it prolongs the duration of drug action, reduces the frequency of drug administration, and improves drug bioavailability [17]. Nanosystems with larger surface areas, such as thin films, hydrogels, have longer diffusion and contact time on the corneal surface, which enhance eye retention. In general, γ-scintigraphy, texture analysis, fluorescence imaging and surface plasmon resonance spectroscopy are used to determine the intraocular retention of nano preparations [17, 61, 322].

### Viscosity

The viscosity of ocular preparations is generally less than 20.0 mPa [323], while the appropriate viscosity of ocular preparations is generally 2–3 mPa [311]. It was reported that the nano-formulations with higher viscosity and lower surface tension could prolong retention times [324]. Synthetic polymers (such as polyacrylate and PVA) and natural polymers (such as hyaluronic acid, alginate) can be used as viscosity enhancers. For example, in vivo anterior corneal retention assay showed that the Chitosan Oligosaccharides-coated nanostructured lipid carrier increased 7.7-fold compared with the uncoated lipid carrier [325].

### Osmolality/Isotonicity

Osmolality was determined based on four properties of ocular or tears formulation parameters known as vapor pressure, osmotic pressure, boiling point, and freezing point [326]. In addition, osmolality can also be measured in terms of the number of moles of solution per liter or kilogram [327]. It was reported that ocular preparations with osmolarity less than 100 mOsm/kg or more than 640 mOsm/kg were named as eye irritants depending on the droplet volume [61].

### Drug loading and release

Drug loading and release are essential to the ocular drug delivery system. Nanocarriers require a high drug payload, which can improve biocompatibility and achieve better therapeutic effects [94]. The primary determinant of drug load is drug solubility. The drug is released continuously in nanocapsules with high encapsulation efficiency, and the release rate is critical to achieve an effective therapeutic effect and avoid drug toxicity. [328]. Pharmacokinetics can be studied via a series of in vivo and in vitro experiments. For example, the content of drugs can be detected in tears and aqueous humor through ELISA (Enzyme-linked Immunosorbent Assay) or HPLC (High Performance Liquid Chromatography) in vitro [329, 330]. Alternatively, fluorescence-labeled drugs could be used and then detected by confocal microscopy in vivo [331]. Besides, the results can be analyzed by some pharmacokinetic parameters, such as the maximum drug concentration (Cmax), the time required to reach Cmax (Tmax), and the area under the concentration–time curve (AUC0-t) [332].

### Biocompatibility and safety

Biocompatibility and safety are critical for nanocarriers. The primary safety concerns of nano-formulation arise from the surfactants and cationic lipids used in the formulation, which may damage corneal epithelial cells during long-term use [333–335]. The safety of eye preparations was evaluated by various tests, such as HEM-CAM test, Schimer's test, Draize's test, histopathological studies and cell viability studies [23]. Using surfactants and cationic lipids may create safety issues that should be further optimized and improved during development [333]. In the HEM-CAM test, ocular toxicity and irritation were predicted by observing the changes in blood vessels [336].

## Approval and under clinical status of nanotechnology-based delivery systems for ocular diseases

With the increasing number of products on the market, the development of nanotechnology for the treatment of ocular diseases seems promising. Table 2 lists some FDA-approved nanocarriers for ocular diseases.

**Table 2:** Some FDA-approved nanocarriers for the treatment of ocular diseases

Product	Nanocarriers	Drug	Indications	Approval date
Visudyne^®^	Liposome	Verteporfin	Wet age macular degeneration	2000
Durezol^®^	Nanoemulsion	Difluprednate	Postoperative ocular inflammation	2002
Restasis^®^	Nanoemulsion	Cyclosporine A	Dry eye disease	2002
Retisert^®^	Implant	Fluclorolone	Uveitis and macular edema	2005
Triesence^®^	Nanoparticles	Triamcinolone acetonide	Dry eye disease	2007
AzaSite^®^	Micelles	Azithromycin	DED; keratitis; eye inflammation	2007
Durezol^®^	Nanoemulsion	Difluprednate	Eye infection and pain	2008
Cationorm^®^	Nanoemulsion	Medical device	Dry eye disease	2008
Trivaris^™^	Nanoparticles	Triamcinolone acetonide	Uveitis	2008
Besivance^®^	Nanosuspension	Besifloxacin	Ocular bacterial infection	2009
Tobradex ST^®^	Nanosuspension	Tobramycin Dexamethasone	Ocular inflammation and bacterial infection	2009
Ikervis^®^	Nanoemulsion	Cyclosporine A	Keratitis	2015
BromSite^®^	Solution	Bromfenac	Postoperative inflammation and pain	2016
Cequa^®^	Micelle	Cyclosporine A	Dry eye disease	2018
Inveltys^®^	Nanosuspension	Loteprednol etabonate	Postoperative ocular inflammation and pain	2018
Xelpros^®^	Nanoemulsion	Latanopros	Open-angle glaucoma or high intraocular pressure	2018
Eysuvis^®^	Nanosuspension	Loteprednol etabonate	Dry eye disesae	2020
Verkazia^®^	Nanoemulsion	Cyclosporine	Vernal keratoconjunctivitis	2021
Cyclokat^®^	Nanoemulsion	Cyclosporine A	Dry eye disease	NA
Lacrisek^®^	Liposomal spray	Vitamin A, E	Dry eye disease	NA
Artelac Rebalance^®^	Liposomal eye drops	Vitamin B12	Dry eye disease	NA

For example, Restasis^®^ was the first cyclosporine A (CsA) oil-in-water emulsion approved by the FDA for the treatment of DED in 2002 [337]. It used polysorbate-80 as an emulsifier and 0.5 mg/ml CsA was dissolved in castor oil. Importantly, the preservative-free emulsions (particle size 100–200 nm) effectively avoided the toxicity shown by earlier preservative-containing formulations. Nevertheless, Restasis^®^ is still accompanied by side effects such as epiphora, eye irritation and instillation pain [338].

Besides, Cequa^®^ is a nano-micellar formulation containing 0.09% CsA that is designed to improve drug delivery and penetration to ocular tissues. Cequa^®^ was approved by the FDA in 2018 for the treatment of DED [339]. The micellar formulation is composed of poly-oxygenated hydrogenated castor oil and octoxynol-40, which could form thermally stable micelles simultaneously by hydrogen bonding. The micelles have a particle size of 12–20 nm and a strong encapsulation ability to increase the CsA concentration tenfold [149]. In addition, Restasis^®^(CsA), Eysuvis^®^ (loteprednol etabonate), Lacrisek^®^ (vitamin A palmitate and vitamin E), Cyclokat^®^(CsA) and Artelac Rebalance^®^ (vitamin B12) are also used for the therapy of DED [22, 23].

Ikervis^®^ was introduced in 2015 for the treatment of severe keratitis [340]. Xelpros^®^ can be used to treat glaucoma or ocular hypertension [341]. Verkazia^®^ and Besivance^®^ can be used for vernal keratoconjunctivitis and allergic conjunctivitis/keratitis, respectively [341, 342].

Ozurdex^®^ contains a PLGA polymer matrix that provides long-term release of dexamethasone for up to 6 months. It was approved by the FDA in June 2009 for the treatment of macular edema [343, 344]. Bromsite^®^ [345] and Eysuvis^®^ [346], which were based on Durasite technology and mucus penetrating particle technology respectively, extended the residence time of drugs and improved treatment efficiency.

In addition, as shown in Table 3, many nano-based ocular drug delivery systems are currently in clinical testing stage, which further promote the delivery and development of ophthalmic drugs. Although the approval of nanocarriers has progressed slowly over the past two decades, more nanocarriers, including ocular nanomedicines, are expected to be available on the market in the near future.

**Table 3:** Representative nanomedicines in clinical trials for treating ocular diseases

Products/Drugs	Nanoformulation	Indications	Trials	Phase
Cyclosporine OTX-101	Nanomicelles	Dry eye disease	NCT02845674	III
AXR-159 ophthalmic	Micelles	Dry eye disease	NCT03598699	II
ISV-303	Micelles	Post cataract surgery inflammation	NCT01576952	III
ISV-305	Micelles	Post cataract surgery inflammation	NCT03192137	III
Dexamethasone (OCS-01)	Nanoparticles	Inflammation, corneal pain	NCT04130802	II
Urea (Pluronic® F-127)	Nanoparticles	Cataracts	NCT03001466	II
Paclitaxel	Nanoparticles	Intraocular melanoma	NCT00738361	II
Liposomal latanoprost	Nanoparticles	Ocular hypertension	NCT01987323	I/II
SeeQ CdSe 655	Nanoparticles	Retinitis pigmentosa	NCT04008771	NA
Dexamethasone (Cyclodextrin NP)	Nanoparticles	Diabetic macular edema	NCT01523314	II/III
GB-102	Microparticles	Wet AMD	NCT03953079	II
Dexamethasone cyclodextrin	Microparticles	Diabetic macular edema	NCT01523314	II
Brimonidine tartrate	Nanoemulsion	Cataract	NCT04246801	III
Clobetasol propionate	Nanoemulsion	Graft-vs Host disease (oGVHD)	NCT03591874	III
OCU-310	Nanoemulsion	Meibomian gland dysfunction	NCT03785340	III
TJO-087	Nanoemulsion	Dry eye disease	NCT05245604	III
Catioprost	Nanoemulsion	Glaucoma	NCT01254370	II
Omega-3 Fatty acids (Remogen® Omega)	Microemulsion	Dry eye disease	NCT02908282	NA
Difluprednate (PRO-145)	Emulsion	Cataract	NCT03693989	III
KPI-121 (1% and 0.25% loteprednol etabonate)	Submicron suspension (PRINT technology)	Ocular infection, and inflammation	NCT00849537	I, II
KPI-121 (1% and 0.25% loteprednol etabonate)	Submicron suspension (PRINT technology)	Dry eye disease and keratoconjunctivitis sicca	NCT02163824	III
Triamcinolone	Microsphere	Diabetic macular edema	NCT02813265	III
D-4517.2	Dendrimers	AMD	NCT05387837	II
TLC399 (Pro Dex)	Liposomes	Diabetic macular edema	NCT03093701	II
LAMELLEYE	Liposomes	Dry eye disease	NCT03140111	NA
Phospholipid (Artificial tears)	Liposomes	Dry eye disease	NCT02420834	NA
Liposomal latanoprost	Liposomes	Glaucoma	NCT01987323	I/II
Latanoprost (POLAT-001)	Liposomes	Ocular hypertension, open-angle glaucoma	NCT02466399	II
Hyaluronic acid	Liposomes	Meibomian gland dysfunction	NCT03617315	NA
Vincristine	Liposomes	Metastatic malignant uveal melanoma	NCT00506142	II
Marqibo	Liposomes	Retinoblastoma	NCT00335738	III
ENV 515 travoprost extended release (XR)	Intracameral implant (PRINT technology)	Glaucoma, ocular hypertension	NCT02371746	II
AR-1105 (dexamethasone)	Intravitreal implant	Macular edema	NCT03739593	II
AR-13503	Intravitreal implant	Neovascular AMD, diabetic macular edema	NCT03835884	I

To treat cataracts, a recent Phase II clinical trial (NCT03001466) involving in evaluating the therapeutic effect of a urea-loaded nanoparticulate system were conducted. Polymeric nanoparticles composed of Pluronic^®^ F-127 copolymers were used to enhance urea efficacy. In this clinical trial, patients in each group received either urea nanoparticles or balanced salt solution, with one drop of eye solution, five times a day for 8 weeks, and the scores of differences in 6-month visual acuity were measured [347].

INVELTYS are delivered as mucus penetrating particles for the treatment of postoperative inflammation and pain following eye surgery. The primary results of the clinical trial showed that INVELTYS, administered twice daily for 2 weeks, safely and effectively resolved postoperative ocular inflammation and subject-rated ocular pain after cataract surgery. The observed outcomes could be attributed to mucus penetrating particles that enable the drug to penetrate the tear film efficiently, facilitating drug release into targeting tissues [348].

Besides, in a recent Phase II clinical trial (NCT02466399), 80 participants with high IOP and open-angle glaucoma were recruited. The differences in intraocular pressure were measured after 3 months of treatment to compare the efficacy and safety of liposome latanoprost (POLAT-001) and latanoprost eye drops [349].

Recently, a multi-center open-labeled study (NCT02371746) is underway to evaluate the efficacy and safety of ENV 515 (travoprost) for treating ocular hypertension and glaucoma. AR-13503 (NCT03835884) and AR-1105 (NCT03739593) designed using PRINT technology as intravitreal implants for the treating AMD and DR are also in clinical trials [22].

## Recent patents on ocular disease therapy

The application and approval of a patent is the final confirmation of the commercial interest in a particular product. In the past years, researchers and pharmaceutical companies have made great progress in developing ocular drug delivery and have obtained multiple patents. Table 4 lists some representative patents in nano-based ocular drug delivery systems.

**Table 4:** Recent patents on nano-based ocular drug delivery systems

Patent title/description	Patent no	Disease targeted	Year	References
Seed crystal nanoparticles tetrandrine ophthalmic formulation and preparation method	CN105726484B	Cataract	2016	[350]
Puerarin and scutellarin lipid nanoparticle ophthalmic preparation and preparation method thereof	CN108066315A	Cataract	2016	[351]
Timolol maleate cubic liquid crystal nanoparticle eye drops and preparation method thereof	CN106619573A	Improved corneal infiltration capacity	2016	[352]
Ophthalmic nanoemulsion composition containing cyclosporin and method for preparing same	PH12015502587B1	Dry eye disease	2016	[353]
Cyclosporine containing nonirritative nanoemulsion ophthalmic composition	US 9,320,801 B2	Dry eye disease	2016	[354]
Nanosuspension of tobramycin and dexamethasone and preparation method thereof	CN105708844A	DED, Conjunctivitis, and tingling sensation in the eye	2016	[355]
Treatment of glaucoma and/or retinal diseases	WO2017152129A2	Glaucoma and retinal diseases	2017	[356]
Method for regulating retinal endothelial cell viability	US 9,566,255	Pathological conditions of retinal endothelial cells	2017	[357]
Pharmaceutical nanoparticles showing improved mucosal transport	AU2013256092B2	Aid in the transportation of Low aqueous soluble drugs in front of the eye	2017	[358]
Nanoparticle ophthalmic composition for the treatment of ocular disorders or diseases	US20190070242A1	Tear dysfunction syndrom	2018	[359]
Non-irritating nanoemulsion eye drop composition containing cyclosporine	US 15/747,618	Dry eye disease	2018	[360]
Method for regulating retinal endothelial cell viability	US 10,010,516 t	Pathological conditions of retinal endothelial cell	2018	[361]
Compositions of jasmonate compounds and methods of use	US20180000958 A1	Angiogenesis-related	2018	[362]
Ophthalmic compositions and methods of use	US20190008920A1	Disorders, inflammatory diseases	2018	[363]
Liposomes that contain at least one bilayer of lipid a prostaglandin F2α and phosphatidylcholine	US9956195B2	Dry eye syndrome	2018	[364]
A nanostructured biocompatible wafer which was used in conjuctival cul-de-sac	US9931306B2	Glaucoma, ocular surface disease	2018	[365]
The NPs, implants and microparticles are formed by the combination of polymer-drug	US20190070302A1	Wet AMD, uveitis	2019	[366]
Nanoparticles stabilized by nitrophenylboronic acid composition	JP2019108372A	For effective delivery of drugs to specific targets of eyes	2019	[367]
Drug delivery implant for treating eye diseases, and preparation method	WO2019160306A1	Ocular surface diseases	2019	[368]
Liposome corticosteroid for the locally injecting in inflammation lesion or region	CN109906075A	Inflammatory lesion in eye	2019	[369]
An oil-in-water nanoemulsion composition of clobetasol	WO2018233878A1	Inflammatory diseases or conditions	2019	[370]
Ophthalmic compositions and methods of use	WO2020047197A1	Dry eye disease related symptoms	2020	[371]
Eye composition containing cyclosporine and a method of preparing the same	KR20200000395A	Dry eye disease, conjunctivitis, and tingling sensation in the eye	2020	[372]
A surfactant-free type ophthalmic nanoemulsion composition and the manufacturing method thereo	KR20200053205A	Dry eye disease	2020	[373]
Nanocrystalline eye drop, preparation method, and application thereof	CN110664757A	Congenital macular degeneration	2020	[374]
Preservative free ocular compositions and methods for using same for treating dry eye disease and other eye disorders	US10751337B2	Dry eye disease and other eye disorders	2020	[375]

To treat cataracts, a patent (CN105726484B) disclosed a composition of tetrandrine liquid crystal nanoparticle eye-drops. The eye-drops were composed of matrix material, stabilizer, penetrating agent and cationic materials etc. Importantly, this invention has the advantages of strong drug loading capacity; good biocompatibility, high biological viscosity, higher stability and the capacity to improve the patient's compliance [350]. Likewise, Jialu et al. invented, a puerarin and scutellarin lipid nanoparticle ophthalmic preparation, with large membrane surface area and high drug carrying capacity. This patent (CN108066315A) opens up a new window for the treatment of cataract [351].

Peter et al. were patented for demonstrating that NPs, microparticles and implants are formed by polymer-drug a ssociations, with the ability to easily administer the required dose and deliver several drugs over extended periods of time (US20190070302A1). In addition, they disclosed that multiblock conjugates in the form of nonlinear copolymer-drug performed better and had fewer side effects compared to administration alone. The patent could be used to treat uveitis and wet AMD [366].

A patent (KR20200000395A) disclosed a composition of a nanoemulsion consisted of an active ingredient cyclosporine in a highly dissolved state and an emulsifier in an aqueous vehicle, which can improve the stability of medicine. The ideal particle size of nanoemulsion is 100 nm, with a narrow particle distribution. This application is effective for the treatment of DED, conjunctivitis and other ocular diseases [372].

Similarly, Application KR20200053205A provides a nano-emulsion eye drop consisting of cyclosporine, a solubilizer, a solvent and a stabilizer. Nanoemulsions have a droplet size of 20 nm or less, which facilitates penetration of the ocular barriers. Importantly, the nanoemulsion eye drop can improve stability and patient compliance, which are also used for the treatment of DED [373].

## Challenges and future perspectives

In this review, we first introduced the anatomy and barriers of the eye, where effective treatments and drug delivery are significant challenges due to the diversity of the diseases and the presence of ocular barriers, especially in the posterior segment of the eye. Although traditional drug administration has achieved certain efficacy in treating ocular diseases, some limitations remain, such as poor permeability, ineffective distribution, and insufficient bioavailability. Novel drug delivery methods, such as nanomicelles, NPs, nanosuspensions, microemulsions, dendrimers, liposomes, contact lenses, aqueous gels, MNs, and other novel drug delivery methods can significantly improve the efficacy of current treatment. At the same time, continued innovation in gene delivery and exosomes seems to be very exciting for drug delivery.

Despite some progress in developing novel ocular drug delivery systems, several challenges still exist. These include the complexity of production technology and processes, which limit the clinical translation of nanotechnology-based ocular drug delivery systems. Additionally, there is a need to improve the stability and safety of nanocarriers to minimize potential complications. Many new drug delivery techniques are primarily tested in animal experiments or in vitro studies, lacking comprehensive in vivo evaluations in human eyes. The targeting capabilities of nanocarriers need enhancement, and their metabolic fate within the eye remains unclear. Furthermore, these technologies' high technical requirements and manufacturing costs have hindered their commercial production and widespread clinical application. Addressing these challenges is crucial to advance ocular drug delivery and promoting its successful implementation in clinical practice.

In the future, more efforts should be paid to developing novel non-invasive ODDS that can overcome the ocular barriers, prolong the drug release time, and sustain therapeutical concentration at the lesion targets. Thus, nanocarriers' size, zeta potential, refractive index, safety, stability, pH, surface tension and osmotic pressure, of nanocarriers ought to be optimized. At the same time, more in vitro and in vivo experiments should be carried out, animal models more similar to human eye diseases should be established, and the evaluation methods of therapeutic effect should be further improved to better predict the safety and efficacy of delivery vectors. In addition, gene therapy, exosomes, and tissue engineering also provide new directions for ocular drug delivery.

In summary, the advantages of novel drug-delivery systems for ocular applications are undeniable, and these innovative nanocarriers will be increasingly used in clinical practice in the future.

## Data Availability

Not applicable.

## Abbreviations

AAV: Adeno-associated virus
AFM: Atomic force microscopy
AMD: Age-related macular degeneration
AmpB: Amphotericin B
ASO: Antisense oligonucleotide
aV: Anti-VEGF antibodies
BAB: Blood-aqueous barrier
BetP: Betamethasone phosphate
Bev: Bevacizumab
BRB: Blood-retinal barrier
BT: Brimonidine tartrate
CIP-NE: Ciprofloxacin-loaded nanoemulsion
CNV: Choroidal neovascularization
CsA: Cyclosporine A
CSO-VV-SA: Chitosan oligosaccharide-valylvaline-stearic acid
CS-PLGA NPs: Chitosan-coated polylactide-glycolic acid nanoparticles
DED: Dry eye disease
Dex-NW: Dexamethasone-loaded nanowafer
DHPs: Dendrimer gel hydrogel particles
DLS: Dynamic light scattering
DR: Diabetic retinopathy
D-TA: Dendrimer-triamcinolone acetonide
EV: Extracellular vesicle
EXO: Exosome
FDA: Food and drug administration
FF-TEM: Freeze-fracture transmission electron microscopy
FK: Fungal keratitis
HA: Hyaluronic acid
HA-LIP: Liposomes coated with hyaluronic acid
HCO-40/OC-40: Hydrogen-castor oil 40/octyl alcohol 40
HDR: Homology-directed repair
IOP: Intraocular pressure
IVT: Intravitreal injection
MEL: Melatonin
MEs: Microemulsions
MGO: Methylglyoxal
MNs: Microneedles
MOX-PAM: Moxifloxacin–pamoate
MSC-exo: Exosomes derived from mesenchymal stromal cells
NaMESys: Nanostructured microemulsions system
NaMESys-SOR: NaMESys carrying sorafenib
nEPC: Copolymer EPC
nEPCs + A: Aflibercept loaded nEPCs
NEs: Nanoemulsions
NHEJ: Non-homologous end joining mechanisms
NPs: Nanoparticles
NS: Nanosuspension
NS-TEM: Negative staining transmission electron microscopy
O/W: Oil-in-water
ODDS: Ocular drug delivery systems
PCL: Polycaprolactone
PDI: Polydispersion index
PEG: Polyethylene glycol
PLA: Polylactic acid
PLGA: Polylactide-coglycolide,
PUTK: Thioketal-containing polyurethane
PVA: Polyvinyl alcohol
rEXS: Exosomes derived from regulatory Treg cell
RH: Reactive oxygen -scavenging hydrogel
rMMNs: Redox-responsive quasi-mesoporous magnetic nanospheres
SEM: Scanning electron microscopy
shRNA: Short hairpin RNA
siRNA: Small interfering RNA
SNEDDS: Self-nano emulsifying drug delivery systems
SPM: Self-plug-type microneedle
TA: Triamcinolone acetonide
TEM: Transmission electron microscopy
TPGS: Tocopherol polyethylene glycol 1000 succinate
VEGF: Vascular endothelial growth factor
W/O: Water-in-oil
ZP: Zeta potential

## References

[1] Ma Y, Bao J, Zhang Y (2019). Mammalian near-infrared image vision through injectable and self-powered retinal nanoantennae. Cell.

[2] Gote V, Ansong M, Pal D (2020). Prodrugs and nanomicelles to overcome ocular barriers for drug penetration. Expert Opin Drug Metab Toxicol.

[3] Khiev D, Mohamed ZA, Vichare R (2021). Emerging nano-formulations and nanomedicines applications for ocular drug delivery. Nanomaterials (Basel).

[4] Kels BD, Grzybowski A, Grant-Kels JM (2015). Human ocular anatomy. Clin Dermatol.

[5] Nayak K, Misra M (2020). Triamcinolone acetonide-loaded PEGylated microemulsion for the posterior segment of eye. ACS Omega.

[6] Urtti A (2006). Challenges and obstacles of ocular pharmacokinetics and drug delivery. Adv Drug Deliv Rev.

[7] Tsai CH, Wang PY, Lin IC, Huang H, Liu GS, Tseng CL (2018). Ocular drug delivery: role of degradable polymeric nanocarriers for ophthalmic application. Int J Mol Sci.

[8] McCluskey P, Powell RJ (2004). The eye in systemic inflammatory diseases. Lancet.

[9] Vision impairment and blindness. https://www.who.int/news-room/fact-sheets/detail/blindness-and-visual-impairment Accessed 19 July 2022.

[10] Brown L, Leck AK, Gichangi M, Burton MJ, Denning DW (2021). The global incidence and diagnosis of fungal keratitis. Lancet Infect Dis.

[11] Wielders LHP, Schouten JSAG, Winkens B (2018). European multicenter trial of the prevention of cystoid macular edema after cataract surgery in nondiabetics: ESCRS PREMED study report 1. J Cataract Refract Surg.

[12] Kang JM, Tanna AP (2021). Glaucoma. Med Clin North Am.

[13] Rosenfeld PJ, Brown DM, Heier JS (2006). Ranibizumab for neovascular age-related macular degeneration. N Engl J Med.

[14] Stitt AW, Curtis TM, Chen M (2016). The progress in understanding and treatment of diabetic retinopathy. Prog Retin Eye Res.

[15] Cabrera FJ, Wang DC, Reddy K, Acharya G, Shin CS (2019). Challenges and opportunities for drug delivery to the posterior of the eye. Drug Discov Today.

[16] Jumelle C, Gholizadeh S, Annabi N, Dana R (2020). Advances and limitations of drug delivery systems formulated as eye drops. J Control Release.

[17] Ahmed S, Amin MM, Sayed S (2023). Ocular drug delivery: a comprehensive review. AAPS PharmSciTech.

[18] Al-Kinani AA, Zidan G, Elsaid N, Seyfoddin A, Alani AWG, Alany RG (2018). Ophthalmic gels: past, present and future. Adv Drug Deliv Rev.

[19] Silva B, São Braz B, Delgado E, Gonçalves L (2021). Colloidal nanosystems with mucoadhesive properties designed for ocular topical delivery. Int J Pharm.

[20] Gholizadeh S, Wang Z, Chen X, Dana R, Annabi N (2021). Advanced nanodelivery platforms for topical ophthalmic drug delivery. Drug Discov Today.

[21] Akhter MH, Ahmad I, Alshahrani MY (2022). Drug delivery challenges and current progress in nanocarrier-based ocular therapeutic system. Gels.

[22] Gorantla S, Rapalli VK, Waghule T (2020). Nanocarriers for ocular drug delivery: current status and translational opportunity. RSC Adv.

[23] Onugwu AL, Nwagwu CS, Onugwu OS (2023). Nanotechnology based drug delivery systems for the treatment of anterior segment eye diseases. J Control Release.

[24] Kang-Mieler JJ, Rudeen KM, Liu W, Mieler WF (2020). Advances in ocular drug delivery systems. Eye (Lond).

[25] Vaneev A, Tikhomirova V, Chesnokova N (2021). Nanotechnology for topical drug delivery to the anterior segment of the eye. Int J Mol Sci.

[26] Gupta A, Kafetzis KN, Tagalakis AD, Yu-Wai-Man C (2021). RNA therapeutics in ophthalmology—translation to clinical trials. Exp Eye Res.

[27] Adrianto MF, Annuryanti F, Wilson CG, Sheshala R, Thakur RRS (2022). In vitro dissolution testing models of ocular implants for posterior segment drug delivery. Drug Deliv Transl Res.

[28] Kumaran K, Karthika K, Padmapreetha J (2010). Comparative review on conventional and advanced ocular drug delivery formulations. Int J Pharm Pharm Sci.

[29] Patel A, Cholkar K, Agrahari V, Mitra AK (2013). Ocular drug delivery systems: an overview. World J Pharmacol.

[30] Bravo-Osuna I, Andrés-Guerrero V, Arranz-Romera A, Esteban-Pérez S, Molina-Martínez IT, Herrero-Vanrell R (2018). Microspheres as intraocular therapeutic tools in chronic diseases of the optic nerve and retina. Adv Drug Deliv Rev.

[31] Huang H, Yang XR, Li HL, Lu HS, Oswald J, Liu YM (2020). iRGD decorated liposomes: a novel actively penetrating topical ocular drug delivery strategy. Nano Res.

[32] Morrison PW, Khutoryanskiy VV (2014). Advances in ophthalmic drug delivery. Ther Deliv.

[33] Pflugfelder SC, Stern ME (2020). Biological functions of tear film. Exp Eye Res.

[34] Imperiale JC, Acosta GB, Sosnik A (2018). Polymer-based carriers for ophthalmic drug delivery. J Control Release.

[35] Wels M, Roels D, Raemdonck K, De Smedt SC, Sauvage F (2021). Challenges and strategies for the delivery of biologics to the cornea. J Control Release.

[36] Durairaj C (2017). Ocular pharmacokinetics. Handb Exp Pharmacol.

[37] Bachu RD, Chowdhury P, Al-Saedi ZHF, Karla PK, Boddu SHS (2018). Ocular drug delivery barriers-role of nanocarriers in the treatment of anterior segment ocular diseases. Pharmaceutics.

[38] Agrahari V, Mandal A, Agrahari V (2016). A comprehensive insight on ocular pharmacokinetics. Drug Deliv Transl Res.

[39] Kim YC, Chiang B, Wu X, Prausnitz MR (2014). Ocular delivery of macromolecules. J Control Release.

[40] Eghrari AO, Riazuddin SA, Gottsch JD (2015). Overview of the cornea: structure, function, and development. Prog Mol Biol Transl Sci.

[41] Gaudana R, Ananthula HK, Parenky A, Mitra AK (2010). Ocular drug delivery. AAPS J.

[42] Janagam DR, Wu L, Lowe TL (2017). Nanoparticles for drug delivery to the anterior segment of the eye. Adv Drug Deliv Rev.

[43] Zhang T, Xiang CD, Gale D, Carreiro S, Wu EY, Zhang EY (2008). Drug transporter and cytochrome P450 mRNA expression in human ocular barriers: implications for ocular drug disposition. Drug Metab Dispos.

[44] Kölln C, Reichl S (2012). mRNA expression of metabolic enzymes in human cornea, corneal cell lines, and hemicornea constructs. J Ocul Pharmacol Ther.

[45] Karla PK, Earla R, Boddu SH, Johnston TP, Pal D, Mitra A (2009). Molecular expression and functional evidence of a drug efflux pump (BCRP) in human corneal epithelial cells. Curr Eye Res.

[46] Ahmed S, Amin MM, El-Korany SM, Sayed S (2022). Corneal targeted fenticonazole nitrate-loaded novasomes for the management of ocular candidiasis: Preparation, *in vitro* characterization, ex vivo and in vivo assessments. Drug Deliv.

[47] Loftsson T, Stefánsson E (2017). Cyclodextrins and topical drug delivery to the anterior and posterior segments of the eye. Int J Pharm.

[48] Huang D, Chen YS, Rupenthal ID (2018). Overcoming ocular drug delivery barriers through the use of physical forces. Adv Drug Deliv Rev.

[49] Barar J, Javadzadeh AR, Omidi Y (2008). Ocular novel drug delivery: impacts of membranes and barriers. Expert Opin Drug Deliv.

[50] Bock F, Maruyama K, Regenfuss B (2013). Novel anti(lymph)angiogenic treatment strategies for corneal and ocular surface diseases. Prog Retin Eye Res.

[51] Shivhare R, Pathak A, Shrivastava N, Singh C, Tiwari G, Goyal R (2012). An update review on novel advancedocular drug delivery system. World J Pharm Pharm Sci.

[52] Watsky MA, Jablonski MM, Edelhauser HF (1988). Comparison of conjunctival and corneal surface areas in rabbit and human. Curr Eye Res.

[53] Ramsay E, Ruponen M, Picardat T (2017). Impact of chemical structure on conjunctival drug permeability: adopting porcine conjunctiva and cassette dosing for construction of in silico model. J Pharm Sci.

[54] Ahmed I, Gokhale RD, Shah MV, Patton TF (1987). Physicochemical determinants of drug diffusion across the conjunctiva, sclera, and cornea. J Pharm Sci.

[55] Gote V, Sikder S, Sicotte J, Pal D (2019). Ocular drug delivery: present innovations and future challenges. J Pharmacol Exp Ther.

[56] Rada JA, Shelton S, Norton TT (2006). The sclera and myopia. Exp Eye Res.

[57] Sun S, Li J, Li X (2016). Episcleral drug film for better-targeted ocular drug delivery and controlled release using multilayered poly-ε-caprolactone (PCL). Acta Biomater.

[58] Mofidfar M, Abdi B, Ahadian S (2021). Drug delivery to the anterior segment of the eye: a review of current and future treatment strategies. Int J Pharm.

[59] Coca-Prados M (2014). The blood-aqueous barrier in health and disease. J Glaucoma.

[60] Dubald M, Bourgeois S, Andrieu V, Fessi H (2018). Ophthalmic drug delivery systems for antibiotherapy-a review. Pharmaceutics.

[61] Singh M, Bharadwaj S, Lee KE, Kang SG (2020). Therapeutic nanoemulsions in ophthalmic drug administration: concept in formulations and characterization techniques for ocular drug delivery. J Control Release.

[62] Tisi A, Feligioni M, Passacantando M, Ciancaglini M, Maccarone R (2021). The impact of oxidative stress on blood-retinal barrier physiology in age-related macular degeneration. Cells.

[63] Díaz-Coránguez M, Ramos C, Antonetti DA (2017). The inner blood-retinal barrier: cellular basis and development. Vision Res.

[64] Duvvuri S, Majumdar S, Mitra AK (2003). Drug delivery to the retina: challenges and opportunities. Expert Opin Biol Ther.

[65] Bochot A, Couvreur P, Fattal E (2000). Intravitreal administration of antisense oligonucleotides: potential of liposomal delivery. Prog Retin Eye Res.

[66] Ge Y, Zhang A, Sun R (2020). Penetratin-modified lutein nanoemulsion *in-situ* gel for the treatment of age-related macular degeneration. Expert Opin Drug Deliv.

[67] Weinreb RN, Aung T, Medeiros FA (2014). The pathophysiology and treatment of glaucoma: a review. JAMA.

[68] Tham YC, Li X, Wong TY, Quigley HA, Aung T, Cheng CY (2014). Global prevalence of glaucoma and projections of glaucoma burden through 2040: a systematic review and meta-analysis. Ophthalmology.

[69] Gagnon MM, Boisjoly HM, Brunette I, Charest M, Amyot M (1997). Corneal endothelial cell density in glaucoma. Cornea.

[70] Li X, Zhang Z, Ye L (2017). Acute ocular hypertension disrupts barrier integrity and pump function in rat corneal endothelial cells. Sci Rep.

[71] Renner M, Stute G, Alzureiqi M (2017). Optic nerve degeneration after retinal ischemia/reperfusion in a rodent model. Front Cell Neurosci.

[72] Cardigos J, Ferreira Q, Crisóstomo S (2019). Nanotechnology-ocular devices for glaucoma treatment: a literature review. Curr Eye Res.

[73] Subrizi A, Del Amo EM, Korzhikov-Vlakh V, Tennikova T, Ruponen M, Urtti A (2019). Design principles of ocular drug delivery systems: importance of drug payload, release rate, and material properties. Drug Discov Today.

[74] Quigley HA (2019). 21st century glaucoma care. Eye (Lond).

[75] Wong WL, Su X, Li X (2014). Global prevalence of age-related macular degeneration and disease burden projection for 2020 and 2040: a systematic review and meta-analysis. Lancet Glob Health.

[76] Thomas CJ, Mirza RG, Gill MK (2021). Age-related macular degeneration. Med Clin North Am.

[77] Gopinath B, Wong TY (2018). Age-related macular degeneration. Lancet.

[78] Bakri SJ, Thorne JE, Ho AC (2019). Safety and efficacy of anti-vascular endothelial growth factor therapies for neovascular age-related macular degeneration: a report by the American academy of ophthalmology. Ophthalmology.

[79] Ogurtsova K, da Rocha Fernandes JD, Huang Y (2017). IDF Diabetes Atlas: global estimates for the prevalence of diabetes for 2015 and 2040. Diabetes Res Clin Pract.

[80] Cheung N, Mitchell P, Wong TY (2010). Diabetic retinopathy. Lancet.

[81] Tan TE, Wong TY (2023). Diabetic retinopathy: Looking forward to 2030. Front Endocrinol (Lausanne).

[82] Ajlan RS, Silva PS, Sun JK (2016). Vascular endothelial growth factor and diabetic retinal disease. Semin Ophthalmol.

[83] Madjedi K, Pereira A, Ballios BG (2022). Switching between anti-VEGF agents in the management of refractory diabetic macular edema: a systematic review. Surv Ophthalmol.

[84] Liu Y, Wu N (2021). Progress of nanotechnology in diabetic retinopathy treatment. Int J Nanomedicine.

[85] Pflugfelder SC, de Paiva CS (2017). The pathophysiology of dry eye disease: what we know and future directions for research. Ophthalmology.

[86] Craig JP, Nichols KK, Akpek EK (2017). TFOS DEWS II definition and classification report. Ocul Surf.

[87] Roda M, Corazza I, Bacchi Reggiani ML (2020). dry eye disease and tear cytokine levels-a meta-analysis. Int J Mol Sci.

[88] Asiedu K, Dzasimatu SK, Kyei S (2018). Impact of dry eye on psychosomatic symptoms and quality of life in a healthy youthful clinical sample. Eye Contact Lens.

[89] Na KS, Han K, Park YG, Na C, Joo CK (2015). Depression, stress, quality of life, and dry eye disease in Korean women: a population-based study. Cornea.

[90] Perez VL, Stern ME, Pflugfelder SC (2020). Inflammatory basis for dry eye disease flares. Exp Eye Res.

[91] Jones L, Downie LE, Korb D (2017). TFOS DEWS II management and therapy report. Ocul Surf.

[92] Wang L, Zhou MB, Zhang H (2021). The emerging role of topical ocular drugs to target the posterior eye. Ophthalmol Ther.

[93] Yang Y, Lockwood A (2022). Topical ocular drug delivery systems: Innovations for an unmet need. Exp Eye Res.

[94] Shen J, Lu GW, Hughes P (2018). Targeted ocular drug delivery with pharmacokinetic/pharmacodynamic considerations. Pharm Res.

[95] Maulvi FA, Shetty KH, Desai DT, Shah DO, Willcox MDP (2021). Recent advances in ophthalmic preparations: ocular barriers, dosage forms and routes of administration. Int J Pharm.

[96] Gause S, Hsu KH, Shafor C, Dixon P, Powell KC, Chauhan A (2016). Mechanistic modeling of ophthalmic drug delivery to the anterior chamber by eye drops and contact lenses. Adv Colloid Interface Sci.

[97] Grassiri B, Zambito Y, Bernkop-Schnürch A (2021). Strategies to prolong the residence time of drug delivery systems on ocular surface. Adv Colloid Interface Sci.

[98] O'Brien Laramy MN, Nagapudi K (2022). Long-acting ocular drug delivery technologies with clinical precedent. Expert Opin Drug Deliv.

[99] Raghava S, Hammond M, Kompella UB (2004). Periocular routes for retinal drug delivery. Expert Opin Drug Deliv.

[100] Le NT, Kroeger ZA, Lin WV, Khanani AM, Weng CY (2021). Novel treatments for diabetic macular edema and proliferative diabetic retinopathy. Curr Diab Rep.

[101] Barocas VH, Balachandran RK (2008). Sustained transscleral drug delivery. Expert Opin Drug Deliv.

[102] Chiang B, Jung JH, Prausnitz MR (2018). The suprachoroidal space as a route of administration to the posterior segment of the eye. Adv Drug Deliv Rev.

[103] Nayak K, Misra M (2018). A review on recent drug delivery systems for posterior segment of eye. Biomed Pharmacother.

[104] Liebmann JM, Barton K, Weinreb RN (2020). Evolving guidelines for intracameral injection. J Glaucoma.

[105] Gaballa SA, Kompella UB, Elgarhy O (2021). Corticosteroids in ophthalmology: drug delivery innovations, pharmacology, clinical applications, and future perspectives. Drug Deliv Transl Res.

[106] Lane SS, Osher RH, Masket S, Belani S (2008). Evaluation of the safety of prophylactic intracameral moxifloxacin in cataract surgery. J Cataract Refract Surg.

[107] Braga-Mele R, Chang DF, Henderson BA (2014). Intracameral antibiotics: safety, efficacy, and preparation. J Cataract Refract Surg.

[108] Labetoulle M, Findl O, Malecaze F (2016). Evaluation of the efficacy and safety of a standardised intracameral combination of mydriatics and anaesthetics for cataract surgery. Br J Ophthalmol.

[109] Behndig A, Cochener B, Güell JL (2013). Endophthalmitis prophylaxis in cataract surgery: overview of current practice patterns in 9 European countries. J Cataract Refract Surg.

[110] Grzybowski A, Brona P, Zeman L, Stewart MW (2021). Commonly used intracameral antibiotics for endophthalmitis prophylaxis: a literature review. Surv Ophthalmol.

[111] Keating GM (2013). Intracameral cefuroxime. Drugs.

[112] Ho JW, Afshari NA (2015). Advances in cataract surgery: preserving the corneal endothelium. Curr Opin Ophthalmol.

[113] Vazirani J, Basu S (2013). Role of topical, subconjunctival, intracameral, and irrigative antibiotics in cataract surgery. Curr Opin Ophthalmol.

[114] Del Amo EM, Rimpelä AK, Heikkinen E (2017). Pharmacokinetic aspects of retinal drug delivery. Prog Retin Eye Res.

[115] Jonas JB, Spandau UH, Schlichtenbrede F (2008). Short-term complications of intravitreal injections of triamcinolone and bevacizumab. Eye (Lond).

[116] Ilochonwu BC, Urtti A, Hennink WE, Vermonden T (2020). Intravitreal hydrogels for sustained release of therapeutic proteins. J Control Release.

[117] Tang Z, Fan X, Chen Y, Gu P (2022). Ocular Nanomedicine. Adv Sci (Weinh).

[118] Gross A, Cestari DM (2014). Optic neuropathy following retrobulbar injection: a review. Semin Ophthalmol.

[119] Alhassan MB, Kyari F, Ejere HO (2015). 2015 Peribulbar versus retrobulbar anaesthesia for cataract surgery. Cochrane Database Syst Rev.

[120] Hayashi K, Hayashi H (2005). Intravitreal versus retrobulbar injections of triamcinolone for macular edema associated with branch retinal vein occlusion. Am J Ophthalmol.

[121] Safi M, Ang MJ, Patel P, Silkiss RZ (2020). Rhino-orbital-cerebral mucormycosis (ROCM) and associated cerebritis treated with adjuvant retrobulbar amphotericin B. Am J Ophthalmol Case Rep.

[122] Cosgrove R, Rossow T, Cosgrove M, Siegel M (2020). Suspected systemic uptake of chlorpromazine after retrobulbar injection. Am J Ophthalmol Case Rep.

[123] Urtti A, Salminen L (1993). Minimizing systemic absorption of topically administered ophthalmic drugs. Surv Ophthalmol.

[124] Duncan TE (1983). Side effects of topical ocular timolol. Am J Ophthalmol.

[125] Anderson JA (1980). Systemic absorption of topical ocularly applied epinephrine and dipivefrin. Arch Ophthalmol.

[126] Inoue K (2014). Managing adverse effects of glaucoma medications. Clin Ophthalmol.

[127] Janoria KG, Gunda S, Boddu SH, Mitra AK (2007). Novel approaches to retinal drug delivery. Expert Opin Drug Deliv.

[128] Han H, Li S, Xu M (2023). Polymer- and lipid-based nanocarriers for ocular drug delivery: current status and future perspectives. Adv Drug Deliv Rev.

[129] Srinivasarao DA, Lohiya G, Katti DS (2019). Fundamentals, challenges, and nanomedicine-based solutions for ocular diseases. Wiley Interdiscip Rev Nanomed Nanobiotechnol.

[130] Grimaudo MA, Pescina S, Padula C (2019). Topical application of polymeric nanomicelles in ophthalmology: a review on research efforts for the noninvasive delivery of ocular therapeutics. Expert Opin Drug Deliv.

[131] Vaishya RD, Khurana V, Patel S, Mitra AK (2014). Controlled ocular drug delivery with nanomicelles. Wiley Interdiscip Rev Nanomed Nanobiotechnol.

[132] Hu Q, Rijcken CJ, van Gaal E (2016). Tailoring the physicochemical properties of core-crosslinked polymeric micelles for pharmaceutical applications. J Control Release.

[133] Bourzac K (2012). Nanotechnology: carrying drugs. Nature.

[134] Trivedi R, Kompella UB (2010). Nanomicellar formulations for sustained drug delivery: strategies and underlying principles. Nanomedicine (Lond).

[135] Torchilin VP (2001). Structure and design of polymeric surfactant-based drug delivery systems. J Control Release.

[136] Rangel-Yagui CO, Pessoa A, Tavares LC (2005). Micellar solubilization of drugs. J Pharm Pharm Sci.

[137] Wang Y, Jiang L, Shen Q, Shen J, Han Y, Zhang H (2017). Investigation on the self-assembled behaviors of C18 unsaturated fatty acids in arginine aqueous solution. RSC Adv.

[138] Fameau AL, Arnould A, Lehmann M, von Klitzing R (2015). Photoresponsive self-assemblies based on fatty acids. Chem Commun.

[139] Ghezzi M, Pescina S, Delledonne A (2021). Improvement of imiquimod solubilization and skin retention via TPGS micelles: exploiting the co-solubilizing effect of oleic acid. Pharmaceutics.

[140] Tampucci S, Guazzelli L, Burgalassi S (2020). pH-responsive nanostructures based on surface active fatty acid-protic ionic liquids for imiquimod delivery in skin cancer topical therapy. Pharmaceutics.

[141] Ghezzi M, Ferraboschi I, Delledonne A (2022). Cyclosporine-loaded micelles for ocular delivery: investigating the penetration mechanisms. J Control Release.

[142] Xu X, Sun L, Zhou L, Cheng Y, Cao F (2020). Functional chitosan oligosaccharide nanomicelles for topical ocular drug delivery of dexamethasone. Carbohydr Polym.

[143] Zhao X, Seah I, Xue K (2022). Antiangiogenic nanomicelles for the topical delivery of aflibercept to treat retinal neovascular disease. Adv Mater.

[144] Peng C, Kuang L, Zhao J, Ross AE, Wang Z, Ciolino JB (2022). Bibliometric and visualized analysis of ocular drug delivery from 2001 to 2020. J Control Release.

[145] Xu J, Zheng S, Hu X (2020). Advances in the research of bioinks based on natural collagen, polysaccharide and their derivatives for skin 3D bioprinting. Polymers (Basel).

[146] Akhter S, Anwar M, Siddiqui MA (2016). Improving the topical ocular pharmacokinetics of an immunosuppressant agent with mucoadhesive nanoemulsions: formulation development, in-vitro and in-vivo studies. Colloids Surf B Biointerfaces.

[147] Yetisgin AA, Cetinel S, Zuvin M, Kosar A, Kutlu O (2020). Therapeutic nanoparticles and their targeted delivery applications. Molecules.

[148] Sánchez-López E, Espina M, Doktorovova S, Souto EB, García ML (2017). Lipid nanoparticles (SLN, NLC): overcoming the anatomical and physiological barriers of the eye—Part I—Barriers and determining factors in ocular delivery. Eur J Pharm Biopharm.

[149] Meng T, Kulkarni V, Simmers R, Brar V, Xu Q (2019). Therapeutic implications of nanomedicine for ocular drug delivery. Drug Discov Today.

[150] Jiang C, Cano-Vega MA, Yue F (2022). Dibenzazepine-loaded nanoparticles induce local browning of white adipose tissue to counteract obesity. Mol Ther.

[151] Jiang C, Kuang L, Merkel MP (2015). Biodegradable polymeric microsphere-based drug delivery for inductive browning of fat. Front Endocrinol (Lausanne).

[152] Pandit J, Sultana Y, Aqil M (2021). Chitosan coated nanoparticles for efficient delivery of bevacizumab in the posterior ocular tissues via subconjunctival administration. Carbohydr Polym.

[153] Kim SN, Min CH, Kim YK (2022). Iontophoretic ocular delivery of latanoprost-loaded nanoparticles via skin-attached electrodes. Acta Biomater.

[154] Nguyen DD, Luo LJ, Lai JY (2020). Effects of shell thickness of hollow poly(lactic acid) nanoparticles on sustained drug delivery for pharmacological treatment of glaucoma. Acta Biomater.

[155] Schnichels S, Hurst J, de Vries JW (2021). Improved treatment options for glaucoma with brimonidine-loaded lipid DNA nanoparticles. ACS Appl Mater Interfaces.

[156] Chen Liangbo, Feng Wu, Pang Yan, Yan Dan, Zhang Siyi, Chen Fangjie, Nianxuan Wu, Gong Danni, Liu Jinyao, Yao Fu, Fan Xianqun (2021). Therapeutic nanocoating of ocular surface. Nano Today.

[157] Li M, Xu Z, Zhang L (2021). Targeted noninvasive treatment of choroidal neovascularization by hybrid cell-membrane-cloaked biomimetic nanoparticles. ACS Nano.

[158] Peltonen L, Hirvonen J (2018). Drug nanocrystals—versatile option for formulation of poorly soluble materials. Int J Pharm.

[159] Al-Kassas R, Bansal M, Shaw J (2017). Nanosizing techniques for improving bioavailability of drugs. J Control Release.

[160] Zhang J, Jiao J, Niu M (2021). Ten years of knowledge of nano-carrier based drug delivery systems in ophthalmology: current evidence, challenges, and future prospective. Int J Nanomed.

[161] Tai L, Liu C, Jiang K (2017). A novel penetratin-modified complex for noninvasive intraocular delivery of antisense oligonucleotides. Int J Pharm.

[162] Josyula A, Omiadze R, Parikh K (2021). An ion-paired moxifloxacin nanosuspension eye drop provides improved prevention and treatment of ocular infection. Bioeng Transl Med.

[163] García-Millán E, Quintáns-Carballo M, Otero-Espinar FJ (2017). Improved release of triamcinolone acetonide from medicated soft contact lenses loaded with drug nanosuspensions. Int J Pharm.

[164] Yan R, Xu L, Wang Q, Wu Z, Zhang H, Gan L (2021). Cyclosporine A nanosuspensions for ophthalmic delivery: a comparative study between cationic nanoparticles and drug-core mucus penetrating nanoparticles. Mol Pharm.

[165] Wu Y, Vora LK, Mishra D (2022). Nanosuspension-loaded dissolving bilayer microneedles for hydrophobic drug delivery to the posterior segment of the eye. Biomater Adv.

[166] Jacob S, Nair AB, Shah J (2020). Emerging role of nanosuspensions in drug delivery systems. Biomater Res.

[167] Rimple, Newton MJ (2018). Impact of ocular compatible lipoids and castor oil in fabrication of brimonidine tartrate nanoemulsions by 33 full factorial design. Recent Pat Inflamm Allergy Drug Discov.

[168] Qamar Z, Qizilbash FF, Iqubal MK (2019). Nano-based drug delivery system: recent strategies for the treatment of ocular disease and future perspective. Recent Pat Drug Deliv Formul.

[169] Singh Y, Meher JG, Raval K (2017). Nanoemulsion: concepts, development and applications in drug delivery. J Control Release.

[170] Lallemand F, Daull P, Benita S, Buggage R, Garrigue JS (2012). Successfully improving ocular drug delivery using the cationic nanoemulsion, novasorb. J Drug Deliv.

[171] Gupta A, Eral HB, Hatton TA, Doyle PS (2016). Nanoemulsions: formation, properties and applications. Soft Matter.

[172] Daull P, Lallemand F, Garrigue JS (2014). Benefits of cetalkonium chloride cationic oil-in-water nanoemulsions for topical ophthalmic drug delivery. J Pharm Pharmacol.

[173] Ammar HO, Salama HA, Ghorab M, Mahmoud AA (2009). Nanoemulsion as a potential ophthalmic delivery system for dorzolamide hydrochloride. AAPS PharmSciTech.

[174] Jurišić Dukovski B, Juretić M, Bračko D (2020). Functional ibuprofen-loaded cationic nanoemulsion: development and optimization for dry eye disease treatment. Int J Pharm.

[175] Tayel SA, El-Nabarawi MA, Tadros MI, Abd-Elsalam WH (2013). Promising ion-sensitive in situ ocular nanoemulsion gels of terbinafine hydrochloride: design, in vitro characterization and in vivo estimation of the ocular irritation and drug pharmacokinetics in the aqueous humor of rabbits. Int J Pharm.

[176] Mahboobian MM, Mohammadi M, Mansouri Z (2020). Development of thermosensitive in situ gel nanoemulsions for ocular delivery of acyclovir. J Drug Deliv Sci Technol.

[177] Bhalerao H, Koteshwara KB, Chandran S (2020). Design, optimisation and evaluation of in situ gelling nanoemulsion formulations of brinzolamide. Drug Deliv Transl Res.

[178] Youssef AAA, Cai C, Dudhipala N, Majumdar S (2021). Design of topical ocular ciprofloxacin nanoemulsion for the management of bacterial keratitis. Pharmaceuticals (Basel).

[179] Ismail A, Nasr M, Sammour O (2020). Nanoemulsion as a feasible and biocompatible carrier for ocular delivery of travoprost: improved pharmacokinetic/pharmacodynamic properties. Int J Pharm.

[180] Üstündag-Okur N, Gökçe EH, Eğrilmez S, Özer Ö, Ertan G (2014). Novel ofloxacin-loaded microemulsion formulations for ocular delivery. J Ocul Pharmacol Ther.

[181] Kale SN, Deore SL (2016). Emulsion micro emulsion and nano emulsion: a review. Syst Rev Pharm.

[182] Cunha Júnior AdS, Fialho SL, Carneiro LB, Oréfice F (2003). Microemulsions as drug delivery systems for topical ocular administration. Arquivos Brasileiros de Oftalmologia.

[183] Üstündağ Okur N, Er S, Çağlar E, Ekmen T, Sala F (2017). Formulation of microemulsions for dermal delivery of Cephalexin. Acta Pharm Sci.

[184] Mahran A, Ismail S, Allam AA (2021). Development of triamcinolone acetonide-loaded microemulsion as a prospective ophthalmic delivery system for treatment of uveitis: in vitro and in vivo evaluation. Pharmaceutics.

[185] Santonocito M, Zappulla C, Viola S (2021). Assessment of a new nanostructured microemulsion system for ocular delivery of sorafenib to posterior segment of the eye. Int J Mol Sci.

[186] Rupenthal ID, Agarwal P, Uy B (2022). Preparation and characterisation of a cyclodextrin-complexed mānuka honey microemulsion for eyelid application. Pharmaceutics.

[187] Deepak Amar, Goyal AK, Rath G (2018). Nanofiber in transmucosal drug delivery. J Drug Deliv Sci Technol.

[188] Razavi MS, Ebrahimnejad P, Fatahi Y, D'Emanuele A, Dinarvand R (2022). Recent developments of nanostructures for the ocular delivery of natural compounds. Front Chem.

[189] Hu X, Liu S, Zhou G, Huang Y, Xie Z, Jing X (2014). Electrospinning of polymeric nanofibers for drug delivery applications. J Control Release.

[190] Zupančič Š, Sinha-Ray S, Sinha-Ray S, Kristl J, Yarin AL (2016). Long-term sustained ciprofloxacin release from pmma and hydrophilic polymer blended nanofibers. Mol Pharm.

[191] Goyal R, Macri LK, Kaplan HM, Kohn J (2016). Nanoparticles and nanofibers for topical drug delivery. J Control Release.

[192] Da Silva GR, Lima TH, Fernandes-Cunha GM (2019). Ocular biocompatibility of dexamethasone acetate loaded poly(ɛ-caprolactone) nanofibers. Eur J Pharm Biopharm.

[193] Carracedo-Rodríguez G, Martínez-Águila A, Rodriguez-Pomar C, Bodas-Romero J, Sanchez-Naves J, Pintor J (2020). Effect of nutritional supplement based on melatonin on the intraocular pressure in normotensive subjects. Int Ophthalmol.

[194] Ferreira de Melo IM, Martins Ferreira CG, da Silva Lima, Souza EH (2020). Melatonin regulates the expression of inflammatory cytokines, VEGF and apoptosis in diabetic retinopathy in rats. Chem Biol Interact.

[195] Harpsøe NG, Andersen LP, Gögenur I, Rosenberg J (2015). Clinical pharmacokinetics of melatonin: a systematic review. Eur J Clin Pharmacol.

[196] Andersen LP, Werner MU, Rosenkilde MM (2016). Pharmacokinetics of oral and intravenous melatonin in healthy volunteers. BMC Pharmacol Toxicol.

[197] Romeo A, Kazsoki A, Omer S (2023). Formulation and characterization of electrospun nanofibers for melatonin ocular delivery. Pharmaceutics.

[198] Rohde F, Walther M, Wächter J, Knetzger N, Lotz C, Windbergs M (2022). In-situ tear fluid dissolving nanofibers enable prolonged viscosity-enhanced dual drug delivery to the eye. Int J Pharm.

[199] Tawfik EA, Alshamsan A, Abul Kalam M (2021). In vitro and in vivo biological assessment of dual drug-loaded coaxial nanofibers for the treatment of corneal abrasion. Int J Pharm.

[200] Esentürk I, Erdal MS, Güngör S (2016). Electrospinning method to produce drug-loaded nanofibers for topical/transdermal drug delivery applications. J Fac Pharm Istanb Univ.

[201] Farokhi M, Mottaghitalab F, Reis RL, Ramakrishna S, Kundu SC (2020). Functionalized silk fibroin nanofibers as drug carriers: advantages and challenges. J Control Release.

[202] Sridhar R, Lakshminarayanan R, Madhaiyan K, Amutha Barathi V, Lim KH, Ramakrishna S (2015). Electrosprayed nanoparticles and electrospun nanofibers based on natural materials: applications in tissue regeneration, drug delivery and pharmaceuticals. Chem Soc Rev.

[203] Yaylaci S, Dinç E, Aydın B, Tekinay AB, Guler MO (2023). Peptide nanofiber system for sustained delivery of anti-vegf proteins to the eye vitreous. Pharmaceutics.

[204] Shi X, Zhou T, Huang S (2023). An electrospun scaffold functionalized with a ROS-scavenging hydrogel stimulates ocular wound healing. Acta Biomater.

[205] Wei S, Yin R, Tang T (2019). Gas-permeable, irritation-free, transparent hydrogel contact lens devices with metal-coated nanofiber mesh for eye interfacing. ACS Nano.

[206] Abbasi E, Aval SF, Akbarzadeh A (2014). Dendrimers: synthesis, applications, and properties. Nanoscale Res Lett.

[207] Kambhampati SP, Kannan RM (2013). Dendrimer nanoparticles for ocular drug delivery. J Ocul Pharmacol Ther.

[208] Spataro G, Malecaze F, Turrin CO (2010). Designing dendrimers for ocular drug delivery. Eur J Med Chem.

[209] Shaikh A, Kesharwani P, Gajbhiye V (2022). Dendrimer as a momentous tool in tissue engineering and regenerative medicine. J Control Release.

[210] Romanowski EG, Yates KA, Paull JRA, Heery GP, Shanks RMQ (2021). Topical astodrimer sodium, a non-toxic polyanionic dendrimer, demonstrates antiviral activity in an experimental ocular adenovirus infection model. Molecules.

[211] Kambhampati SP, Bhutto IA, Wu T (2021). Systemic dendrimer nanotherapies for targeted suppression of choroidal inflammation and neovascularization in age-related macular degeneration. J Control Release.

[212] Wang J, Li B, Huang D (2021). Nano-in-nano dendrimer gel particles for efficient topical delivery of antiglaucoma drugs into the eye. Chem Eng J.

[213] Ge X, Wei M, He S, Yuan WE (2019). Advances of non-ionic surfactant vesicles (niosomes) and their application in drug delivery. Pharmaceutics.

[214] Keam SJ, Scott LJ, Curran MP (2003). Verteporfin: a review of its use in the management of subfoveal choroidal neovascularisation. Drugs.

[215] Tavakoli S, Peynshaert K, Lajunen T (2020). Ocular barriers to retinal delivery of intravitreal liposomes: impact of vitreoretinal interface. J Control Release.

[216] Kaur IP, Garg A, Singla AK, Aggarwal D (2004). Vesicular systems in ocular drug delivery: an overview. Int J Pharm.

[217] Lajunen T, Nurmi R, Kontturi L (2016). Light activated liposomes: functionality and prospects in ocular drug delivery. J Control Release.

[218] Chen X, Wu J, Lin X (2022). Tacrolimus loaded cationic liposomes for dry eye treatment. Front Pharmacol.

[219] Sahoo SK, Dilnawaz F, Krishnakumar S (2008). Nanotechnology in ocular drug delivery. Drug Discov Today.

[220] Chen S, Hanning S, Falconer J, Locke M, Wen J (2019). Recent advances in non-ionic surfactant vesicles (niosomes): Fabrication, characterization, pharmaceutical and cosmetic applications. Eur J Pharm Biopharm.

[221] Gan L, Wang J, Jiang M (2013). Recent advances in topical ophthalmic drug delivery with lipid-based nanocarriers. Drug Discov Today.

[222] Verma A, Tiwari A, Saraf S, Panda PK, Jain A, Jain SK (2021). Emerging potential of niosomes in ocular delivery. Expert Opin Drug Deliv.

[223] Farha AK, Gan RY, Li HB (2022). The anticancer potential of the dietary polyphenol rutin: current status, challenges, and perspectives. Crit Rev Food Sci Nutr.

[224] Wichayapreechar P, Anuchapreeda S, Phongpradist R, Rungseevijitprapa W, Ampasavate C (2020). Dermal targeting of *Centella asiatica* extract using hyaluronic acid surface modified niosomes. J Liposome Res.

[225] Kattar A, Quelle-Regaldie A, Sánchez L, Concheiro A, Alvarez-Lorenzo C (2023). Formulation and characterization of epalrestat-loaded polysorbate 60 cationic niosomes for ocular delivery. Pharmaceutics.

[226] Allam A, Elsabahy M, El Badry M, Eleraky NE (2021). Betaxolol-loaded niosomes integrated within pH-sensitive in situ forming gel for management of glaucoma. Int J Pharm.

[227] Fathalla D, Fouad EA, Soliman GM (2020). Latanoprost niosomes as a sustained release ocular delivery system for the management of glaucoma. Drug Dev Ind Pharm.

[228] Coursey TG, Henriksson JT, Marcano DC (2015). Dexamethasone nanowafer as an effective therapy for dry eye disease. J Control Release.

[229] Marcano DC, Shin CS, Lee B (2016). Synergistic cysteamine delivery nanowafer as an efficacious treatment modality for corneal cystinosis. Mol Pharm.

[230] Yuan X, Marcano DC, Shin CS (2015). Ocular drug delivery nanowafer with enhanced therapeutic efficacy. ACS Nano.

[231] Dourado LFN, da Silva CN, Gonçalves RS (2022). Improvement of PnPP-19 peptide bioavailability for glaucoma therapy: design and application of nanowafers based on PVA. J Drug Deliv Sci Technol.

[232] Rykowska I, Nowak I, Nowak R (2021). Soft contact lenses as drug delivery systems: a review. Molecules.

[233] Peral A, Martinez-Aguila A, Pastrana C, Huete-Toral F, Carpena-Torres C, Carracedo G (2020). Contact lenses as drug delivery system for glaucoma: a review. Appl Sci.

[234] Filipe HP, Henriques J, Reis P, Silva PC, Quadrado MJ, Serro AP (2016). Contact lenses as drug controlled release systems: a narrative review. Rev Bras Oftalmol.

[235] Choi SW, Kim J (2018). Therapeutic contact lenses with polymeric vehicles for ocular drug delivery: a review. Materials (Basel).

[236] Hsu KH, Carbia BE, Plummer C, Chauhan A (2015). Dual drug delivery from vitamin E loaded contact lenses for glaucoma therapy. Eur J Pharm Biopharm.

[237] Soeken TA, Ross AE, Kohane DS (2021). Dexamethasone-eluting contact lens for the prevention of postphotorefractive keratectomy scar in a New Zealand white rabbit model. Cornea.

[238] Maulvi FA, Soni TG, Shah DO (2016). A review on therapeutic contact lenses for ocular drug delivery. Drug Deliv.

[239] Shayani Rad M, Sabeti Z, Mohajeri SA, Fazly Bazzaz BS (2020). Preparation, characterization, and evaluation of zinc oxide nanoparticles suspension as an antimicrobial media for daily use soft contact lenses. Curr Eye Res.

[240] Bin Sahadan MY, Tong WY, Tan WN (2019). Phomopsidione nanoparticles coated contact lenses reduce microbial keratitis causing pathogens. Exp Eye Res.

[241] Jiao Z, Huo Q, Lin X (2022). Drug-free contact lens based on quaternized chitosan and tannic acid for bacterial keratitis therapy and corneal repair. Carbohydr Polym.

[242] Ding X, Ben-Shlomo G, Que L (2020). Soft contact lens with embedded microtubes for sustained and self-adaptive drug delivery for glaucoma treatment. ACS Appl Mater Interfaces.

[243] Cooper RC, Yang H (2019). Hydrogel-based ocular drug delivery systems: emerging fabrication strategies, applications, and bench-to-bedside manufacturing considerations. J Control Release.

[244] Irimia T, Dinu-Pîrvu CE, Ghica MV (2018). Chitosan-based in situ gels for ocular delivery of therapeutics: a state-of-the-art review. Mar Drugs.

[245] Sacco P, Furlani F, De Marzo G, Marsich E, Paoletti S, Donati I (2018). Concepts for developing physical gels of chitosan and of chitosan derivatives. Gels.

[246] Zhang Z, Ai S, Yang Z, Li X (2021). Peptide-based supramolecular hydrogels for local drug delivery. Adv Drug Deliv Rev.

[247] Arranz-Romera A, Esteban-Pérez S, Garcia-Herranz D, Aragón-Navas A, Bravo-Osuna I, Herrero-Vanrell R (2019). Combination therapy and co-delivery strategies to optimize treatment of posterior segment neurodegenerative diseases. Drug Discov Today.

[248] Lin S, Ge C, Wang D (2019). Overcoming the anatomical and physiological barriers in topical eye surface medication using a peptide-decorated polymeric micelle. ACS Appl Mater Interfaces.

[249] Fang G, Wang Q, Yang X, Qian Y, Zhang G, Tang B (2022). γ-Cyclodextrin-based polypseudorotaxane hydrogels for ophthalmic delivery of flurbiprofen to treat anterior uveitis. Carbohydr Polym.

[250] Jung JH, Kim SS, Chung H, Hejri A, Prausnitz MR (2022). Six-month sustained delivery of anti-VEGF from in-situ forming hydrogel in the suprachoroidal space. J Control Release.

[251] Gao H, Chen M, Liu Y (2023). Injectable anti-inflammatory supramolecular nanofiber hydrogel to promote anti-VEGF therapy in age-related macular degeneration treatment. Adv Mater.

[252] Lee K, Goudie MJ, Tebon P (2020). Non-transdermal microneedles for advanced drug delivery. Adv Drug Deliv Rev.

[253] Zhu J, Zhou X, Kim HJ (2020). Gelatin methacryloyl microneedle patches for minimally invasive extraction of skin interstitial fluid. Small.

[254] Jiang J, Moore JS, Edelhauser HF, Prausnitz MR (2009). Intrascleral drug delivery to the eye using hollow microneedles. Pharm Res.

[255] Gupta P, Yadav KS (2019). Applications of microneedles in delivering drugs for various ocular diseases. Life Sci.

[256] Shi H, Zhou J, Wang Y (2022). A rapid corneal healing microneedle for efficient ocular drug delivery. Small.

[257] Cui M, Zheng M, Wiraja C (2021). Ocular delivery of predatory bacteria with cryomicroneedles against eye infection. Adv Sci (Weinh).

[258] Lee K, Park S, Jo DH (2022). Self-plugging microneedle (SPM) for intravitreal drug delivery. Adv Healthc Mater.

[259] Tawfik M, Chen F, Goldberg JL, Sabel BA (2022). Nanomedicine and drug delivery to the retina: current status and implications for gene therapy. Naunyn Schmiedebergs Arch Pharmacol.

[260] Musarella MA (1992). Gene mapping of ocular diseases. Surv Ophthalmol.

[261] Cheng KJ, Hsieh CM, Nepali K, Liou JP (2020). Ocular disease therapeutics: design and delivery of drugs for diseases of the eye. J Med Chem.

[262] Mendell JR, Al-Zaidy SA, Rodino-Klapac LR (2021). Current clinical applications of in vivo gene therapy with AAVs. Mol Ther.

[263] Dunbar CE, High KA, Joung JK, Kohn DB, Ozawa K, Sadelain M (2018). Gene therapy comes of age. Science.

[264] Amador C, Shah R, Ghiam S, Kramerov AA, Ljubimov AV (2022). Gene therapy in the anterior eye segment. Curr Gene Ther.

[265] Ren W, Duan S, Dai C, Xie C, Jiang L, Shi Y (2023). Nanotechnology lighting the way for gene therapy in ophthalmopathy: from opportunities toward applications. Molecules.

[266] Colella P, Cotugno G, Auricchio A (2009). Ocular gene therapy: current progress and future prospects. Trends Mol Med.

[267] Naso MF, Tomkowicz B, Perry WL, Strohl WR (2017). Adeno-associated virus (AAV) as a vector for gene therapy. BioDrugs.

[268] Bastola P, Song L, Gilger BC, Hirsch ML (2020). Adeno-associated virus mediated gene therapy for corneal diseases. Pharmaceutics.

[269] Tarallo V, Bogdanovich S, Hirano Y (2012). Inhibition of choroidal and corneal pathologic neovascularization by Plgf1-de gene transfer. Invest Ophthalmol Vis Sci.

[270] Lu Y, Tai PWL, Ai J (2018). Transcriptome profiling of neovascularized corneas reveals miR-204 as a multi-target biotherapy deliverable by rAAVs. Mol Ther Nucleic Acids.

[271] Kaemmerer WF (2018). How will the field of gene therapy survive its success?. Bioeng Transl Med.

[272] Jiang J, Zhang X, Tang Y, Li S, Chen J (2021). Progress on ocular siRNA gene-silencing therapy and drug delivery systems. Fundam Clin Pharmacol.

[273] Del Amo EM, Urtti A (2008). Current and future ophthalmic drug delivery systems. A shift to the posterior segment. Drug Discov Today.

[274] Ma Y, Lin H, Wang P (2023). A miRNA-based gene therapy nanodrug synergistically enhances pro-inflammatory antitumor immunity against melanoma. Acta Biomater.

[275] Ribeiro MCS, de Miranda MC, Cunha PDS (2021). Neuroprotective effect of siRNA entrapped in hyaluronic acid-coated lipoplexes by intravitreal administration. Pharmaceutics.

[276] Kumar S, Fry LE, Wang JH (2023). RNA-targeting strategies as a platform for ocular gene therapy. Prog Retin Eye Res.

[277] Russell SR, Drack AV, Cideciyan AV (2022). Intravitreal antisense oligonucleotide sepofarsen in Leber congenital amaurosis type 10: a phase 1b/2 trial. Nat Med.

[278] Supe S, Upadhya A, Singh K (2021). Role of small interfering RNA (siRNA) in targeting ocular neovascularization: a review. Exp Eye Res.

[279] Wang J, Zhao P, Chen Z, Wang H, Wang Y, Lin Q (2023). Non-viral gene therapy using RNA interference with PDGFR-α mediated epithelial-mesenchymal transformation for proliferative vitreoretinopathy. Mater Today Bio.

[280] Dhurandhar D, Sahoo NK, Mariappan I, Narayanan R (2021). Gene therapy in retinal diseases: a review. Indian J Ophthalmol.

[281] Sander JD, Joung JK (2014). CRISPR-Cas systems for editing, regulating and targeting genomes. Nat Biotechnol.

[282] Guo N, Liu JB, Li W, Ma YS, Fu D (2022). The power and the promise of CRISPR/Cas9 genome editing for clinical application with gene therapy. J Adv Res.

[283] Gumerson JD, Alsufyani A, Yu W (2022). Restoration of RPGR expression in vivo using CRISPR/Cas9 gene editing. Gene Ther.

[284] Chung SH, Sin TN, Dang B (2022). CRISPR-based VEGF suppression using paired guide RNAs for treatment of choroidal neovascularization. Mol Ther Nucleic Acids.

[285] Banskota S, Raguram A, Suh S (2022). Engineered virus-like particles for efficient in vivo delivery of therapeutic proteins. Cell.

[286] Manukonda R, Attem J, Yenuganti VR, Kaliki S, Vemuganti GK (2022). Exosomes in the visual system: new avenues in ocular diseases. Tumour Biol.

[287] Gurung S, Perocheau D, Touramanidou L, Baruteau J (2021). The exosome journey: from biogenesis to uptake and intracellular signalling. Cell Commun Signal.

[288] Feng X, Peng Z, Yuan L (2023). Research progress of exosomes in pathogenesis, diagnosis, and treatment of ocular diseases. Front Bioeng Biotechnol.

[289] Wortzel I, Dror S, Kenific CM, Lyden D (2019). Exosome-mediated metastasis: communication from a distance. Dev Cell.

[290] Kalluri R, LeBleu VS (2020). The biology, function, and biomedical applications of exosomes. Science..

[291] Dong X, Lei Y, Yu Z (2021). Exosome-mediated delivery of an anti-angiogenic peptide inhibits pathological retinal angiogenesis. Theranostics.

[292] Tian Y, Zhang F, Qiu Y (2021). Reduction of choroidal neovascularization via cleavable VEGF antibodies conjugated to exosomes derived from regulatory T cells. Nat Biomed Eng.

[293] Zhou T, He C, Lai P (2022). miR-204-containing exosomes ameliorate GVHD-associated dry eye disease. Sci Adv.

[294] Herrmann IK, Wood MJA, Fuhrmann G (2021). Extracellular vesicles as a next-generation drug delivery platform. Nat Nanotechnol.

[295] Piffoux M, Silva AKA, Wilhelm C, Gazeau F, Tareste D (2018). Modification of extracellular vesicles by fusion with liposomes for the design of personalized biogenic drug delivery systems. ACS Nano.

[296] Kojima R, Bojar D, Rizzi G (2018). Designer exosomes produced by implanted cells intracerebrally deliver therapeutic cargo for Parkinson's disease treatment. Nat Commun.

[297] Siqueira Jørgensen SD, Al Sawaf M, Graeser K, Mu H, Müllertz A, Rades T (2018). The ability of two in vitro lipolysis models reflecting the human and rat gastro-intestinal conditions to predict the in vivo performance of SNEDDS dosing regimens. Eur J Pharm Biopharm.

[298] Pouton CW (2000). Lipid formulations for oral administration of drugs: non-emulsifying, self-emulsifying and 'self-microemulsifying' drug delivery systems. Eur J Pharm Sci.

[299] Ujhelyi Z, Vecsernyés M, Fehér P (2018). Physico-chemical characterization of self-emulsifying drug delivery systems. Drug Discov Today Technol.

[300] Li Z, Xu D, Yuan Y (2020). Advances of spontaneous emulsification and its important applications in enhanced oil recovery process. Adv Colloid Interface Sci.

[301] Buya AB, Beloqui A, Memvanga PB, Préat V (2020). Self-Nano-emulsifying drug-delivery systems: from the development to the current applications and challenges in oral drug delivery. Pharmaceutics.

[302] López-Cano JJ, González-Cela-Casamayor MA, Andrés-Guerrero V (2022). Development of an osmoprotective microemulsion as a therapeutic platform for ocular surface protection. Int J Pharm.

[303] Kontogiannidou E, Meikopoulos T, Gika H (2020). In vitro evaluation of self-nano-emulsifying drug delivery systems (SNEDDS) containing room temperature ionic liquids (RTILs) for the oral delivery of amphotericin B. Pharmaceutics.

[304] Whitesides GM (2005). Nanoscience, nanotechnology, and chemistry. Small.

[305] Zhang T, Wei C, Wu X (2023). Characterization and evaluation of rapamycin-loaded nano-micelle ophthalmic solution. J Funct Biomater.

[306] Barenholz Y (2012). Doxil®–the first FDA-approved nano-drug: lessons learned. J Control Release.

[307] Lai SK, Wang YY, Hanes J (2009). Mucus-penetrating nanoparticles for drug and gene delivery to mucosal tissues. Adv Drug Deliv Rev.

[308] Toropainen E, Fraser-Miller SJ, Novakovic D (2021). Biopharmaceutics of topical ophthalmic suspensions: importance of viscosity and particle size in ocular absorption of indomethacin. Pharmaceutics.

[309] Younes NF, Abdel-Halim SA, Elassasy AI (2018). Corneal targeted Sertaconazole nitrate loaded cubosomes: Preparation, statistical optimization, in vitro characterization, ex vivo permeation and in vivo studies. Int J Pharm.

[310] Bali V, Ali M, Ali J (2010). Study of surfactant combinations and development of a novel nanoemulsion for minimising variations in bioavailability of ezetimibe. Colloids Surf B Biointerfaces.

[311] Tamilvanan S, Benita S (2004). The potential of lipid emulsion for ocular delivery of lipophilic drugs. Eur J Pharm Biopharm.

[312] Apaolaza PS, Delgado D, del Pozo-Rodríguez A, Gascón AR, Solinís MÁ (2014). A novel gene therapy vector based on hyaluronic acid and solid lipid nanoparticles for ocular diseases. Int J Pharm.

[313] Fangueiro JF, Andreani T, Egea MA (2014). Design of cationic lipid nanoparticles for ocular delivery: development, characterization and cytotoxicity. Int J Pharm.

[314] Fahmy AM, Hassan M, El-Setouhy DA, Tayel SA, Al-Mahallawi AM (2021). Voriconazole ternary micellar systems for the treatment of ocular mycosis: statistical optimization and in vivo evaluation. J Pharm Sci.

[315] Balguri SP, Adelli GR, Janga KY, Bhagav P, Majumdar S (2017). Ocular disposition of ciprofloxacin from topical, PEGylated nanostructured lipid carriers: Effect of molecular weight and density of poly (ethylene) glycol. Int J Pharm.

[316] Nayak K, Misra M (2020). Triamcinolone acetonide-Loaded PEGylated microemulsion for the posterior segment of eye. ACS Omega.

[317] Lakhani P, Patil A, Wu KW (2019). Optimization, stabilization, and characterization of amphotericin B loaded nanostructured lipid carriers for ocular drug delivery. Int J Pharm.

[318] Craig JP, Simmons PA, Patel S, Tomlinson A (1995). Refractive index and osmolality of human tears. Optom Vis Sci.

[319] Patel N, Nakrani H, Raval M, Sheth N (2016). Development of loteprednol etabonate-loaded cationic nanoemulsified in-situ ophthalmic gel for sustained delivery and enhanced ocular bioavailability. Drug Deliv.

[320] Fialho SL, da Silva-Cunha A (2004). New vehicle based on a microemulsion for topical ocular administration of dexamethasone. Clin Exp Ophthalmol.

[321] López-Alemany A, Montés-Micó R, García-Valldecabres M (1999). Ocular physiology and artificial tears. J Am Optom Assoc.

[322] Moiseev RV, Steele F, Khutoryanskiy VV (2022). Polyaphron formulations stabilised with different water-soluble polymers for ocular drug delivery. Pharmaceutics.

[323] Radomska-Soukharev A, Wojciechowska J (2005). Microemulsions as potential ocular drug delivery systems: phase diagrams and physical properties depending on ingredients. Acta Pol Pharm.

[324] Doshi U, Xu J (2009). Effect of viscosity, surface tension and mucoadhesion on ocular residence time of lubricant eye drops. Invest Ophthalmol Vis Sci.

[325] Luo Q, Zhao J, Zhang X, Pan W (2011). Nanostructured lipid carrier (NLC) coated with Chitosan Oligosaccharides and its potential use in ocular drug delivery system. Int J Pharm.

[326] Stahl U, Willcox M, Stapleton F (2012). Osmolality and tear film dynamics. Clin Exp Optom.

[327] Murube J (2006). Tear osmolarity. Ocul Surf.

[328] Varela-Fernández R, Díaz-Tomé V, Luaces-Rodríguez A (2020). Drug delivery to the posterior segment of the eye: biopharmaceutic and pharmacokinetic considerations. Pharmaceutics.

[329] Shetty R, Naidu JR, Nair AP (2020). Distinct ocular surface soluble factor profile in human corneal dystrophies. Ocul Surf.

[330] Romeo A, Musumeci T, Carbone C (2021). Ferulic acid-loaded polymeric nanoparticles for potential ocular delivery. Pharmaceutics.

[331] Carnevale C, Riva I, Roberti G (2021). Confocal microscopy and anterior segment optical coherence tomography imaging of the ocular surface and bleb morphology in medically and surgically treated glaucoma patients: a review. Pharmaceuticals (Basel).

[332] Khalil IA, Ali IH, El-Sherbiny IM (2019). Noninvasive biodegradable nanoparticles-in-nanofibers single-dose ocular insert: in vitro, ex vivo and in vivo evaluation. Nanomedicine (Lond).

[333] Leonardi A, Bucolo C, Romano GL (2014). Influence of different surfactants on the technological properties and in vivo ocular tolerability of lipid nanoparticles. Int J Pharm.

[334] Ammar HO, Haider M, Ibrahim M, El Hoffy NM (2017). In vitro and in vivo investigation for optimization of niosomal ability for sustainment and bioavailability enhancement of diltiazem after nasal administration. Drug Deliv.

[335] Tavakoli M, Mahboobian MM, Nouri F, Mohammadi M (2021). Studying the ophthalmic toxicity potential of developed ketoconazole loaded nanoemulsion *in situ gel* formulation for ophthalmic administration. Toxicol Mech Methods.

[336] Mahboobian MM, Seyfoddin A, Aboofazeli R, Foroutan SM, Rupenthal ID (2019). Brinzolamide-loaded nanoemulsions: ex vivo transcorneal permeation, cell viability and ocular irritation tests. Pharm Dev Technol.

[337] Ames P, Galor A (2015). Cyclosporine ophthalmic emulsions for the treatment of dry eye: a review of the clinical evidence. Clin Investig (Lond).

[338] Boujnah Y, Mouchel R, El-Chehab H, Dot C, Burillon C, Kocaba V (2018). Étude prospective, monocentrique, non contrôlée de l’efficacité, de la tolérance et de l’adhésion au traitement par ciclosporine 0,1 % au cours des sécheresses oculaires sévères [Prospective, monocentric, uncontrolled study of efficacy, tolerance and adherence of cyclosporin 0.1 % for severe dry eye syndrome]. J Fr Ophtalmol.

[339] Mandal A, Gote V, Pal D, Ogundele A, Mitra AK (2019). Ocular Pharmacokinetics of a topical ophthalmic nanomicellar solution of cyclosporine (Cequa®) for dry eye disease. Pharm Res.

[340] Henostroza M, Melo K, Yukuyama MN, Löbenberg R, Bou-Chacra NA (2020). Cationic rifampicin nanoemulsion for the treatment of ocular tuberculosis. Colloids Surf, A.

[341] Kagkelaris K, Panayiotakopoulos G, Georgakopoulos CD (2022). Nanotechnology-based formulations to amplify intraocular bioavailability. Ther Adv Ophthalmol.

[342] Eroglu YI (2017). A comparative review of Haute Autorité de Santé and National Institute for Health and Care Excellence health technology assessments of Ikervis® to treat severe keratitis in adult patients with dry eye disease which has not improved despite treatment with tear substitutes. J Mark Access Health Policy.

[343] Boyer DS, Yoon YH, Belfort R (2014). Three-year, randomized, sham-controlled trial of dexamethasone intravitreal implant in patients with diabetic macular edema. Ophthalmology.

[344] Wentz SM, Price F, Harris A, Siesky B, Ciulla T (2019). Efficacy and safety of bromfenac 0.075% formulated in DuraSite for pain and inflammation in cataract surgery. Expert Opin Pharmacother.

[345] Rodrigues GA, Lutz D, Shen J (2018). Topical drug delivery to the posterior segment of the eye: addressing the challenge of preclinical to clinical translation. Pharm Res.

[346] Ahn SJ, Hong HK, Na YM (2016). Use of rabbit eyes in pharmacokinetic studies of intraocular drugs. J Vis Exp.

[347] U.S. National Library of Medicine, A randomized controlled trial comparing urea loaded nanoparticles to placebo: a new concept for cataract management, NCT03001466, 2016.

[348] Kim T, Sall K, Holland EJ, Brazzell RK, Coultas S, Gupta PK (2018). Safety and efficacy of twice daily administration of KPI-121 1% for ocular inflammation and pain following cataract surgery. Clin Ophthalmol.

[349] U.S. National Library of Medicine, POLAT-001 compared to latanoprost ophthalmic solution in patients with ocular hypertension and open-angle glaucoma, NCT02466399, 2020.

[350] Wang LR, Wang Y, Wang SLW, Jingjing JC, Xingguo H. Seed crystal nanoparticles tetrandrine ophthalmic formulation and preparation method. C.N. Patent CN 1,05,726,484 B, 2016.

[351] Jialu WLR, Ruijuan LWL, Ze ZFW. Puerarin and scutellarin lipid nanoparticle ophthalmic preparation and preparation method thereof. C.N. Patent CN 1,08,066,315 A, 2016.

[352] Li CY, Li YP, Ying WH, Hangping C. Timolol maleate cubic liquid crystal nanoparticle eye drops and preparation method thereof. C.N. Patent CN 1,06,619,573 A, 2016.

[353] Lee JY, Shin YJ, Sang-Rok R. ophthalmic nanoemulsion composition containing cyclosporine and method for preparing same, PH12015502587B1, 2016.

[354] Wang SJ, Cha KH, Kang H, Sun BK. Cyclosporine-containing non-irritative nanoemulsion ophthalmic composition, US 9,320,801 B2, 2016.

[355] XU S, Zhu Y, Fan Q, Ou S, Liu X. nanosuspension of tobramycin and dexamethasone and preparation method thereof, CN105708844, 2016.

[356] Weiss, S.L. Treatment of glaucoma and/or retinal diseases. WO 2017152129A2, 9 August 2017.

[357] Yates CR, Smith JS, Miller DD, Toutounchian JJ. Method for regulating retinal endothelial cell viability, in, US 9,566,255, 2017.

[358] Chen H, Enlow EM, Popov A. Pharmaceutical nanoparticles showing improved mucosal transport. A.U. Patent AU 2,013,256,092 B2, 2017.

[359] Campora G. Nanoparticle ophthalmic composition for the treatment of ocular disorders or diseases. U.S. Patent US 20,190,070,242 A1, 2018.

[360] Dongwoo L, Hyunju B, Younggwan K. Non-irritant ophthalmic composition containing cyclosporin, and convenient preparation method, US 15/747,618, 2018.

[361] Yates CR, Smith JS, Miller DD, Toutounchian JJ. Method for regulating retinal endothelial cell viability, in, US 10,010,516, 2018.

[362] Lopes FP, Jose E. Compositions of jasmonate compounds and methods of use. US 20,180,000,958 A1, 2018.

[363] Arumugham R, Upadhyay AK. Ophthalmic compositions and methods of use. U.S. Patent US 20,190,008,920 A1, 2018.

[364] Venkatraman S, Natarajan JV, Howden T, Boey F. inventors; Nanyang Technological University, Singapore Health Services Pte Ltd, assignee. Stable liposomal formulations for ocular drug delivery. United States patent US 9,956,195. 2018 May 1.

[365] Barman SP, Liu M, Barman K, Ward KL, Hackett B. inventors; Integral Biosystems LLC, assignee. Methods and biocompatible compositions to achieve sustained drug release in the eye. United States patent US 9,931,306. 2018 Apr 3.

[366] Fu J, Campochiaro PA, Hanes JS. inventors; Johns Hopkins University, assignee. Non-linear multiblock copolymer-drug conjugates for the delivery of active agents. United States patent application US 16/182,261. 2019 Mar 7.

[367] Davis ME, Davishan ME, Han H. Nanoparticles stabilized by nitrophenylboronic acid composition. JP 2,019,108,372A, 2019.

[368] Lee HC. Drug delivery implant for treating eye diseases, and preparation method therefore. WO 2,019,160,306A1, 2019.

[369] Liposome Corticosteroid for the Locally Injecting in Inflammation Lesion or Region. CN 109906075A, 18 June 2019.

[370] Aquilue JS, Gris MDCL, Gan˜an MID. ´ An oil-in-water nanoemulsion composition of clobetasol, in: WO2018233878A1, 2019.

[371] Rasappa Arumugham AU. Ophthalmic compositions and methods of use, in: WO2020047197A1, 2020.

[372] Junyeop L, Jae SY, Sang-rok R. Eye composition containing a cyclosporine and a method of preparing the same. KR20200000395A, 2 January 2020.

[373] Chul-hwan K, Hyun-seop N, Hye-min K, Da-hye S. A surfactant-free type ophthalmic nano-emulsion composition, and the manufacturing method thereof. KR 20200053205A, 18 May 2020.

[374] Qing D. Nanocrystalline eye drop, preparation method and application thereof. CN 110664757A, 28 May 2020.

[375] Jain S, Kompella UB, Musunuri S. Preservative free ocular compositions and methods for using the same for treating dry eye disease and other eye disorders, in: US10751337B2, 2020.

